# Xylazine as an emerging new psychoactive substance; focuses on both 5‐HT_7_ and κ‐opioid receptors' molecular interactions and isosteric replacement

**DOI:** 10.1002/ardp.202500041

**Published:** 2025-03-17

**Authors:** Giuseppe Floresta, Alberto Granzotto, Vincenzo Patamia, Davide Arillotta, Gabriele D. Papanti, Amira Guirguis, John M. Corkery, Giovanni Martinotti, Stefano L. Sensi, Fabrizio Schifano

**Affiliations:** ^1^ Psychopharmacology, Drug Misuse and Novel Psychoactive Substances Research Unit, School of Health, Medicine and Life Sciences University of Hertfordshire Hatfield United Kingdom; ^2^ Department of Drug and Health Sciences University of Catania Catania Italy; ^3^ Center for Advanced Studies and Technology – CAST University G. d'Annunzio of Chieti‐Pescara Chieti Italy; ^4^ Department of Neuroscience, Imaging, and Clinical Sciences University G. d'Annunzio of Chieti‐Pescara Chieti Italy; ^5^ Department of Neurosciences, Psychology, Drug Research, and Child Health, Section of Pharmacology and Toxicology University of Florence Florence Italy; ^6^ Tolmezzo Community Mental Health Centre, ASUFC Mental Health and Addiction Department Tolmezzo Italy; ^7^ Pharmacy, Swansea University Medical School Swansea University Swansea Wales United Kingdom; ^8^ Institute for Advanced Biomedical Technologies – ITAB University G. d'Annunzio of Chieti‐Pescara Chieti Italy; ^9^ Institute of Neurology, SS Annunziata University Hospital University G. d'Annunzio of Chieti‐Pescara Chieti Italy

**Keywords:** computational approaches, drug misuse, drug overdose, fentanyl, in silico studies

## Abstract

Xylazine, traditionally used as a veterinary sedative, has recently emerged as a new psychoactive substance, being typically ingested in combination with fentanyl derivatives and hence raising significant public health concerns. Despite its increasing prevalence, little is known about its molecular interactions with human neuroreceptors, specifically the serotonin 7 (5‐HT_7_R) and kappa‐opioid (KOR) receptors, which play critical roles in mood regulation, consciousness and nociception. Hence, the binding affinity and molecular interactions of xylazine with both 5‐HT_7_R and KOR through docking simulations and molecular dynamics calculations were investigated. These computational approaches revealed critical insights into receptor binding motifs and highlighted structural modifications that could enhance receptor affinity. The isosteric replacements within the xylazine structure to improve its binding efficacy were assessed, demonstrating that minimal structural modifications can potentiate its interaction with 5‐HT_7_R and KOR. These findings may well advance our understanding of xylazine's mechanism of action, possibly contributing to identifying suitable treatment/management approaches in treating xylazine‐related overdoses.

## Graphical abstract

The binding affinities of xylazine with 5‐HT_7_ and kappa‐opioid receptors are revealed through computational modeling, highlighting potential structural modifications to enhance receptor interaction, providing crucial insights for addressing xylazine‐related overdoses.

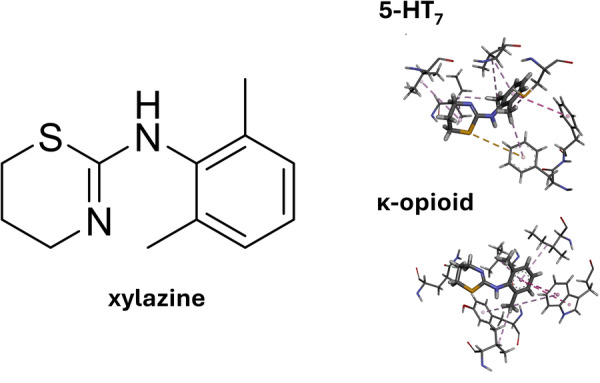

## 1 INTRODUCTION

The rise of new psychoactive substances (NPS) presents a unique challenge to public health, with many compounds designed to mimic the effects of controlled drugs whilst evading regulation.^[^ ^1^, ^2^ ^]^ Xylazine is a nonopioid sedative compound used for its preanesthetic, pain relief and muscle relaxation properties in veterinary medicine; it is indeed unlicensed for human use.^[^ ^3^, ^4^ ^]^ In veterinary medicine, xylazine is typically combined with other medications, such as ketamine. Xylazine has, however, recently emerged as an NPS in humans, being frequently combined with either synthetic opioids and/or psychostimulants, alcohol, or benzodiazepines.^[^ ^5^, ^6^ ^]^ Xylazine alone is commonly referred to as “tranq”; conversely, when combined with opiates/opioids such as heroin or fentanyl, the mixture is usually termed “tranq dope.”^[^ ^3^ ^]^

Acute xylazine intoxication is potentially life threatening, with the molecule likely acting as a central nervous system depressant and hence putatively potentiating the clinical effects of both opiates/opioids and remaining sedatives.^[^ ^7^ ^]^ Indeed, overdose deaths related to the combination of xylazine with illicit fentanyl rose in the United States during 2019‒2022,^[^ ^8^, ^9^ ^]^ with this drug combination being currently considered a public health threat.^[^ ^9^, ^10^ ^]^

In veterinary medicine, xylazine is meant to produce synergistic antinociceptive effects in combination with fentanyl.^[^ ^11^ ^]^ Conversely, preclinical studies confirmed xylazine potentiating fentanyl lethality risk.^[^ ^12^ ^]^ In humans, overdoses associated with xylazine may resemble opioid overdoses, but xylazine is not typically included in routine drug screening tests. Since it is not considered an opioid molecule, one could argue that its clinical effects may not be reversed by the opioid antagonist naloxone.^[^ ^13^ ^]^ However, recent preclinical data suggested that xylazine is a full agonist at kappa‐opioid receptors (KOR) and that naloxone precipitated withdrawal from both xylazine and fentanyl/xylazine coadministration, with enhanced sensitivity in females.^[^ ^14^ ^]^ Furthermore, chronic xylazine intravenous misuse may be associated with skin ulcers and related infections, whilst its related dependence and withdrawal issues in humans are still unclear (for a thorough review, see^[^ ^15^ ^]^).

From the pharmacological point of view, xylazine is a clonidine analogue, which closely resembles the chemical structure of phenothiazines and tricyclic antidepressants.^[^ ^3^, ^15^ ^]^ Xylazine is a nonselective α‐2 adrenoceptor (α2‐AR) agonist. The drug activates both central and peripheral α2‐ARs, resulting in muscle relaxation, reduced pain response and depressed respiratory drive. Its bioavailability levels are good with oral ingestion, nasal insufflation, rectal insertion and ocular exposure. Conversely, xylazine parenteral intake is associated with higher bioavailability and hence blood levels.^[^ ^15^ ^]^ Although pharmacokinetic studies in humans are lacking, animal studies indicate that xylazine has a short distribution and elimination half‐life (<1 h).^[^ ^16^, ^17^ ^]^ Most xylazine is excreted unchanged in urine and its primary metabolite is 2,6‐dimethylaniline (DMA). Due to its elevated lipophilicity, xylazine can rapidly cross the blood–brain barrier.^[^ ^15^ ^]^

Despite its growing levels of misuse, the precise molecular mechanisms underlying xylazine‐related psychoactive effects remain unclear. Unlike conventional psychoactive agents, the pharmacological profile of xylazine includes interactions with a range of central nervous system receptors, including both the serotonin 5‐HT_7_ receptors (5‐HT_7_R) and KOR. Indeed, Bedard et al. demonstrated that xylazine inhibits radioligand binding by 50% or more at α2‐ARs, as well as 5‐HT_7_R, KOR, sigma 1 (σ1R) and sigma 2 receptors (σ2R).^[^ ^14^ ^]^ Furthermore, they demonstrated the surprising effects of xylazine on KOR antagonism.^[^ ^14^ ^]^

5‐HT_7_Rs are the most recently identified among the family of serotonin receptors. 5‐HT_7_R is implicated in mood disorders and may be a promising target for the treatment of depression and anxiety.^[^ ^18^ ^]^ It may play a role in drug and alcohol abuse, including reward and reinforcement, as well as seeking and craving behavior.^[^ ^18^, ^19^ ^]^ 5‐HT_7_Rs' role in health and disease, particularly as mediators and druggable targets for neurodegenerative diseases, is incompletely understood.^[^ ^20^ ^]^ Furthermore, 5‐HT_7_Rs have emerged as key players in stress‐related disorders, particularly depression.^[^ ^21^, ^22^ ^]^ Indeed, the functional suppression of 5‐HT_7_R is likely related to a rapid‐acting antidepressant‐like action.^[^ ^23^ ^]^ 5‐HT_7_Rs may also constitute a target for the treatment of schizophrenia.^[^ ^24^ ^]^ 5‐HT_7_Rs may also have a role in autism spectrum disorder,^[^ ^25^, ^26^ ^]^ learning/memory, circadian rhythm regulation and reward‐guided behavior.^[^ ^27^ ^]^

KORs modulate a range of physiological processes, including stress, mood, reward, pain, inflammation and remyelination.^[^ ^28^ ^]^ However, clinical use of KOR agonists is limited by adverse effects such as dysphoria, aversion, and sedation.^[^ ^28^ ^]^ Considering the role of KOR in xylazine activities, Romero et al. found that the opioid receptor antagonist naloxone and the μ‐opioid receptor (MOR) antagonist clocinnamox, but not the δ‐opioid receptor (DOR) antagonist naltrindole and the KOR antagonist nor‐binaltorphimine, antagonized the xylazine‐induced central antinociception.^[^ ^29^ ^]^ Hence, the authors suggested that there may be a significant involvement of endogenous opioids and MOR in xylazine‐induced central antinociception. In contrast, DOR and KOR may not be associated with this effect. In this respect, Goodchild et al. found that the α2‐ARs in the spinal cord appeared to be involved in the antinociception activities associated with intrathecal fentanyl,^[^ ^30^ ^]^ and this synergistic interaction with fentanyl was confirmed, in another preclinical model, by Meert et al.^[^ ^31^ ^]^ Finally, apart from its involvement in 5‐HT and opioid receptors, Choon et al. suggested that xylazine is also reported to have acted at cholinergic, H2‐histamine and dopaminergic receptors.^[^ ^13^ ^]^

This study aimed to investigate xylazine binding characteristics at the molecular level, focusing on its interactions with 5‐HT_7_R and KOR. By employing docking simulations and molecular dynamics (MD) calculations, we aimed to identify key binding residues and conformational states relevant to its pharmacological action. Furthermore, it was hypothesized that isosteric replacement (e.g., a technique used in medicinal chemistry to modify a biologically active molecule by replacing an atom or a group of atoms with another that has similar properties) could enhance xylazine's binding affinity, potentially informing the design of either safer or more harmful analogues.

## 2 RESULTS

**Figure 1:**
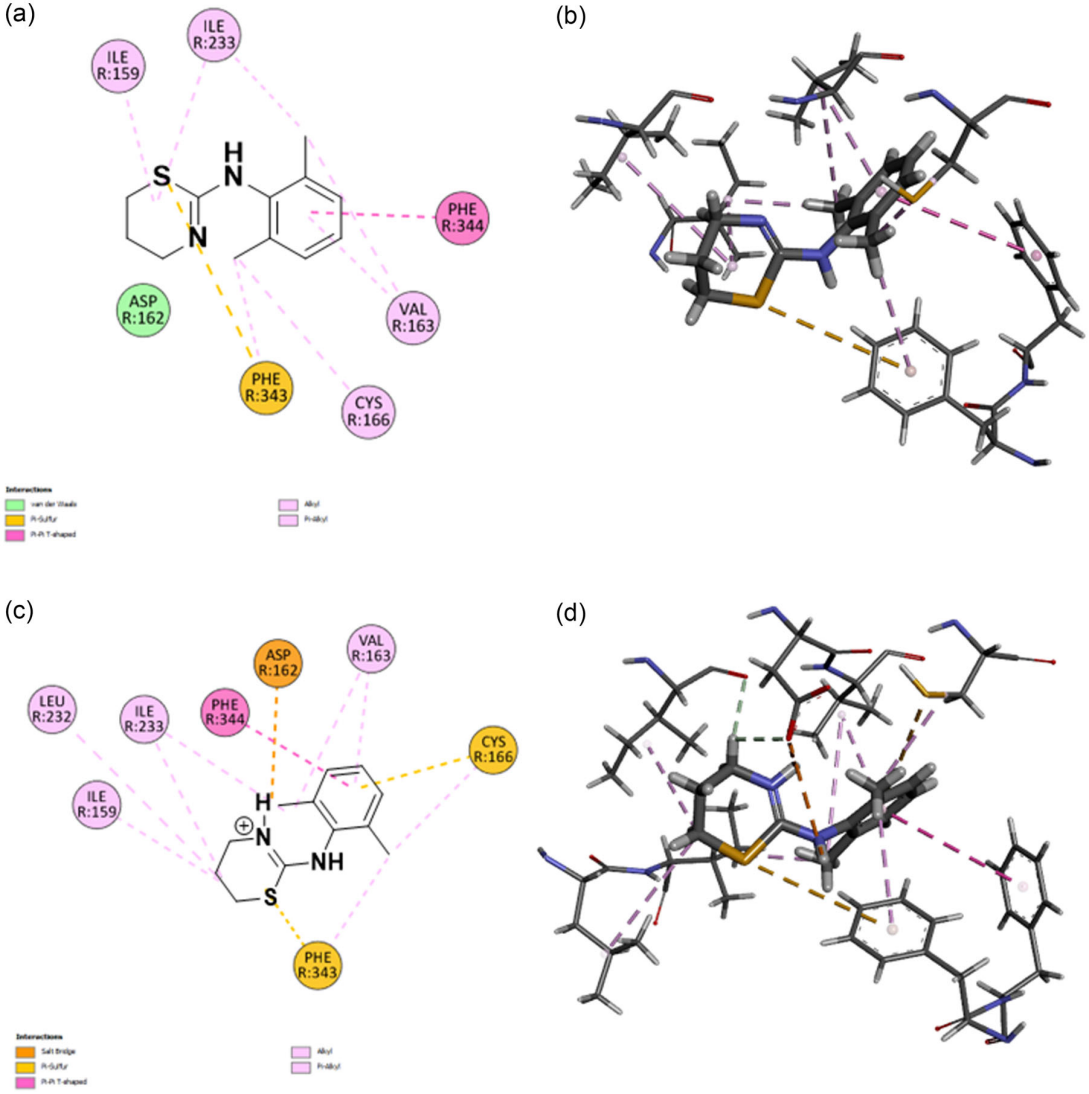
Two‐dimensional (2D; a, c) and three‐dimensional (3D; b, d) docking poses and interactions of xylazine (a, b) and xylazine‐H (c, d) in the 5‐HT_7_ serotonin receptor binding site. Pink lines indicate alkyl and π–alkyl interactions; magenta lines indicate π–π T‐shaped interactions; yellow lines indicate π–sulfur interactions; orange lines indicate a salt bridge; green residues indicate Van der Waals interactions. Following residues with Ballesteros‐Weinstein's nomenclature: Ile^3.29^ (Ile159), Asp^3.32^ (Asp162), Val^3.33^ (Val163), Cys^3.36^ (Cys166), Leu^5.27^ (Leu232), Ile^5.28^ (Ile233), Phe^6.51^ (Phe343), and Phe^6.52^ (Phe344).

In silico studies were performed using AutoDock Vina implemented in YASARA software. Using Marvin software, a study of the protonation stage of xylazine at pH 7.4 was performed. As previously reported in the literature, the compound exhibits equilibrium with its protonated state on imine nitrogen.^[^ ^32^, ^33^ ^]^ Thus, docking studies were performed within the 5‐HT_7_R pocket using both structures (PDB: 7XTC). From Figure 1, in which 2D and 3D poses of xylazine (Figure 1a,b) and protonated xylazine (Figure 1c,d) are shown, it can be inferred how the protonation state affects the interaction of the ligand with the amino acid residues in the pocket (superscripts refer to Ballesteros‐Weinstein's nomenclature).^[^ ^34^ ^]^ Xylazine establishes a π–sulfur interaction with residue Phe^6.51^ (Phe343), to which are added numerous hydrophobic, alkyl and Van der Waals interactions with residues Ile^3.29^ (Ile159), Ile^5.28^ (Ile233), Phe^6.52^ (Phe344), Val^3.33^ (Val163), Cys^3.36^ (Cys166), and Asp^3.32^ (Asp162) (Figure 1a,b). Xylazine‐H, due to the presence of the proton on the imine nitrogen, establishes a salt bridge with residue Asp^3.32^ (Asp162); in addition, the conformational change due to protonation allows the ligand to interact with residues Cys166 and Phe^6.51^ (Phe343) with π–sulfur interaction (Figure 1c,d). In addition to these, there are other interactions of a hydrophobic nature with residues Leu^5.27^ (Leu232), Ile^3.29^ (Ile159), Ile^5.28^ (Ile233), Val^3.33^ (Val163), and Phe^6.52^ (Phe344). Despite a higher interaction number, xylazine‐H has a lower Δ*G* value compared to xylazine and consequently also a lower binding energy, which are, respectively, 6.80 and 6.92.

**Figure 2:**
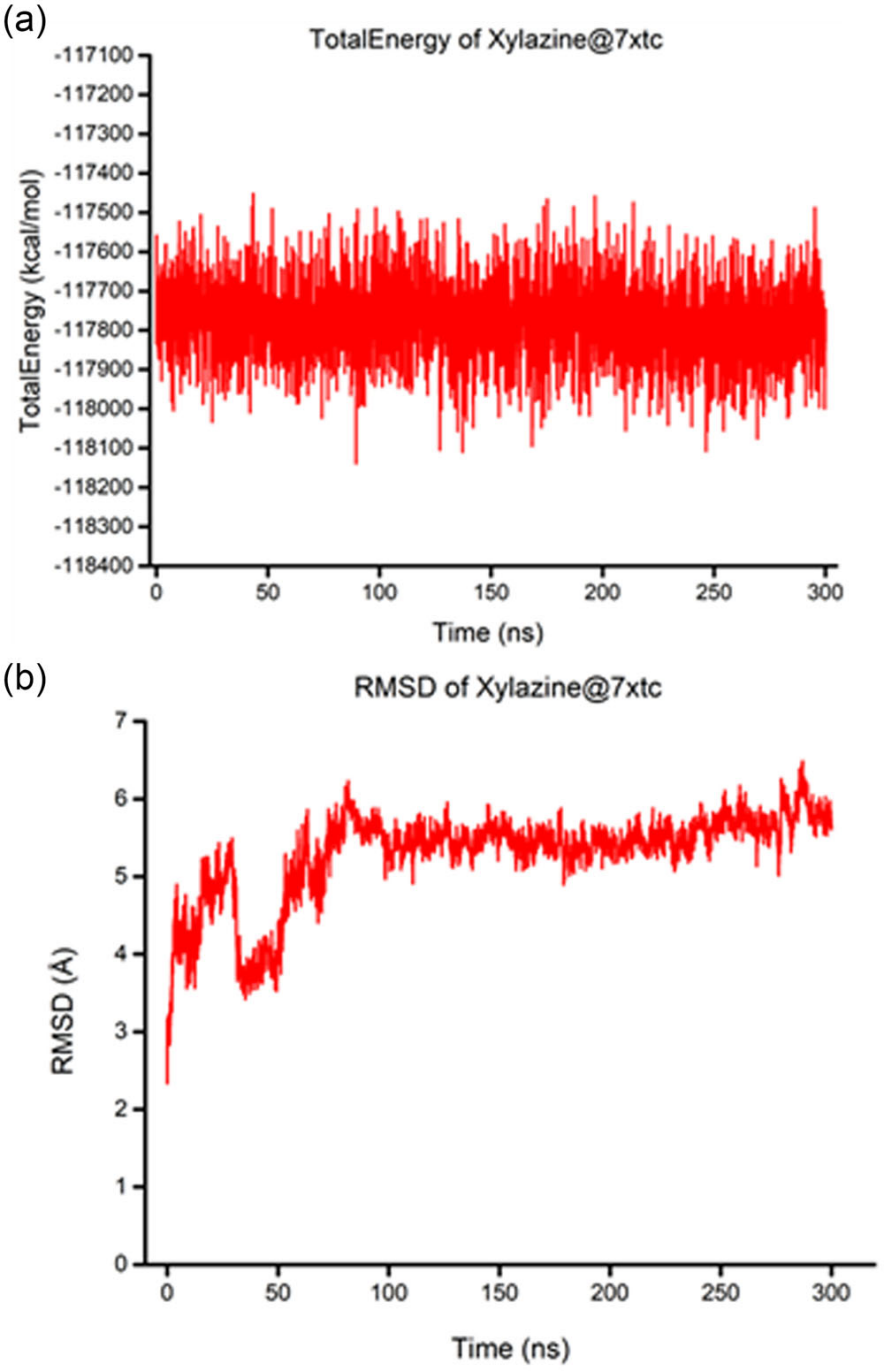
Time course of total energy (a) and root mean square deviation (RMSD) (b) for the xylazine@7xtc complex over a 300 ns MD simulation.

To further study the xylazine@7xtc complex, an MD simulation of 300 ns was conducted. Analysis of the graph of the total energy of xylazine@7xtc reveals that the complex immediately reaches equilibrium, remaining stable throughout the duration of the dynamics (Figure 2a).^[^ ^35^, ^36^, ^37^ ^]^ This is confirmed by the root mean square deviation (RMSD) plot of the complex (Figure 2b) in which one notean initial fluctuation up to 50 ns and then it remains stable until the end of the dynamics, demonstrating that the molecule remains consistently within the binding site throughout the duration of the simulation. This stability is attributed to the strong interaction with sulfur and in addition to the dense network of hydrophobic interactions that anchor the ligand within the binding site.

**Figure 3:**
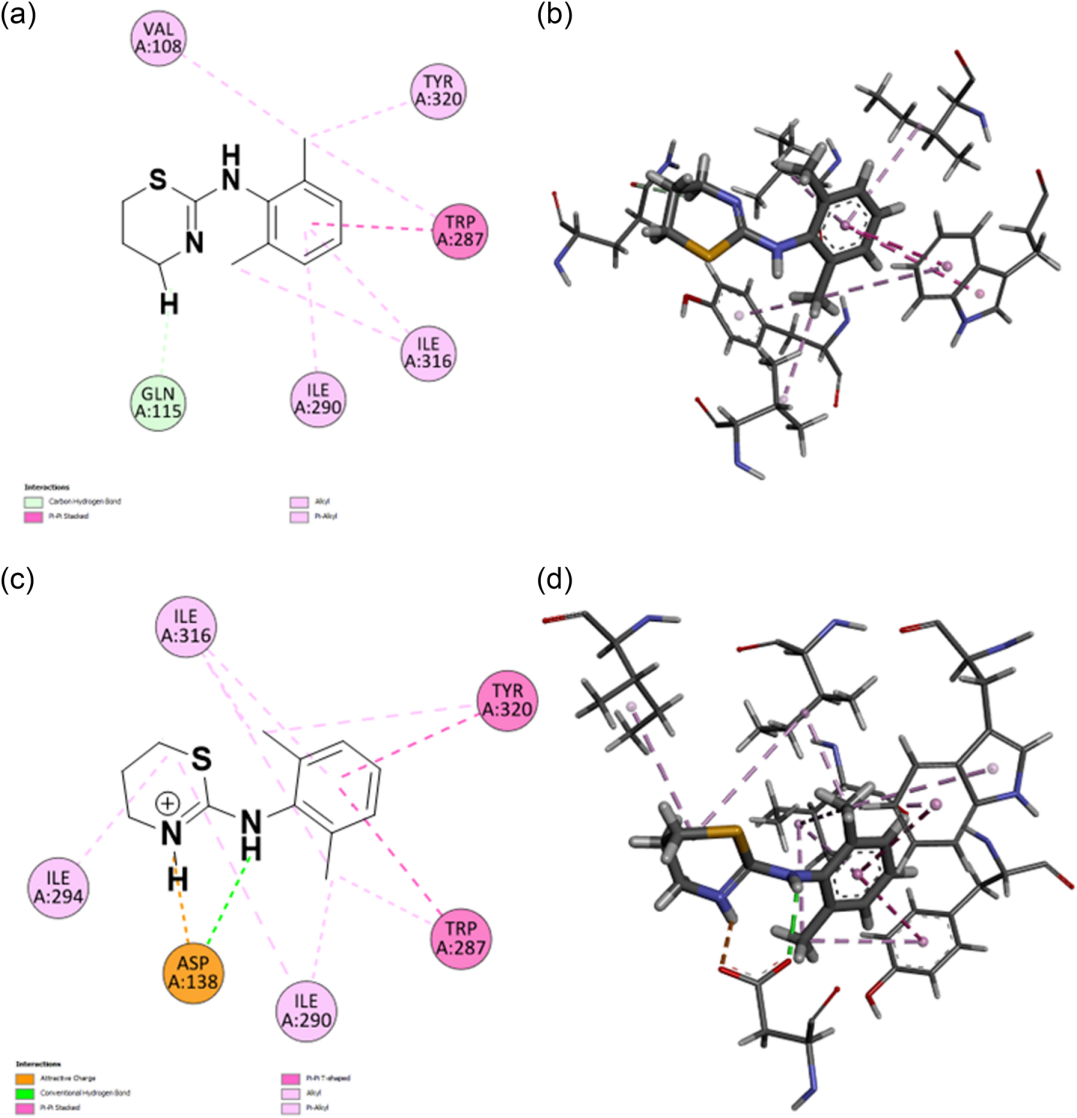
2D and 3D poses of xylazine (a, b) and xylazine‐H (c, d) inside the κ‐opioid receptor. Two‐dimensional (2D; a, c) and three‐dimensional (3D; b, d) docking poses and interactions of xylazine (a, b) and xylazine‐H (c, d) in the kappa‐opioid binding site. Pink lines indicate alky and π–alkyl interactions; magenta lines indicate π–π T‐shaped interactions; yellow lines indicate π–sulfur interactions; orange lines indicate a salt bridge; green residues indicate Van der Waals interactions. Following residues with Ballesteros‐Weinstein's nomenclature: Val^2.53^ (Val108), Gln^2.60^ (Gln115), Asp^3.32^ (Asp138), Trp^6.48^ (Trp287), Ile^6.51^ (Ile290), Ile^6.55^ (Ile294), Ile^7.39^ (Ile316), and Tyr^7.43^ (Tyr320).

Both the protonated and unprotonated forms of xylazine were also employed for docking studies with human KOR (PDB: 4DJH), with the protonation state affecting the ligand interaction with the amino acid residues of the binding pocket (superscripts refer to Ballesteros‐Weinstein's nomenclature)^[^ ^38^ ^]^ (Figure 3). Xylazine forms several hydrophobic and alkyl interactions with residues Val^2.53^ (Val108), Tyr^7.43^ (Tyr320), Trp^6.48^ (Trp287), Ile^7.39^ (Ile316), and Ile^6.51^ (Ile290), complemented by a single hydrogen bond with Gln^2.60^ (Gln115) (Figure 3a,b). The conformational change observed with xylazine‐H results in a binding mode characterized by both electrostatic and aromatic interactions within the active site of the receptor. The positively charged portion of the ligand forms a salt bridge with Asp^3.32^ (Asp138). Xylazine‐H also engages in multiple aromatic and hydrophobic interactions with residues Ile^7.39^ (Ile316), Tyr^7.43^ (Tyr320), Trp^6.48^ (Trp287), Ile^6.51^ (Ile290), and Ile^6.55^ (Ile294) of the binding site. In analogy with what was observed with the 5‐HT_7_R, xylazine‐H shows a lower binding energy compared to xylazine (6.49 and 7.10 kcal/mol, respectively).

**Figure 4:**
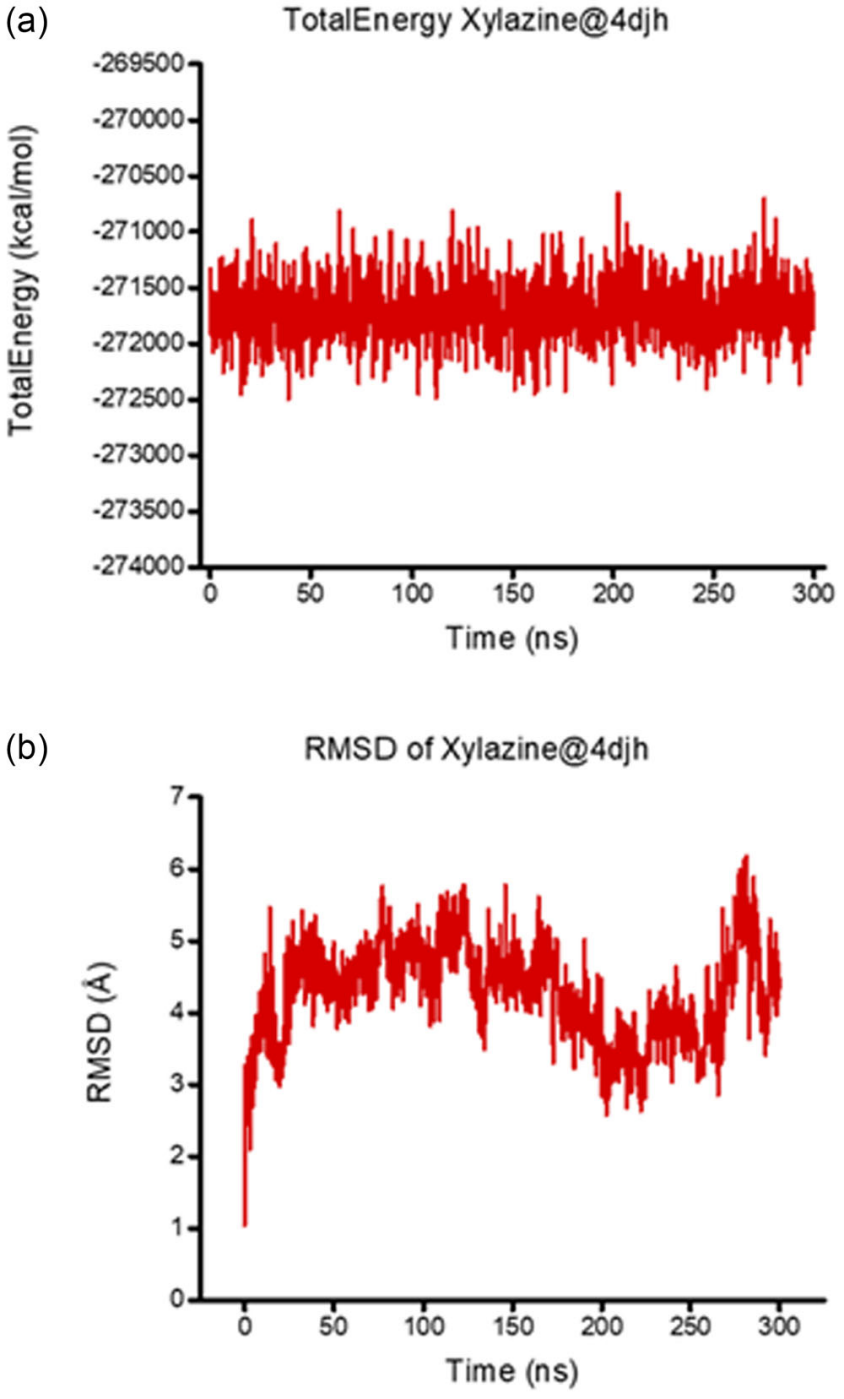
Time course of total energy (a) and root mean square deviation (RMSD) (b) for the xylazine@4djh complex over a 300 ns MD simulation.

The interaction between xylazine and the KOR was further characterized by employing a 300 ns MD simulation. Analysis of the total potential energy of the ligand–receptor complex (Figure 4a) shows that the system quickly reaches equilibrium and remains stable throughout the simulation. The complex stability is further supported by the RMSD analysis of the ligand movement after superimposing it on the receptor (Figure 4b). Following an initial stabilization phase, the ligand remains confined within the KOR binding pocket, exhibiting modest fluctuations that return to baseline levels during the simulation (Figure 4b). This stability is attributed to a network of hydrophobic interactions that anchor the ligand within the binding site. Notably, the KOR complex exhibits larger fluctuations compared to the 5‐HT_7_R complex. One can speculate that the absence of the π–sulfur interaction allows the ligand conformational flexibility to explore more favorable binding poses.

**Figure 5:**
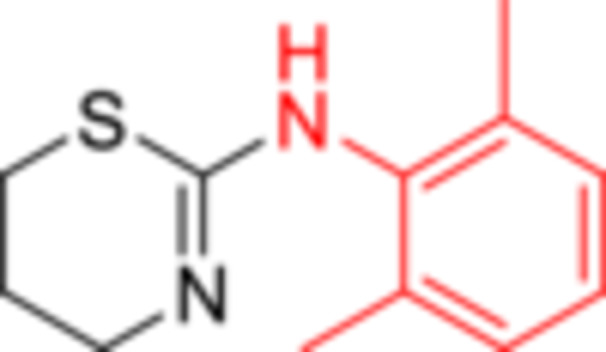
Selected portion of the xylazine structure for the scaffold‐hopping approach.

To enlarge the chemical landscape evaluation of xylazine‐like compounds, a bioisosteric and fragment replacement software tool (Spark; Cresset group) was adopted to produce a scaffold‐hopping analysis and to generate a virtual library of novel 5‐HT_7_R and KOR ligands,^[^ ^39^, ^40^ ^]^ investigating the aromatic substituent in the structure as the group mostly involved in the binding interactions. In particular, the molecule was analyzed as highlighted in Figure 5, and 500 new virtual molecules were generated (Tables S1 and S2, see Supporting information). Subsequently, each ligand was evaluated by docking calculation and the best‐performing compounds accordingly with their calculated ΔG of binding are reported in Table 1 for each receptor. For each case, the replacement was performed using the same data set of fragments previously reported.^[^ ^41^ ^]^ The results outline that the replacement generated new structures with optimized chemical features for binding both the 5‐HT_7_R and the KOR. The results of all the series indicate that the chemical landscape for this class of compounds is still huge and that small modifications may further increase the activity of the parent molecule. It is interesting to notice that some of the most powerful compounds are shared by the two analyzed receptors, that is, compounds SH1, SH2 and SH4.

**Table 1:** Selected molecules resulted from the scaffold‐hopping approach and calculated binding energies.

5‐HT_7_R	KOR
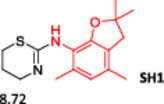	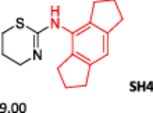
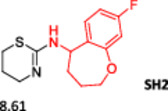	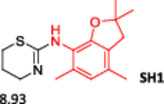
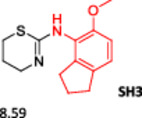	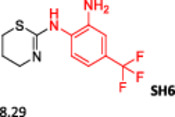
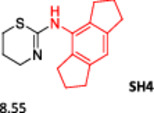	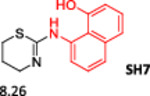
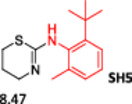	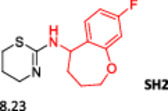

## 3 DISCUSSION AND CONCLUSIONS

To the best of the authors' knowledge, this is the first work highlighting the precise mechanism of interaction between xylazine and both the 5‐HT_7_R and the KOR, an interaction that was very recently experimentally confirmed.^[^ ^14^ ^]^ The interaction between xylazine and both receptors was considered as being very strong, although with the current approach, it cannot be concluded if xylazine is acting either as an agonist or an antagonist at any of these receptors. Furthermore, current data may be of help in future studies aiming at designing novel xylazine analogues.

Recent experimental evidence has addressed these concerns. Bedard et al.^[^ ^14^ ^]^ conducted a comprehensive pharmacodynamic characterization of xylazine. Their work demonstrated that the molecule acts as a full agonist at KOR with a submicromolar binding affinity (*K*_i_ = 0.47 μM). The study also showed that xylazine engages G‐protein signaling pathways at KOR, leading to pharmacodynamic effects that are reversed by opioid antagonists like naloxone. These findings are critical, as they establish that xylazine's effects are not solely mediated via α2‐adrenergic receptors but also involve direct opioid receptor interactions.

In addition, Bedard et al.^[^ ^14^ ^]^ provided radioligand binding data showing that xylazine also binds 5‐HT_7_R, KOR, σ1R and σ2R with significant affinity. Functional assays confirmed xylazine's ability to activate KOR and α2‐AR, supporting its role in opioid‐related withdrawal and analgesia. Notably, their study also observed sex‐specific differences in xylazine's pharmacodynamic effects, with female mice exhibiting heightened sensitivity to withdrawal symptoms.^[^ ^14^ ^]^

Taken together, the authors' computational data provides further support and extends the existing findings by providing structural insights into receptor–ligand interactions. These results also suggest that minor structural modifications through isosteric replacement may further enhance xylazine's receptor binding, potentially informing the design of both safer therapeutic analogues and strategies for mitigating its misuse. Future work should focus on in vitro functional assays to confirm whether xylazine's interactions with 5‐HT_7_R translate to functional agonist or antagonist activity. Moreover, in vivo studies on xylazine's behavioral effects in the presence of KOR antagonists could further elucidate its role in opioid withdrawal and analgesia.

Overall, these findings contribute to the growing body of literature on xylazine's emerging role as a new psychoactive substance. By bridging computational and experimental data, our study underscores the importance of multidisciplinary approaches in characterizing the pharmacology of novel substances.

In a recent study carried out on adult substance abusers, out of those with heroin or fentanyl addiction, some 8.3% reportedrecent xylazine consumption, and those who reported xylazine use also reported a median of two previous nonfatal overdoses from any drug compared with a median of one overdose among those who did not report xylazine use. Misuse of xylazine together with fentanyl and other opioids/depressants may prolong and enhance the related psychoactive effects^[^ ^42^ ^]^; furthermore, xylazine may be used for its sedative properties in speedball (e.g., “uppers” and “downers” being combined together) preparations among poly‐substance misusers.^[^ ^43^ ^]^ A recent preclinical study has shown that xylazine can increase dopamine levels in the mice reward pathways' system (e.g., in the nucleus accumbens; NAc) and that this is additive when combined with fentanyl, hence likely to enhance its effect.^[^ ^44^ ^]^ Some what consistent with this, 5‐HT_7_R as well may be involved in drug consumption‐related reward, reinforcement, and craving.^[^ ^19^ ^]^

Overall, the insights gained from the authors' study on the molecular interactions of xylazine with 5‐HT_7_R and KOR may provide a foundational understanding of its receptor binding mechanisms. Using computational approaches, the impact of structural modifications, such as isosteric replacements, was demonstrated on enhancing the binding affinity of xylazine derivatives. The generation of a virtual library of novel compounds further illustrates the chemical flexibility of this scaffold, emphasizing its potential for optimization. These findings highlight the utility of virtually generated molecules in the rational design of receptor‐specific ligands, paving the way for the development of safer and more effective therapeutic agents. By identifying structural modifications that enhance receptor binding, this work is likely to contribute not only to a better understanding of the xylazine pharmacological profile as an emerging NPS but also to the broader field of drug design.

Overall, these findings not only advance understanding of xylazine's receptor interactions but also underscore the potential of computational approaches in assessing the risk profile of emerging NPS.

Future research should focus on validating these computational predictions through experimental studies and exploring the therapeutic implications of these novel derivatives, particularly in modulating receptor pathways implicated in neuropsychiatric and analgesic processes. This approach underscores the transformative role of computational methods in modern drug discovery, offering innovative strategies to tackle challenges posed by emerging psychoactive substances.

## 4 METHODOLOGY

### 4.1 Molecular modeling

Marvin Sketch was used to create the 2D chemical structures, and the same software's MMFF94 force field was used to apply molecular mechanics energy minimization to each structure.^[^ ^45^ ^]^ The 3D geometry of all compounds was then optimized using PM3 Hamiltonian,^[^ ^46^ ^]^ as implemented in the MOPAC 2016 package assuming a pH of 7.0. Once built and optimized, all structures were used in the bio isostere replacement tool Spark 10.7.0.^[^ ^47^ ^]^ The replacement/growing was performed using the same 178,558 fragments for each part; in particular, the fragments derived from ChEMBL and Zinc databases with a protocol already reported and validated.^[^ ^48^, ^49^, ^50^, ^51^, ^52^, ^53^, ^54^ ^]^ Using AutoDock's default docking parameters and a validated protocol, docking calculations were performed.^[^ ^50^, ^55^, ^56^ ^]^ The setup was done with YASARA.^[^ ^57^ ^]^ PDB id: 7XTC for 5‐HT_7_R and 4DJH for KOR were downloaded from the Protein Data Bank (www.rcsb.org) and used for the calculations. The MD simulations of the complexes were performed with the YASARA suite. A periodic simulation cell extending 10 Å from the surface of the protein was employed. A 15 Å cuboid cell was instead employed for the KOR. The cell was filled with water, with a maximum sum of all water bumps of 1.0 Å and a density of 0.997 g/mL. The setup included optimizing the hydrogen bonding network^[^ ^58^ ^]^ to increase the solute stability and a p*K*_a_ prediction to fine‐tune the protonation states of protein residues at the chosen pH of 7.4.^[^ ^59^ ^]^ With an excess of either Na or Cl to neutralize the cell, NaCl ions were supplied at a physiological concentration of 0.9%. The simulation was run using the ff14SB force field^[^ ^60^ ^]^ for the solute, GAFF2,^[^ ^61^ ^]^ AM1BCC^[^ ^62^ ^]^ for ligands and TIP3P for water. The cutoff was 10 Å for Van der Waals forces (the default used by AMBER),^[^ ^63^ ^]^ and no cutoff was applied to electrostatic forces (using the Particle Mesh Ewald algorithm).^[^ ^64^ ^]^ The equations of motions were integrated with multiple time steps of 2.5 fs for bonded interactions and 5.0 fs for nonbonded interactions at a temperature of 298 K and a pressure of 1 atm using algorithms described in detail previously.^[^ ^65^, ^66^ ^]^ A short MD simulation was run on the solvent only to remove clashes. The entire system was then energy minimized using a steepest descent minimization to remove conformational stress, followed by a simulated annealing minimization until convergence (<0.01 kcal/mol Å). Finally, a 300 ns MD simulation without any restrictions was conducted, and the conformations were recorded every 250 ps.

The MD trajectory, including the total potential energy of the system and the ligand movement RMSD, was calculated with the YASARA *md_analyze* function.

## CONFLICTS OF INTEREST STATEMENT

Fabrizio Schifano formally advises the European Drugs Agency (EUDA) NPS working group. The remaining authors declare no conflict of interest.

## Supporting information

Supporting information.

## Data Availability

The data that support the findings of this study are available on request from the corresponding author. The data are not publicly available due to privacy or ethical restrictions.
