# Supplementary material for: Xylazine as an emerging new psychoactive substance; focuses on both 5‐HT7 and κ‐opioid receptors' molecular interactions and isosteric replacement
Source: Arch Pharm (Weinheim). 2025 Mar 17;358(3):e2500041. doi: 10.1002/ardp.202500041 (PMC11911908; doi:10.1002/ardp.202500041)
Supplement: Supplementary file 1 — Supporting information. [file ARDP-358-e2500041-s001.docx]

**Xylazine as an emerging new psychoactive substance; focus on both 5-HT_7_ and κ-opioid receptors’ molecular interactions and isosteric replacement.**

Giuseppe Floresta ^1,2^, Alberto Granzotto ^3,4^, Vincenzo Patamia ^2^, Davide Arillotta^1,5^, Gabriele Duccio Papanti ^1,6^, Amira Guirguis ^7^, John M. Corkery ^1^, Giovanni Martinotti ^1,4^, Stefano L. Sensi ^3,4,8,9^, Fabrizio Schifano ^1^

^1^ Psychopharmacology, Drug Misuse and Novel Psychoactive Substances Research Unit, School of Life and Medical Sciences, University of Hertfordshire, Hatfield, United Kingdom

^2^ Department of Drug and Health Sciences, University of Catania, Catania, Italy

^3^ Center for Advanced Studies and Technology – CAST, University G. d’Annunzio of Chieti-Pescara, Chieti, Italy

^4^ Department of Neuroscience, Imaging, and Clinical Sciences, University G. d’Annunzio of Chieti-Pescara, Chieti, Italy

^5^ Department of Neurosciences, Psychology, Drug Research, and Child Health, Section of Pharmacology and Toxicology, University of Florence, Florence, Italy

^6^ Tolmezzo Community Mental Health Centre, ASUFC Mental Health and Addiction Department, Tolmezzo, Italy

^7^ Swansea University Medical School, Swansea University, Swansea, United Kingdom

^8^ Institute for Advanced Biomedical Technologies – ITAB, University G. d’Annunzio of Chieti-Pescara, Chieti, Italy

^9^ Institute of Neurology, SS Annunziata University Hospital, University G. d’Annunzio of Chieti-Pescara, Chieti, Italy

Table S1: SMILES, Bind.energy and Contacting receptor residues for the 500 molecules resulted from the scaffold-hopping approach for 5-HT_7_R.

| SMILES | Bind.energy[kcal/mol] | Contacting receptor residues |
| --- | --- | --- |
| N1=C(SCCC1)Nc1c2OC(Cc2c(cc1C)C)(C)C | 8.716 | R PHE 158 R ILE 159 R ASP 162 R VAL 163 R CYS 166 R THR 167 R ILE 213 R LEU 232 R ILE 233 R SER 234 R TYR 239 R THR 240 R SER 243 R THR 244 R ALA 247 R PHE 343 R PHE 344 R SER 347 R LEU 370 |
| N1=C(SCCC1)N[C@@H]1CCCOc2c1ccc(F)c2 | 8.605 | R PHE 158 R ILE 159 R ASP 162 R VAL 163 R CYS 166 R THR 167 R ILE 213 R LEU 232 R ILE 233 R TYR 239 R THR 240 R SER 243 R THR 244 R ALA 247 R TRP 340 R PHE 343 R PHE 344 R SER 347 R LEU 370 |
| N1=C(SCCC1)Nc1c2CCCc2ccc1OC | 8.587 | R ASP 162 R VAL 163 R CYS 166 R THR 167 R ILE 213 R LEU 232 R ILE 233 R TYR 239 R THR 240 R SER 243 R THR 244 R ALA 247 R TRP 340 R PHE 343 R PHE 344 R SER 347 R LEU 370 |
| N1=C(SCCC1)Nc1c2CCCc2cc2CCCc12 | 8.551 | R ASP 162 R VAL 163 R CYS 166 R THR 167 R ILE 213 R LEU 232 R ILE 233 R SER 234 R GLN 235 R TYR 239 R THR 240 R SER 243 R THR 244 R ALA 247 R PHE 343 R PHE 344 R SER 347 R ARG 350 R LEU 370 |
| N1=C(SCCC1)Nc1c(C(C)(C)C)cccc1C | 8.473 | R PHE 158 R ILE 159 R ASP 162 R VAL 163 R CYS 166 R THR 167 R ILE 213 R LEU 232 R ILE 233 R TYR 239 R THR 240 R SER 243 R THR 244 R ALA 247 R TRP 340 R PHE 343 R PHE 344 R SER 347 R LEU 370 |
| N1=C(SCCC1)Nc1c2c(ccc1O)cccc2 | 8.457 | R PHE 158 R ILE 159 R ASP 162 R VAL 163 R CYS 166 R THR 167 R ILE 213 R LEU 232 R ILE 233 R THR 240 R SER 243 R THR 244 R ALA 247 R PHE 343 R PHE 344 R SER 347 |
| N1=C(SCCC1)Nc1c2CCCCc2ccc1 | 8.337 | R PHE 158 R ILE 159 R ASP 162 R VAL 163 R CYS 166 R THR 167 R ILE 213 R LEU 232 R ILE 233 R THR 240 R SER 243 R THR 244 R ALA 247 R TRP 340 R PHE 343 R PHE 344 R SER 347 R LEU 370 |
| N1=C(SCCC1)Nc1c(c(F)cc(c1)C(=O)N)C | 8.325 | R PHE 158 R ILE 159 R ASP 162 R VAL 163 R CYS 166 R LEU 232 R ILE 233 R THR 240 R SER 243 R THR 244 R ALA 247 R PHE 343 R PHE 344 R SER 347 R LEU 370 |
| N1=C(SCCC1)Nc1c2c(CC[C@H](O)C2)ccc1 | 8.289 | R ASP 162 R VAL 163 R CYS 166 R THR 167 R ILE 213 R LEU 232 R ILE 233 R THR 240 R SER 243 R THR 244 R ALA 247 R PHE 343 R PHE 344 R SER 347 R LEU 370 |
| N1=C(SCCC1)N[C@@H]1C(CCc2c1cccc2)(C)C | 8.282 | R ILE 159 R ASP 162 R VAL 163 R CYS 166 R THR 167 R ILE 213 R LEU 232 R ILE 233 R TYR 239 R THR 240 R SER 243 R THR 244 R ALA 247 R TRP 340 R PHE 343 R PHE 344 R SER 347 R LEU 370 |
| N1=C(SCCC1)Nc1c(ccc(c1)C(=O)NC)C | 8.254 | R PHE 158 R ILE 159 R ASP 162 R VAL 163 R CYS 166 R LEU 232 R ILE 233 R THR 240 R SER 243 R THR 244 R ALA 247 R PHE 343 R PHE 344 R SER 347 R LEU 370 |
| N1=C(SCCC1)N[C@H]1CCCOc2c1cccc2 | 8.156 | R PHE 158 R ILE 159 R ASP 162 R VAL 163 R CYS 166 R THR 167 R ILE 213 R LEU 232 R ILE 233 R TYR 239 R THR 240 R SER 243 R THR 244 R ALA 247 R PHE 343 R PHE 344 R SER 347 R LEU 370 |
| N1=C(SCCC1)Nc1c2c(ccc1)cccc2 | 8.156 | R ASP 162 R VAL 163 R CYS 166 R THR 167 R ILE 213 R LEU 232 R ILE 233 R GLN 235 R THR 240 R SER 243 R THR 244 R ALA 247 R PHE 343 R PHE 344 R SER 347 R ARG 350 |
| N1=C(SCCC1)Cc1c2c(ccc1C)cccc2 | 8.145 | R PHE 158 R ILE 159 R ASP 162 R VAL 163 R CYS 166 R THR 167 R ILE 213 R LEU 232 R ILE 233 R TYR 239 R THR 240 R SER 243 R THR 244 R ALA 247 R TRP 340 R PHE 343 R PHE 344 R SER 347 |
| N1=C(SCCC1)N[C@H]1[C@@H](CCc2c1cccc2)C | 8.123 | R PHE 158 R ILE 159 R ASP 162 R VAL 163 R CYS 166 R THR 167 R ILE 213 R LEU 232 R ILE 233 R TYR 239 R THR 240 R SER 243 R THR 244 R ALA 247 R PHE 343 R PHE 344 R SER 347 R LEU 370 |
| N1=C(SCCC1)N1CC[C@H](c2c1c(O)ccc2)C | 8.115 | R PHE 158 R ILE 159 R ASP 162 R VAL 163 R CYS 166 R THR 167 R ILE 213 R LEU 232 R ILE 233 R TYR 239 R SER 243 R ALA 247 R TRP 340 R PHE 343 R PHE 344 R SER 347 R LEU 370 |
| N1=C(SCCC1)Nc1c(CC(F)(F)F)cccc1 | 8.104 | R PHE 158 R ILE 159 R ASP 162 R VAL 163 R CYS 166 R THR 167 R ILE 213 R LEU 232 R ILE 233 R SER 243 R ALA 247 R TRP 340 R PHE 343 R PHE 344 R SER 347 R ARG 350 |
| N1=C(SCCC1)Nc1c(c(ccc1)C(F)(F)F)C | 8.072 | R PHE 158 R ILE 159 R ASP 162 R VAL 163 R CYS 166 R THR 167 R LEU 232 R ILE 233 R THR 244 R ALA 247 R TRP 340 R PHE 343 R PHE 344 R SER 347 R LEU 370 |
| N1=C(SCCC1)Nc1c2c(ccc1)cccc2O | 8.058 | R VAL 138 R PHE 158 R ILE 159 R ASP 162 R VAL 163 R CYS 166 R CYS 231 R LEU 232 R ILE 233 R SER 243 R ALA 247 R TRP 340 R PHE 343 R PHE 344 R ARG 367 R LEU 370 R TYR 374 |
| N1=C(SCCC1)N1c2c(CCC1)cccc2C | 8.048 | R ILE 159 R ASP 162 R VAL 163 R CYS 166 R THR 167 R ILE 213 R ILE 233 R THR 240 R SER 243 R THR 244 R ALA 247 R PHE 343 R PHE 344 R SER 347 R LEU 370 |
| N1=C(SCCC1)Nc1c(cc(cc1)C(F)(F)F)C | 8.036 | R PHE 158 R ILE 159 R ASP 162 R VAL 163 R CYS 166 R THR 167 R ILE 213 R LEU 232 R ILE 233 R SER 243 R THR 244 R ALA 247 R PHE 343 R PHE 344 R SER 347 R LEU 370 |
| N1=C(SCCC1)Nc1cc(C(F)(F)F)ccc1C | 8.029 | R PHE 158 R ILE 159 R ASP 162 R VAL 163 R CYS 166 R THR 167 R LEU 232 R ILE 233 R THR 240 R SER 243 R THR 244 R ALA 247 R PHE 343 R PHE 344 R SER 347 R LEU 370 |
| N1=C(SCCC1)N1CC[C@H](O)c2c1cccc2 | 8.026 | R ILE 159 R ASP 162 R VAL 163 R CYS 166 R THR 167 R ILE 213 R LEU 232 R ILE 233 R TYR 239 R THR 240 R SER 243 R THR 244 R ALA 247 R PHE 343 R PHE 344 R SER 347 R LEU 370 |
| N1=C(SCCC1)Nc1c2c(ccc1)c(=O)[nH]cc2 | 8.025 | R ASP 162 R VAL 163 R CYS 166 R THR 167 R ILE 213 R LEU 232 R ILE 233 R GLN 235 R SER 243 R THR 244 R ALA 247 R PHE 343 R PHE 344 R SER 347 R ARG 350 |
| N1=C(SCCC1)Nc1c(ccc(c1)C(=O)N)C | 8.012 | R PHE 158 R ILE 159 R ASP 162 R VAL 163 R CYS 166 R LEU 232 R ILE 233 R THR 240 R SER 243 R THR 244 R ALA 247 R PHE 343 R PHE 344 R SER 347 R LEU 370 |
| N1=C(SCCC1)Nc1c(N2CCCC2)cccc1F | 7.93 | R PHE 158 R ILE 159 R ASP 162 R VAL 163 R CYS 166 R THR 167 R ILE 213 R LEU 232 R ILE 233 R SER 234 R TYR 239 R THR 240 R SER 243 R THR 244 R ALA 247 R PHE 343 R PHE 344 R SER 347 R LEU 370 |
| N1=C(SCCC1)N1c2c(CCC1)c(ccc2)C(=O)N | 7.897 | R PHE 158 R ILE 159 R ASP 162 R VAL 163 R CYS 166 R CYS 231 R LEU 232 R ILE 233 R GLN 235 R SER 243 R PHE 343 R PHE 344 R SER 347 R ARG 350 R ARG 367 R LEU 370 |
| N1=C(SCCC1)Nc1c2c(sc1)cccc2 | 7.892 | R ILE 159 R ASP 162 R VAL 163 R CYS 166 R THR 167 R ILE 213 R LEU 232 R ILE 233 R SER 243 R THR 244 R ALA 247 R PHE 343 R PHE 344 R SER 347 R LEU 370 |
| N1=C(SCCC1)Nc1c2CCOCc2ccc1 | 7.882 | R PHE 158 R ILE 159 R ASP 162 R VAL 163 R CYS 166 R LEU 232 R ILE 233 R THR 244 R TRP 340 R PHE 343 R PHE 344 R SER 347 R LEU 370 |
| N1=C(SCCC1)Nc1c(ccc(c1)C#N)C | 7.876 | R PHE 158 R ILE 159 R ASP 162 R VAL 163 R CYS 166 R LEU 232 R ILE 233 R THR 240 R SER 243 R THR 244 R ALA 247 R PHE 343 R PHE 344 R SER 347 R LEU 370 |
| N1=C(SCCC1)Nc1c(cc2c([nH]cc2)c1)C | 7.866 | R PHE 158 R ILE 159 R ASP 162 R VAL 163 R CYS 166 R ILE 213 R LEU 232 R ILE 233 R THR 240 R SER 243 R THR 244 R ALA 247 R PHE 343 R PHE 344 R SER 347 R LEU 370 |
| N1=C(SCCC1)Nc1c(n2nccc2)cccc1F | 7.835 | R ASP 162 R VAL 163 R CYS 166 R THR 167 R ILE 213 R LEU 232 R ILE 233 R SER 234 R THR 240 R SER 243 R THR 244 R ALA 247 R PHE 343 R PHE 344 R SER 347 R LEU 370 |
| N1=C(SCCC1)N1CCCc2c1c(F)ccc2 | 7.816 | R ILE 159 R ASP 162 R VAL 163 R CYS 166 R THR 167 R ILE 213 R LEU 232 R ILE 233 R TYR 239 R THR 240 R SER 243 R THR 244 R ALA 247 R TRP 340 R PHE 343 R PHE 344 R SER 347 R LEU 370 |
| N1=C(SCCC1)Nc1cc(ccc1Cl)C(F)(F)F | 7.806 | R PHE 158 R ILE 159 R ASP 162 R VAL 163 R CYS 166 R ILE 213 R LEU 232 R ILE 233 R THR 240 R SER 243 R THR 244 R ALA 247 R PHE 343 R PHE 344 R SER 347 |
| N1=C(SCCC1)N[C@@H]1CCN(c2c1cccc2)C | 7.801 | R PHE 158 R ILE 159 R ASP 162 R VAL 163 R CYS 166 R THR 167 R ILE 213 R LEU 232 R ILE 233 R TYR 239 R SER 243 R THR 244 R ALA 247 R PHE 343 R PHE 344 R SER 347 R ARG 367 R LEU 370 |
| N1=C(SCCC1)N1CCCCc2c1cccc2 | 7.796 | R ILE 159 R ASP 162 R VAL 163 R CYS 166 R THR 167 R ILE 213 R LEU 232 R ILE 233 R THR 240 R SER 243 R THR 244 R ALA 247 R TRP 340 R PHE 343 R PHE 344 R SER 347 R LEU 370 |
| N1=C(SCCC1)Nc1c(n2ccnc2)cccc1 | 7.793 | R ILE 159 R ASP 162 R VAL 163 R CYS 166 R THR 167 R ILE 213 R LEU 232 R ILE 233 R SER 234 R TYR 239 R THR 240 R SER 243 R THR 244 R ALA 247 R PHE 343 R PHE 344 R SER 347 R LEU 370 |
| N1=C(SCCC1)Nc1c(ccc(c1)C(=O)C)C | 7.777 | R PHE 158 R ILE 159 R ASP 162 R VAL 163 R CYS 166 R LEU 232 R ILE 233 R TYR 239 R THR 240 R SER 243 R THR 244 R ALA 247 R PHE 343 R PHE 344 R SER 347 R LEU 370 |
| N1=C(SCCC1)Nc1c(C(C)C)cccc1CC | 7.722 | R PHE 158 R ILE 159 R ASP 162 R VAL 163 R CYS 166 R THR 167 R ILE 213 R LEU 232 R ILE 233 R TYR 239 R THR 240 R SER 243 R THR 244 R ALA 247 R TRP 340 R PHE 343 R PHE 344 R SER 347 R LEU 370 |
| N1=C(SCCC1)Nc1c2nsnc2ccc1Cl | 7.703 | R PHE 158 R ILE 159 R ASP 162 R VAL 163 R CYS 166 R THR 167 R ILE 213 R LEU 232 R ILE 233 R THR 240 R SER 243 R THR 244 R ALA 247 R PHE 343 R PHE 344 R SER 347 R LEU 370 |
| N1=C(SCCC1)N[C@H]1[C@@H](CCCC1)C(C)(C)C | 7.687 | R PHE 158 R ILE 159 R ASP 162 R VAL 163 R CYS 166 R THR 167 R LEU 232 R ILE 233 R SER 243 R THR 244 R ALA 247 R TRP 340 R PHE 343 R PHE 344 R SER 347 R LEU 370 |
| N1=C(SCCC1)Nc1c(cc2OCOc2c1)C | 7.679 | R PHE 158 R ILE 159 R ASP 162 R VAL 163 R CYS 166 R LEU 232 R ILE 233 R THR 240 R SER 243 R THR 244 R ALA 247 R PHE 343 R PHE 344 R SER 347 R LEU 370 |
| N1=C(SCCC1)Nc1c(c(ccc1)C(=O)N)C | 7.676 | R PHE 158 R ILE 159 R ASP 162 R VAL 163 R CYS 166 R THR 167 R ILE 213 R LEU 232 R ILE 233 R TYR 239 R THR 240 R SER 243 R THR 244 R ALA 247 R PHE 343 R PHE 344 R SER 347 R LEU 370 |
| N1=C(SCCC1)N1c2c(CCC1)cc(OC)cc2OC | 7.673 | R PHE 158 R ILE 159 R ASP 162 R VAL 163 R CYS 166 R THR 167 R ILE 170 R ILE 213 R LEU 232 R ILE 233 R TYR 239 R THR 240 R SER 243 R THR 244 R ALA 247 R PHE 248 R TRP 340 R PHE 343 R PHE 344 R SER 347 R LEU 370 R TYR 374 |
| N1=C(SCCC1)Nc1ccc(cc1F)C(F)(F)F | 7.67 | R PHE 158 R ILE 159 R ASP 162 R VAL 163 R THR 167 R ILE 213 R LEU 232 R ILE 233 R SER 243 R THR 244 R ALA 247 R PHE 343 R PHE 344 R SER 347 R ARG 367 R LEU 370 |
| N1=C(SCCC1)Nc1c(cc(N)c(Cl)c1)C | 7.64 | R PHE 158 R ILE 159 R ASP 162 R VAL 163 R CYS 166 R THR 167 R ILE 213 R LEU 232 R ILE 233 R SER 243 R ALA 247 R TRP 340 R PHE 343 R PHE 344 R SER 347 R LEU 370 |
| N1=C(SCCC1)Nc1c(c2c([nH]cc2)nc1)C | 7.632 | R PHE 158 R ILE 159 R ASP 162 R VAL 163 R CYS 166 R THR 167 R ILE 213 R LEU 232 R ILE 233 R SER 243 R THR 244 R ALA 247 R PHE 343 R PHE 344 R SER 347 |
| N1=C(SCCC1)Nc1c2OCOc2ccc1Cl | 7.627 | R VAL 163 R CYS 166 R ILE 213 R LEU 232 R ILE 233 R SER 234 R GLN 235 R THR 240 R SER 243 R THR 244 R ALA 247 R PHE 343 R PHE 344 R SER 347 R ARG 350 R LEU 370 |
| N1=C(SCCC1)Nc1c(c(ccc1)C(=O)OC)C | 7.621 | R PHE 158 R ILE 159 R ASP 162 R VAL 163 R ILE 213 R CYS 231 R LEU 232 R ILE 233 R THR 240 R SER 243 R THR 244 R ALA 247 R PHE 343 R PHE 344 R SER 347 R ARG 350 R LEU 370 |
| N1=C(SCCC1)Nc1c(C(C)C)cccc1C | 7.616 | R PHE 158 R ILE 159 R ASP 162 R VAL 163 R CYS 166 R THR 167 R ILE 213 R LEU 232 R ILE 233 R TYR 239 R THR 240 R SER 243 R THR 244 R ALA 247 R TRP 340 R PHE 343 R PHE 344 R SER 347 |
| N1=C(SCCC1)N[C@H](c1c(OC)ccc(c1)C)C | 7.562 | R PHE 158 R ILE 159 R ASP 162 R VAL 163 R CYS 166 R THR 167 R ILE 213 R LEU 232 R ILE 233 R SER 243 R THR 244 R ALA 247 R TRP 340 R PHE 343 R PHE 344 R SER 347 R LEU 370 R TYR 374 |
| N1=C(SCCC1)C[n+]1c2c(ccc1)cccc2 | 7.537 | R PHE 158 R ILE 159 R ASP 162 R VAL 163 R CYS 166 R THR 167 R ILE 213 R LEU 232 R ILE 233 R SER 243 R THR 244 R ALA 247 R TRP 340 R PHE 343 R PHE 344 R SER 347 R LEU 370 |
| N1=C(SCCC1)N[C@@H](c1cc(cc(c1)C)C)C | 7.53 | R PHE 158 R ILE 159 R ASP 162 R VAL 163 R CYS 166 R THR 167 R ILE 213 R CYS 231 R LEU 232 R ILE 233 R SER 234 R TYR 239 R THR 240 R SER 243 R THR 244 R ALA 247 R TRP 340 R PHE 343 R PHE 344 R SER 347 R LEU 370 |
| N1=C(SCCC1)Nc1c(Cl)c2c([nH]nc2)cc1 | 7.516 | R ASP 162 R VAL 163 R CYS 166 R THR 167 R ILE 213 R LEU 232 R ILE 233 R SER 243 R THR 244 R ALA 247 R PHE 343 R PHE 344 R SER 347 R ARG 367 R LEU 370 R TYR 374 |
| N1=C(SCCC1)Nc1c(CS(=O)(=O)C)cccc1 | 7.515 | R PHE 158 R ILE 159 R ASP 162 R VAL 163 R CYS 166 R THR 167 R LEU 232 R ILE 233 R TYR 239 R THR 240 R SER 243 R THR 244 R ALA 247 R PHE 343 R PHE 344 R SER 347 |
| N1=C(SCCC1)Nc1cc(C(C)(C)C)ccc1C | 7.512 | R VAL 138 R PHE 158 R ILE 159 R ASP 162 R VAL 163 R ILE 213 R CYS 231 R LEU 232 R ILE 233 R TYR 239 R THR 240 R SER 243 R THR 244 R ALA 247 R PHE 343 R PHE 344 R SER 347 R ARG 350 R ARG 367 R LEU 370 R TYR 374 |
| N1=C(SCCC1)Nc1c(cccc1Cl)C(F)(F)F | 7.51 | R ASP 162 R VAL 163 R CYS 166 R THR 167 R ILE 213 R ILE 233 R THR 240 R SER 243 R THR 244 R ALA 247 R PHE 343 R PHE 344 R SER 347 |
| N1=C(SCCC1)Nc1c(n2nccc2)cccc1 | 7.494 | R ASP 162 R VAL 163 R CYS 166 R THR 167 R ILE 213 R LEU 232 R ILE 233 R SER 234 R TYR 239 R THR 240 R SER 243 R ALA 247 R PHE 343 R PHE 344 R SER 347 R LEU 370 |
| N1=C(SCCC1)N1CCC[C@@H](c2c1cccc2)C | 7.492 | R PHE 158 R ILE 159 R ASP 162 R VAL 163 R CYS 166 R THR 167 R LEU 232 R ILE 233 R ALA 247 R TRP 340 R PHE 343 R PHE 344 R SER 347 R ARG 350 R LEU 370 |
| N1=C(SCCC1)Nc1c(c2sccc2)cccc1 | 7.489 | R ASP 162 R VAL 163 R CYS 166 R THR 167 R ILE 213 R LEU 232 R ILE 233 R SER 234 R TYR 239 R THR 240 R SER 243 R THR 244 R ALA 247 R PHE 343 R PHE 344 R SER 347 R LEU 370 R TYR 374 |
| N1=C(SCCC1)Nc1c2CCCCc2sc1 | 7.487 | R ILE 159 R ASP 162 R VAL 163 R CYS 166 R THR 167 R ILE 213 R LEU 232 R ILE 233 R SER 243 R THR 244 R ALA 247 R TRP 340 R PHE 343 R PHE 344 R SER 347 R LEU 370 |
| N1=C(SCCC1)Nc1c(F)c(F)cc(F)c1F | 7.479 | R ASP 162 R VAL 163 R CYS 166 R THR 167 R ILE 213 R LEU 232 R ILE 233 R GLN 235 R SER 243 R THR 244 R ALA 247 R PHE 343 R PHE 344 R SER 347 R ARG 350 R LEU 370 |
| N1=C(SCCC1)Nc1c(CC)cccc1CC | 7.463 | R PHE 158 R ILE 159 R ASP 162 R VAL 163 R CYS 166 R THR 167 R ILE 213 R LEU 232 R ILE 233 R SER 234 R TYR 239 R THR 240 R SER 243 R THR 244 R ALA 247 R TRP 340 R PHE 343 R PHE 344 R SER 347 |
| N1=C(SCCC1)Nc1c(c(Cl)c(cc1O)C)C | 7.46 | R PHE 158 R ASP 162 R VAL 163 R CYS 166 R THR 167 R ILE 213 R LEU 232 R ILE 233 R SER 243 R THR 244 R ALA 247 R PHE 343 R PHE 344 R SER 347 R ARG 367 R LEU 370 R TYR 374 |
| N1=C(SCCC1)N1c2c(CCC1)cccc2OC | 7.449 | R ILE 159 R ASP 162 R VAL 163 R CYS 166 R THR 167 R ILE 213 R LEU 232 R ILE 233 R TYR 239 R THR 240 R SER 243 R THR 244 R ALA 247 R TRP 340 R PHE 343 R PHE 344 R SER 347 R LEU 370 |
| N1=C(SCCC1)Nc1c(c2c([nH]cc2)cc1)C | 7.441 | R ASP 162 R VAL 163 R CYS 166 R THR 167 R ILE 213 R LEU 232 R ILE 233 R SER 243 R THR 244 R ALA 247 R PHE 343 R PHE 344 R SER 347 R ARG 367 R LEU 370 R TYR 374 |
| N1=C(SCCC1)Nc1c(N)cc(cc1)C(F)(F)F | 7.438 | R PHE 158 R ASP 162 R VAL 163 R CYS 166 R THR 167 R ILE 213 R LEU 232 R ILE 233 R SER 243 R THR 244 R ALA 247 R PHE 343 R PHE 344 R SER 347 R ARG 367 R LEU 370 R TYR 374 |
| N1=C(SCCC1)Nc1c(ccc2nsnc12)C | 7.415 | R PHE 158 R ILE 159 R ASP 162 R VAL 163 R CYS 166 R THR 167 R ILE 213 R LEU 232 R ILE 233 R TYR 239 R SER 243 R ALA 247 R TRP 340 R PHE 343 R PHE 344 R LEU 370 |
| N1=C(SCCC1)Nc1c(c(OC)c(cc1)C)C | 7.415 | R VAL 138 R PHE 158 R ILE 159 R ASP 162 R VAL 163 R CYS 166 R THR 167 R ILE 213 R CYS 231 R LEU 232 R ILE 233 R SER 243 R THR 244 R ALA 247 R TRP 340 R PHE 343 R PHE 344 R ARG 367 R LEU 370 |
| N1=C(SCCC1)N[C@@H](c1cc(ccc1)C)C | 7.405 | R PHE 158 R ILE 159 R ASP 162 R VAL 163 R CYS 166 R THR 167 R ILE 213 R LEU 232 R ILE 233 R SER 243 R THR 244 R ALA 247 R TRP 340 R PHE 343 R PHE 344 R SER 347 R LEU 370 R TYR 374 |
| N1=C(SCCC1)N1c2c(CC1)cccc2C | 7.402 | R ILE 159 R ASP 162 R VAL 163 R CYS 166 R THR 167 R ILE 213 R LEU 232 R ILE 233 R SER 243 R THR 244 R ALA 247 R TRP 340 R PHE 343 R PHE 344 R SER 347 R LEU 370 |
| N1=C(SCCC1)Nc1c(cccc1OC)C | 7.399 | R ILE 159 R ASP 162 R VAL 163 R CYS 166 R THR 167 R ILE 213 R LEU 232 R ILE 233 R TYR 239 R THR 240 R SER 243 R THR 244 R ALA 247 R PHE 343 R PHE 344 R SER 347 R LEU 370 |
| N1=C(SCCC1)N1c2c(CN(CC1)C)cccc2 | 7.39 | R ILE 159 R ASP 162 R VAL 163 R CYS 166 R LEU 232 R ILE 233 R GLN 235 R THR 240 R SER 243 R THR 244 R ALA 247 R TRP 340 R PHE 343 R PHE 344 R SER 347 R ARG 350 R LEU 370 |
| N1=C(SCCC1)Nc1c(cc(cc1C)C#N)C | 7.388 | R VAL 138 R ASP 142 R PHE 158 R ILE 159 R ASP 162 R VAL 163 R CYS 166 R LEU 232 R ILE 233 R SER 243 R PHE 343 R PHE 344 R SER 347 R ARG 350 R ARG 367 R LEU 370 |
| N1=C(SCCC1)Nc1c(cc(cc1C)C)C | 7.384 | R PHE 158 R ILE 159 R ASP 162 R VAL 163 R CYS 166 R THR 167 R ILE 213 R LEU 232 R ILE 233 R THR 240 R SER 243 R THR 244 R ALA 247 R PHE 343 R PHE 344 R SER 347 R LEU 370 |
| N1=C(SCCC1)Nc1c(cc(O)c(C(C)C)c1)C | 7.384 | R PHE 158 R ILE 159 R ASP 162 R VAL 163 R CYS 166 R ILE 213 R LEU 232 R ILE 233 R TYR 239 R THR 240 R SER 243 R THR 244 R ALA 247 R PHE 343 R PHE 344 R SER 347 R LEU 370 |
| N1=C(SCCC1)[C@H]1CNCc2c1cccc2 | 7.375 | R PHE 158 R ILE 159 R ASP 162 R VAL 163 R CYS 166 R THR 167 R ILE 213 R LEU 232 R ILE 233 R SER 243 R THR 244 R ALA 247 R PHE 343 R PHE 344 R SER 347 |
| N1=C(SCCC1)Nc1cc(C(C)C)ccc1C | 7.373 | R PHE 158 R ILE 159 R ASP 162 R VAL 163 R CYS 166 R THR 167 R ILE 213 R LEU 232 R ILE 233 R GLN 235 R SER 243 R THR 244 R ALA 247 R PHE 343 R PHE 344 R SER 347 R ARG 367 R LEU 370 |
| N1=C(SCCC1)Nc1c(CC)c(Cl)ccc1CC | 7.369 | R PHE 158 R ILE 159 R ASP 162 R VAL 163 R CYS 166 R THR 167 R ILE 213 R LEU 232 R ILE 233 R TYR 239 R THR 240 R SER 243 R THR 244 R ALA 247 R TRP 340 R PHE 343 R PHE 344 R SER 347 R LEU 370 R TYR 374 |
| N1=C(SCCC1)Nc1c(c(N)ccc1)C | 7.369 | R ILE 159 R ASP 162 R VAL 163 R CYS 166 R THR 167 R ILE 213 R LEU 232 R ILE 233 R SER 243 R THR 244 R ALA 247 R TRP 340 R PHE 343 R PHE 344 R SER 347 R LEU 370 |
| N1=C(SCCC1)Nc1c(N)ccc(c1)C(=O)N | 7.367 | R PHE 158 R ASP 162 R VAL 163 R CYS 166 R LEU 232 R ILE 233 R SER 243 R THR 244 R ALA 247 R PHE 343 R PHE 344 R SER 347 R ARG 367 R LEU 370 R TYR 374 |
| N1=C(SCCC1)Nc1c2sccc2ccc1 | 7.36 | R ILE 159 R ASP 162 R VAL 163 R CYS 166 R THR 167 R ILE 213 R LEU 232 R ILE 233 R THR 240 R SER 243 R THR 244 R ALA 247 R PHE 343 R PHE 344 R SER 347 R LEU 370 |
| N1=C(SCCC1)Nc1c([C@H](CC)C)cccc1 | 7.356 | R PHE 158 R ILE 159 R ASP 162 R VAL 163 R CYS 166 R THR 167 R ILE 213 R LEU 232 R ILE 233 R SER 234 R TYR 239 R THR 240 R SER 243 R ALA 247 R TRP 340 R PHE 343 R PHE 344 R SER 347 |
| N1=C(SCCC1)Nc1c(C(C)C)cccc1 | 7.351 | R ILE 159 R ASP 162 R VAL 163 R CYS 166 R THR 167 R ILE 213 R LEU 232 R ILE 233 R SER 243 R THR 244 R ALA 247 R TRP 340 R PHE 343 R PHE 344 R SER 347 R LEU 370 |
| N1=C(SCCC1)Nc1c(c(O)ccc1F)C | 7.344 | R ILE 159 R ASP 162 R VAL 163 R CYS 166 R THR 167 R ILE 213 R LEU 232 R ILE 233 R SER 243 R THR 244 R ALA 247 R TRP 340 R PHE 343 R PHE 344 R SER 347 R LEU 370 |
| N1=C(SCCC1)N[C@@H]1CCOc2c1cccc2 | 7.344 | R PHE 158 R ILE 159 R ASP 162 R VAL 163 R CYS 166 R ILE 213 R LEU 232 R ILE 233 R TYR 239 R SER 243 R THR 244 R ALA 247 R TRP 340 R PHE 343 R PHE 344 R SER 347 R LEU 370 |
| N1=C(SCCC1)N[C@H](c1c(F)c(F)ccc1)C | 7.325 | R PHE 158 R ILE 159 R ASP 162 R VAL 163 R CYS 166 R THR 167 R ILE 213 R LEU 232 R ILE 233 R SER 243 R THR 244 R ALA 247 R PHE 343 R PHE 344 R SER 347 R LEU 370 |
| N1=C(SCCC1)Nc1c(cc(F)cc1)C(F)(F)F | 7.316 | R ASP 162 R VAL 163 R CYS 166 R THR 167 R LEU 232 R ILE 233 R GLN 235 R SER 243 R THR 244 R ALA 247 R PHE 343 R PHE 344 R SER 347 R ARG 350 R LEU 370 |
| N1=C(SCCC1)Cc1c(cc(cc1C)C#N)C | 7.314 | R PHE 158 R ILE 159 R ASP 162 R VAL 163 R CYS 166 R THR 167 R LEU 232 R ILE 233 R SER 243 R ALA 247 R TRP 340 R PHE 343 R PHE 344 R SER 347 R LEU 370 R TYR 374 |
| N1=C(SCCC1)N[C@@H](c1c(F)cc(F)cc1)C | 7.313 | R PHE 158 R ILE 159 R ASP 162 R VAL 163 R CYS 166 R THR 167 R ILE 213 R LEU 232 R ILE 233 R SER 243 R THR 244 R ALA 247 R TRP 340 R PHE 343 R PHE 344 R SER 347 R LEU 370 |
| N1=C(SCCC1)Nc1c(cc(N)cc1)C(F)F | 7.311 | R PHE 158 R ILE 159 R ASP 162 R VAL 163 R CYS 231 R LEU 232 R ILE 233 R PHE 343 R PHE 344 R SER 347 R LEU 370 |
| N1=C(SCCC1)Nc1c(cccc1N)C(=O)N | 7.309 | R ASP 162 R VAL 163 R CYS 166 R THR 167 R ILE 213 R LEU 232 R ILE 233 R GLN 235 R SER 243 R THR 244 R ALA 247 R PHE 343 R PHE 344 R SER 347 R LEU 370 |
| N1=C(SCCC1)Nc1c(Cl)c(ccc1Cl)C | 7.302 | R PHE 158 R ILE 159 R ASP 162 R VAL 163 R CYS 166 R THR 167 R ILE 213 R LEU 232 R ILE 233 R SER 243 R THR 244 R ALA 247 R PHE 343 R PHE 344 R SER 347 |
| N1=C(SCCC1)Nc1c(NS(=O)(=O)C)cccc1 | 7.302 | R ASP 162 R VAL 163 R CYS 166 R THR 167 R ILE 213 R LEU 232 R ILE 233 R SER 234 R GLN 235 R TYR 239 R THR 240 R SER 243 R THR 244 R ALA 247 R PHE 343 R PHE 344 R SER 347 R LEU 370 |
| N1=C(SCCC1)Nc1cc(c(cc1)C)C | 7.289 | R ASP 162 R VAL 163 R THR 167 R ILE 213 R LEU 232 R ILE 233 R GLN 235 R SER 243 R THR 244 R ALA 247 R PHE 343 R PHE 344 R SER 347 R ARG 350 R LEU 370 |
| N1=C(SCCC1)Cc1c2CCOc2ccc1F | 7.274 | R PHE 158 R ILE 159 R ASP 162 R VAL 163 R CYS 166 R THR 167 R LEU 232 R ILE 233 R TYR 239 R THR 240 R SER 243 R ALA 247 R PHE 343 R PHE 344 R SER 347 R LEU 370 |
| N1=C(SCCC1)N1CCSc2c1cccc2 | 7.262 | R PHE 158 R ILE 159 R ASP 162 R VAL 163 R CYS 166 R THR 167 R LEU 232 R ILE 233 R ALA 247 R PHE 343 R PHE 344 R SER 347 |
| N1=C(SCCC1)Nc1c(CN(C)C)cc(cc1)C | 7.259 | R PHE 158 R ILE 159 R ASP 162 R VAL 163 R CYS 231 R LEU 232 R ILE 233 R GLN 235 R THR 244 R PHE 343 R PHE 344 R SER 347 R ARG 350 R LEU 370 |
| N1=C(SCCC1)Nc1c(N)c(F)cc(F)c1 | 7.24 | R PHE 158 R ILE 159 R ASP 162 R VAL 163 R CYS 166 R THR 167 R LEU 232 R ILE 233 R THR 244 R ALA 247 R PHE 343 R PHE 344 R SER 347 R LEU 370 |
| N1=C(SCCC1)Nc1c(c(O)c(cc1)C)C | 7.234 | R PHE 158 R ILE 159 R ASP 162 R VAL 163 R CYS 166 R THR 167 R ILE 213 R LEU 232 R ILE 233 R SER 243 R THR 244 R ALA 247 R PHE 343 R PHE 344 R SER 347 |
| N1=C(SCCC1)Nc1c(CC)cccc1C | 7.229 | R PHE 158 R ILE 159 R ASP 162 R VAL 163 R CYS 166 R THR 167 R ILE 213 R LEU 232 R ILE 233 R THR 240 R SER 243 R THR 244 R ALA 247 R TRP 340 R PHE 343 R PHE 344 R SER 347 R LEU 370 |
| N1=C(SCCC1)N[C@@H]1CCSc2c1cccc2 | 7.207 | R PHE 158 R ILE 159 R ASP 162 R VAL 163 R CYS 166 R THR 167 R ILE 213 R LEU 232 R ILE 233 R THR 240 R SER 243 R THR 244 R ALA 247 R TRP 340 R PHE 343 R PHE 344 R SER 347 R LEU 370 |
| N1=C(SCCC1)Nc1c(nn(c1N1CCC1)C)C | 7.203 | R ASP 162 R VAL 163 R CYS 166 R THR 167 R ILE 213 R LEU 232 R ILE 233 R SER 234 R GLN 235 R THR 240 R SER 243 R THR 244 R ALA 247 R TRP 340 R PHE 343 R PHE 344 R SER 347 R ARG 350 |
| N1=C(SCCC1)N[C@@H](c1c(F)cccc1F)C | 7.179 | R PHE 158 R ILE 159 R ASP 162 R VAL 163 R THR 167 R ILE 213 R LEU 232 R ILE 233 R SER 243 R THR 244 R ALA 247 R PHE 343 R PHE 344 R SER 347 R LEU 370 |
| N1=C(SCCC1)Nc1c(cc(F)cc1)C | 7.178 | R PHE 158 R ILE 159 R ASP 162 R VAL 163 R CYS 231 R LEU 232 R ILE 233 R PHE 343 R PHE 344 R SER 347 R LEU 370 |
| N1=C(SCCC1)Nc1c(cc(OC)c(F)c1)C | 7.176 | R PHE 158 R ILE 159 R ASP 162 R VAL 163 R CYS 166 R THR 167 R ILE 213 R LEU 232 R ILE 233 R SER 243 R THR 244 R ALA 247 R PHE 343 R PHE 344 R SER 347 R LEU 370 |
| N1=C(SCCC1)Nc1c(N)cc(cc1)C | 7.174 | R PHE 158 R ILE 159 R ASP 162 R VAL 163 R CYS 166 R CYS 231 R LEU 232 R ILE 233 R SER 243 R THR 244 R ALA 247 R TRP 340 R PHE 343 R PHE 344 R ARG 367 R LEU 370 R TYR 374 |
| N1=C(SCCC1)Nc1c(C(C)(C)C)cccc1 | 7.159 | R ASP 162 R VAL 163 R CYS 166 R THR 167 R ILE 213 R LEU 232 R ILE 233 R TYR 239 R THR 240 R SER 243 R THR 244 R ALA 247 R PHE 343 R PHE 344 R SER 347 R LEU 370 |
| N1=C(SCCC1)Nc1cc(C)ccc1C | 7.158 | R PHE 158 R ILE 159 R ASP 162 R VAL 163 R CYS 166 R THR 167 R ILE 213 R LEU 232 R ILE 233 R SER 243 R THR 244 R ALA 247 R PHE 343 R PHE 344 R SER 347 R LEU 370 |
| N1=C(SCCC1)N[C@@H]1C2(CCCC1)CCC2 | 7.158 | R PHE 158 R ILE 159 R ASP 162 R VAL 163 R CYS 166 R THR 167 R ILE 213 R LEU 232 R ILE 233 R SER 243 R THR 244 R ALA 247 R PHE 343 R PHE 344 R SER 347 |
| N1=C(SCCC1)Nc1c2c(sc1N)cccc2 | 7.155 | R ASP 162 R VAL 163 R CYS 166 R THR 167 R ILE 213 R LEU 232 R ILE 233 R SER 243 R THR 244 R ALA 247 R PHE 343 R PHE 344 R SER 347 R LEU 370 R TYR 374 |
| N1=C(SCCC1)N1CCCc2c1c(O)ccc2 | 7.154 | R PHE 158 R ILE 159 R ASP 162 R VAL 163 R CYS 231 R LEU 232 R ILE 233 R PHE 343 R SER 347 R GLU 366 R ARG 367 R LEU 370 |
| N1=C(SCCC1)N[C@@H](c1ccc(cc1)C)CC | 7.151 | R PHE 158 R ILE 159 R ASP 162 R VAL 163 R CYS 166 R LEU 232 R ILE 233 R PHE 343 R PHE 344 R SER 347 R ARG 350 R GLU 366 R LEU 370 |
| N1=C(SCCC1)NCc1c(Cl)c(Cl)ccc1 | 7.146 | R PHE 158 R ILE 159 R ASP 162 R VAL 163 R CYS 166 R THR 167 R ILE 213 R LEU 232 R ILE 233 R SER 243 R THR 244 R ALA 247 R PHE 343 R PHE 344 R SER 347 R LEU 370 |
| N1=C(SCCC1)NC(c1ccccc1)(C)C | 7.139 | R ILE 159 R ASP 162 R VAL 163 R CYS 166 R THR 167 R ILE 213 R LEU 232 R ILE 233 R TYR 239 R SER 243 R THR 244 R ALA 247 R TRP 340 R PHE 343 R PHE 344 R SER 347 R LEU 370 |
| N1=C(SCCC1)Nc1c(cc(N)cc1C)C | 7.138 | R PHE 158 R ILE 159 R ASP 162 R VAL 163 R CYS 166 R THR 167 R ILE 213 R LEU 232 R ILE 233 R SER 243 R THR 244 R ALA 247 R PHE 343 R PHE 344 R SER 347 R LEU 370 |
| N1=C(SCCC1)Nc1c(CCC)cccc1 | 7.135 | R PHE 158 R ILE 159 R ASP 162 R VAL 163 R CYS 166 R THR 167 R ILE 213 R LEU 232 R ILE 233 R SER 243 R ALA 247 R TRP 340 R PHE 343 R PHE 344 R SER 347 R ARG 350 |
| N1=C(SCCC1)Nc1c(OC(F)F)cccc1 | 7.134 | R ASP 162 R VAL 163 R THR 167 R ILE 213 R LEU 232 R ILE 233 R THR 240 R SER 243 R THR 244 R ALA 247 R PHE 343 R PHE 344 R SER 347 R THR 348 R ARG 350 R LEU 370 |
| N1=C(SCCC1)Nc1c(cccc1C)C | 7.132 | R ASP 162 R VAL 163 R CYS 166 R THR 167 R ILE 213 R ILE 233 R SER 234 R THR 240 R SER 243 R THR 244 R ALA 247 R PHE 343 R PHE 344 R SER 347 R THR 348 |
| N1=C(SCCC1)Nc1c(Br)c(ccc1)C | 7.13 | R PHE 158 R ILE 159 R ASP 162 R VAL 163 R CYS 166 R THR 167 R ILE 213 R LEU 232 R ILE 233 R SER 243 R THR 244 R ALA 247 R PHE 343 R PHE 344 R SER 347 |
| N1=C(SCCC1)Nc1cc(ccc1F)C | 7.128 | R ASP 162 R VAL 163 R CYS 166 R THR 167 R ILE 213 R LEU 232 R ILE 233 R GLN 235 R SER 243 R THR 244 R ALA 247 R PHE 343 R PHE 344 R SER 347 R ARG 350 |
| N1=C(SCCC1)Nc1cc(Cl)c(cc1Cl)C | 7.127 | R PHE 158 R ILE 159 R ASP 162 R VAL 163 R CYS 166 R THR 167 R ILE 213 R CYS 231 R LEU 232 R ILE 233 R SER 243 R THR 244 R ALA 247 R TRP 340 R PHE 343 R PHE 344 R ARG 367 R LEU 370 |
| N1=C(SCCC1)NCc1c(OC)cc(C)cc1 | 7.125 | R PHE 158 R ILE 159 R ASP 162 R VAL 163 R THR 167 R ILE 213 R CYS 231 R LEU 232 R ILE 233 R GLN 235 R THR 240 R SER 243 R THR 244 R ALA 247 R PHE 343 R PHE 344 R SER 347 R ARG 350 |
| N1=C(SCCC1)Nc1c(N2CCCC2)cccc1 | 7.123 | R ASP 162 R VAL 163 R CYS 166 R THR 167 R ILE 213 R LEU 232 R ILE 233 R TYR 239 R THR 240 R SER 243 R THR 244 R ALA 247 R PHE 343 R PHE 344 R SER 347 R LEU 370 |
| N1=C(SCCC1)Nc1c(O)cc(c(c1)C)C | 7.113 | R ASP 162 R VAL 163 R ILE 213 R LEU 232 R ILE 233 R GLN 235 R SER 243 R THR 244 R ALA 247 R PHE 343 R PHE 344 R SER 347 R ARG 350 R LEU 370 |
| N1=C(SCCC1)Nc1cc(C(C)C)ccc1O | 7.109 | R PHE 158 R ILE 159 R ASP 162 R VAL 163 R CYS 166 R ILE 213 R CYS 231 R LEU 232 R ILE 233 R TYR 239 R SER 243 R THR 244 R ALA 247 R PHE 343 R PHE 344 R SER 347 R ARG 367 R LEU 370 R TYR 374 |
| N1=C(SCCC1)Nc1c(F)cc(F)cc1Cl | 7.104 | R ASP 162 R VAL 163 R CYS 166 R THR 167 R ILE 213 R LEU 232 R ILE 233 R GLN 235 R SER 243 R THR 244 R ALA 247 R PHE 343 R PHE 344 R SER 347 R ARG 350 R LEU 370 |
| N1=C(SCCC1)Nc1c(c(Br)ccc1)C | 7.102 | R PHE 158 R ILE 159 R ASP 162 R VAL 163 R CYS 166 R THR 167 R LEU 232 R ILE 233 R SER 243 R THR 244 R ALA 247 R PHE 343 R PHE 344 R SER 347 R LEU 370 |
| N1=C(SCCC1)Nc1c2c([nH]cc2)ccc1 | 7.1 | R PHE 158 R ILE 159 R ASP 162 R VAL 163 R LEU 232 R ILE 233 R PHE 343 R SER 347 R ARG 350 R LEU 363 R GLU 366 R ARG 367 R LEU 370 |
| N1=C(SCCC1)Nc1c(cc(N(C)C)cc1)C | 7.092 | R ILE 159 R ASP 162 R VAL 163 R THR 167 R ILE 213 R LEU 232 R ILE 233 R SER 243 R THR 244 R ALA 247 R PHE 343 R PHE 344 R SER 347 R ARG 350 R GLU 366 R ARG 367 R LEU 370 |
| N1=C(SCCC1)Nc1c2n(ncc2)ccc1 | 7.087 | R ILE 159 R ASP 162 R VAL 163 R THR 167 R ILE 213 R LEU 232 R ILE 233 R SER 243 R THR 244 R ALA 247 R PHE 343 R PHE 344 R SER 347 R ARG 350 |
| N1=C(SCCC1)Nc1c(F)c(ccc1F)C | 7.083 | R PHE 158 R ILE 159 R ASP 162 R VAL 163 R CYS 166 R LEU 232 R ILE 233 R SER 243 R THR 244 R ALA 247 R PHE 343 R PHE 344 R SER 347 |
| N1=C(SCCC1)Nc1c(OC(C)C)cccc1 | 7.083 | R PHE 158 R ILE 159 R ASP 162 R VAL 163 R CYS 231 R LEU 232 R ILE 233 R GLN 235 R PHE 343 R PHE 344 R SER 347 R ARG 350 R LEU 370 |
| N1=C(SCCC1)Nc1c([C@H](O)C)cccc1 | 7.079 | R ILE 159 R ASP 162 R VAL 163 R CYS 166 R THR 167 R ILE 213 R LEU 232 R ILE 233 R SER 234 R TYR 239 R THR 240 R SER 243 R THR 244 R ALA 247 R PHE 343 R PHE 344 R SER 347 R LEU 370 |
| N1=C(SCCC1)Nc1c(OC)cccc1OC | 7.078 | R ILE 159 R ASP 162 R VAL 163 R CYS 166 R THR 167 R ILE 213 R LEU 232 R ILE 233 R TYR 239 R THR 240 R SER 243 R THR 244 R ALA 247 R PHE 343 R PHE 344 R SER 347 R LEU 370 |
| N1=C(SCCC1)Nc1c(OCC)cc(cc1)C | 7.078 | R PHE 158 R ILE 159 R ASP 162 R VAL 163 R CYS 231 R LEU 232 R ILE 233 R GLN 235 R PHE 343 R PHE 344 R SER 347 R ARG 350 R LEU 370 |
| N1=C(SCCC1)Nc1cc(c(nc1C)C)C | 7.077 | R ILE 159 R ASP 162 R VAL 163 R THR 167 R ILE 213 R LEU 232 R ILE 233 R SER 243 R THR 244 R ALA 247 R PHE 343 R PHE 344 R SER 347 R ARG 350 R LEU 370 |
| N1=C(SCCC1)Nc1c(cc(Cl)cc1OC)C | 7.075 | R ILE 159 R ASP 162 R VAL 163 R CYS 166 R THR 167 R ILE 213 R LEU 232 R ILE 233 R GLN 235 R SER 243 R THR 244 R ALA 247 R PHE 343 R PHE 344 R SER 347 R ARG 350 R LEU 370 |
| N1=C(SCCC1)Nc1cc(CC)ccc1O | 7.075 | R PHE 158 R ILE 159 R ASP 162 R VAL 163 R CYS 166 R ILE 213 R LEU 232 R ILE 233 R SER 243 R THR 244 R ALA 247 R PHE 343 R PHE 344 R SER 347 R LEU 370 |
| N1=C(SCCC1)Nc1c(F)cc(F)c(F)c1 | 7.073 | R PHE 158 R ILE 159 R ASP 162 R VAL 163 R LEU 232 R ILE 233 R TYR 239 R SER 243 R THR 244 R PHE 343 R PHE 344 R SER 347 R LEU 370 |
| N1=C(SCCC1)Nc1cc(OC(F)F)ccc1 | 7.073 | R PHE 158 R ILE 159 R ASP 162 R VAL 163 R LEU 232 R ILE 233 R PHE 343 R LEU 346 R SER 347 R ARG 350 R GLU 366 R ARG 367 R PHE 369 R LEU 370 |
| N1=C(SCCC1)Nc1c(c(Cl)cnc1)C | 7.071 | R ILE 159 R ASP 162 R VAL 163 R CYS 166 R THR 167 R ILE 213 R LEU 232 R ILE 233 R SER 243 R THR 244 R ALA 247 R PHE 343 R PHE 344 R SER 347 R LEU 370 |
| N1=C(SCCC1)Nc1c(ccc(N)c1)C | 7.07 | R PHE 158 R ILE 159 R ASP 162 R VAL 163 R CYS 166 R THR 167 R ILE 213 R LEU 232 R ILE 233 R THR 240 R SER 243 R THR 244 R ALA 247 R PHE 343 R PHE 344 R SER 347 |
| N1=C(SCCC1)Cc1c(cc(cc1C)C)C | 7.069 | R PHE 158 R ILE 159 R ASP 162 R VAL 163 R CYS 166 R THR 167 R ILE 213 R LEU 232 R ILE 233 R SER 243 R THR 244 R ALA 247 R TRP 340 R PHE 343 R PHE 344 R SER 347 R LEU 370 |
| N1=C(SCCC1)[C@H]1NCCc2c1cccc2 | 7.066 | R PHE 158 R ILE 159 R ASP 162 R VAL 163 R CYS 166 R THR 167 R ILE 213 R LEU 232 R ILE 233 R THR 240 R SER 243 R THR 244 R ALA 247 R TRP 340 R PHE 343 R PHE 344 R SER 347 |
| N1=C(SCCC1)Nc1c2[nH]ccc2ccc1 | 7.06 | R PHE 158 R ILE 159 R ASP 162 R VAL 163 R LEU 232 R ILE 233 R PHE 343 R SER 347 R ARG 350 R GLU 366 R ARG 367 R LEU 370 |
| N1=C(SCCC1)NCc1c(F)cc(F)cc1 | 7.058 | R PHE 158 R ILE 159 R ASP 162 R VAL 163 R CYS 166 R THR 167 R ILE 213 R CYS 231 R LEU 232 R ILE 233 R SER 243 R THR 244 R ALA 247 R PHE 343 R PHE 344 R SER 347 |
| N1=C(SCCC1)Nc1c(C)ccnc1Cl | 7.055 | R ILE 159 R ASP 162 R VAL 163 R CYS 166 R THR 167 R ILE 213 R LEU 232 R ILE 233 R THR 240 R SER 243 R THR 244 R ALA 247 R PHE 343 R PHE 344 R SER 347 R LEU 370 |
| N1=C(SCCC1)Nc1c(F)cc(F)cc1F | 7.055 | R ASP 162 R VAL 163 R CYS 166 R THR 167 R ILE 213 R LEU 232 R ILE 233 R SER 243 R THR 244 R ALA 247 R PHE 343 R PHE 344 R SER 347 R ARG 350 R LEU 370 |
| N1=C(SCCC1)N[C@H]1[C@@H](C[C@@H](CC1)C)C | 7.05 | R PHE 158 R ILE 159 R ASP 162 R VAL 163 R CYS 166 R THR 167 R ILE 213 R LEU 232 R ILE 233 R SER 243 R THR 244 R ALA 247 R PHE 343 R PHE 344 R SER 347 R LEU 370 |
| N1=C(SCCC1)N[C@@H](c1c(nn(c1C)C)C)C | 7.05 | R PHE 158 R ILE 159 R ASP 162 R VAL 163 R CYS 166 R THR 167 R ILE 213 R LEU 232 R ILE 233 R SER 243 R THR 244 R ALA 247 R TRP 340 R PHE 343 R PHE 344 R SER 347 R LEU 370 |
| N1=C(SCCC1)Nc1c(cccc1Cl)C | 7.039 | R ILE 159 R ASP 162 R VAL 163 R CYS 166 R THR 167 R ILE 213 R LEU 232 R ILE 233 R SER 243 R THR 244 R ALA 247 R PHE 343 R PHE 344 R SER 347 R LEU 370 |
| N1=C(SCCC1)N[C@H](c1c(CC)cncc1)C | 7.035 | R PHE 158 R ILE 159 R ASP 162 R VAL 163 R CYS 166 R THR 167 R ILE 213 R LEU 232 R ILE 233 R SER 243 R THR 244 R ALA 247 R TRP 340 R PHE 343 R PHE 344 R SER 347 R LEU 370 |
| N1=C(SCCC1)Nc1c(cc(cc1Cl)C)C | 7.031 | R PHE 158 R ILE 159 R ASP 162 R VAL 163 R CYS 166 R THR 167 R ILE 213 R LEU 232 R ILE 233 R SER 243 R THR 244 R ALA 247 R PHE 343 R PHE 344 R SER 347 R LEU 370 |
| N1=C(SCCC1)N[C@H](c1c(F)cccc1)C | 7.027 | R PHE 158 R ILE 159 R ASP 162 R VAL 163 R CYS 166 R THR 167 R ILE 213 R LEU 232 R ILE 233 R SER 243 R ALA 247 R PHE 343 R PHE 344 R SER 347 R LEU 370 |
| N1=C(SCCC1)Nc1c(N)ccc(Br)c1 | 7.025 | R ASP 162 R VAL 163 R CYS 166 R THR 167 R ILE 213 R LEU 232 R ILE 233 R THR 240 R SER 243 R THR 244 R ALA 247 R PHE 343 R PHE 344 R SER 347 R LEU 370 |
| N1=C(SCCC1)NCc1cc(N)c(Cl)cc1 | 7.025 | R PHE 158 R ILE 159 R ASP 162 R VAL 163 R THR 167 R ILE 213 R CYS 231 R LEU 232 R ILE 233 R SER 243 R THR 244 R ALA 247 R PHE 343 R PHE 344 R SER 347 R LEU 370 |
| N1=C(SCCC1)Nc1c(N)c(ccc1)C#N | 7.024 | R PHE 158 R ASP 162 R VAL 163 R ILE 213 R LEU 232 R ILE 233 R SER 243 R THR 244 R ALA 247 R PHE 343 R PHE 344 R SER 347 R ARG 350 |
| N1=C(SCCC1)Nc1c(ccc(Br)c1)C | 7.022 | R PHE 158 R ILE 159 R ASP 162 R VAL 163 R CYS 166 R LEU 232 R ILE 233 R SER 243 R THR 244 R ALA 247 R PHE 343 R PHE 344 R SER 347 R LEU 370 |
| N1=C(SCCC1)NC1CCC2(CC1)CC2 | 7.021 | R PHE 158 R ILE 159 R ASP 162 R VAL 163 R THR 167 R ILE 213 R LEU 232 R ILE 233 R SER 243 R THR 244 R ALA 247 R PHE 343 R PHE 344 R SER 347 R LEU 370 |
| N1=C(SCCC1)NCc1ccc(F)cc1 | 7.008 | R PHE 158 R ILE 159 R ASP 162 R VAL 163 R THR 167 R ILE 213 R CYS 231 R LEU 232 R ILE 233 R SER 243 R THR 244 R ALA 247 R PHE 343 R PHE 344 R SER 347 |
| N1=C(SCCC1)Nc1cc2c([nH]cc2)cc1 | 7.001 | R VAL 138 R PHE 158 R ILE 159 R ASP 162 R VAL 163 R CYS 166 R CYS 231 R LEU 232 R ILE 233 R SER 243 R ALA 247 R PHE 343 R PHE 344 R ARG 367 R LEU 370 |
| N1=C(SCCC1)Nc1c(OC)cc(cc1)C | 6.998 | R PHE 158 R ILE 159 R ASP 162 R VAL 163 R CYS 231 R LEU 232 R ILE 233 R PHE 343 R PHE 344 R SER 347 R LEU 370 |
| N1=C(SCCC1)Nc1c(c(F)ccc1)C | 6.996 | R ILE 159 R ASP 162 R VAL 163 R CYS 166 R THR 167 R ILE 213 R LEU 232 R ILE 233 R SER 243 R THR 244 R ALA 247 R TRP 340 R PHE 343 R PHE 344 R SER 347 R LEU 370 |
| N1=C(SCCC1)Nc1c(ccc(F)c1)C | 6.992 | R PHE 158 R ILE 159 R ASP 162 R VAL 163 R CYS 166 R THR 167 R ILE 213 R LEU 232 R ILE 233 R SER 243 R THR 244 R ALA 247 R PHE 343 R PHE 344 R SER 347 |
| N1=C(SCCC1)N[n+]1c(cc(cc1C)C)C | 6.983 | R PHE 158 R ILE 159 R ASP 162 R VAL 163 R CYS 166 R THR 167 R ILE 213 R LEU 232 R ILE 233 R SER 243 R ALA 247 R TRP 340 R PHE 343 R PHE 344 R SER 347 R LEU 370 R TYR 374 |
| N1=C(SCCC1)Nc1c(cc(Br)cc1)C | 6.982 | R PHE 158 R ILE 159 R ASP 162 R VAL 163 R CYS 166 R ILE 213 R LEU 232 R ILE 233 R SER 243 R THR 244 R ALA 247 R PHE 343 R PHE 344 R SER 347 |
| N1=C(SCCC1)Nc1c(ccc(O)c1)C | 6.981 | R PHE 158 R ILE 159 R ASP 162 R VAL 163 R CYS 166 R THR 167 R ILE 213 R LEU 232 R ILE 233 R SER 243 R THR 244 R ALA 247 R PHE 343 R PHE 344 R SER 347 |
| N1=C(SCCC1)Nc1c(Br)cc(cc1)C | 6.981 | R PHE 158 R ILE 159 R ASP 162 R VAL 163 R CYS 231 R LEU 232 R ILE 233 R PHE 343 R PHE 344 R SER 347 |
| N1=C(SCCC1)Nc1c(O)ccc(Br)c1 | 6.979 | R PHE 158 R ILE 159 R ASP 162 R VAL 163 R CYS 166 R LEU 232 R ILE 233 R SER 243 R THR 244 R ALA 247 R PHE 343 R PHE 344 R SER 347 |
| N1=C(SCCC1)Nc1c2ncoc2ccc1 | 6.976 | R ASP 162 R VAL 163 R THR 167 R ILE 213 R LEU 232 R ILE 233 R GLN 235 R SER 243 R THR 244 R ALA 247 R PHE 343 R PHE 344 R SER 347 R ARG 350 R LEU 370 |
| N1=C(SCCC1)Nc1c(O)c(cc(c1)C)C | 6.976 | R ILE 159 R ASP 162 R VAL 163 R ILE 213 R LEU 232 R ILE 233 R GLN 235 R SER 243 R THR 244 R ALA 247 R PHE 343 R PHE 344 R SER 347 R ARG 350 R LEU 370 |
| N1=C(SCCC1)N1Cc2c(OC)cccc2C1 | 6.973 | R PHE 158 R ASP 162 R VAL 163 R THR 167 R ILE 213 R LEU 232 R ILE 233 R THR 240 R SER 243 R THR 244 R ALA 247 R PHE 343 R PHE 344 R SER 347 R LEU 370 |
| N1=C(SCCC1)Nc1c(C(C)C)cc(O)c(c1)C | 6.97 | R ILE 159 R ASP 162 R VAL 163 R THR 167 R ILE 213 R LEU 232 R ILE 233 R SER 243 R THR 244 R ALA 247 R PHE 343 R PHE 344 R SER 347 R ARG 350 R LEU 370 |
| N1=C(SCCC1)Nc1c2nc[nH]c2ccc1 | 6.968 | R ASP 162 R VAL 163 R ILE 213 R LEU 232 R ILE 233 R GLN 235 R SER 243 R THR 244 R ALA 247 R PHE 343 R PHE 344 R SER 347 R ARG 350 R LEU 370 |
| N1=C(SCCC1)Nc1c(ccn(c1=O)C)C | 6.961 | R ILE 159 R ASP 162 R VAL 163 R CYS 166 R THR 167 R ILE 213 R LEU 232 R ILE 233 R THR 240 R SER 243 R THR 244 R ALA 247 R PHE 343 R PHE 344 R SER 347 R LEU 370 |
| N1=C(SCCC1)Nc1c(c(Cl)ccc1)C | 6.96 | R PHE 158 R ILE 159 R ASP 162 R VAL 163 R CYS 166 R THR 167 R CYS 231 R LEU 232 R ILE 233 R SER 243 R TRP 340 R PHE 343 R PHE 344 R ARG 367 R LEU 370 |
| N1=C(SCCC1)N[C@@H](c1c(OC)cccc1)C | 6.96 | R PHE 158 R ILE 159 R ASP 162 R VAL 163 R CYS 166 R THR 167 R ILE 213 R LEU 232 R ILE 233 R TYR 239 R SER 243 R THR 244 R ALA 247 R TRP 340 R PHE 343 R PHE 344 R SER 347 R LEU 370 |
| N1=C(SCCC1)Nc1c(cc(Cl)cc1)C | 6.954 | R PHE 158 R ILE 159 R ASP 162 R VAL 163 R CYS 231 R LEU 232 R ILE 233 R THR 244 R PHE 343 R PHE 344 R SER 347 R LEU 370 |
| N1=C(SCCC1)Nc1c2[nH]ncc2ccc1 | 6.953 | R ASP 162 R VAL 163 R ILE 213 R LEU 232 R ILE 233 R GLN 235 R THR 240 R SER 243 R THR 244 R ALA 247 R PHE 343 R PHE 344 R SER 347 R ARG 350 R LEU 370 |
| N1=C(SCCC1)Nc1c(ccc(Cl)c1)C | 6.952 | R PHE 158 R ILE 159 R ASP 162 R VAL 163 R CYS 166 R THR 167 R ILE 213 R LEU 232 R ILE 233 R SER 243 R THR 244 R ALA 247 R PHE 343 R PHE 344 R SER 347 |
| N1=C(SCCC1)Nc1cc(c(F)cc1Cl)C | 6.946 | R PHE 158 R ILE 159 R ASP 162 R VAL 163 R CYS 166 R CYS 231 R LEU 232 R ILE 233 R TYR 239 R SER 243 R TRP 340 R PHE 343 R PHE 344 R SER 347 R ARG 367 R LEU 370 |
| N1=C(SCCC1)Nc1c(cccc1F)C | 6.943 | R ILE 159 R ASP 162 R VAL 163 R CYS 166 R THR 167 R ILE 213 R LEU 232 R ILE 233 R THR 240 R SER 243 R THR 244 R ALA 247 R PHE 343 R PHE 344 R SER 347 R LEU 370 |
| N1=C(SCCC1)Nc1c(NC(C)C)cccc1F | 6.943 | R VAL 138 R ASP 142 R PHE 158 R ILE 159 R ASP 162 R VAL 163 R CYS 231 R LEU 232 R ILE 233 R PHE 343 R SER 347 R ARG 350 R LEU 363 R GLU 366 R ARG 367 R LEU 370 |
| N1=C(SCCC1)Nc1cc(ccc1)C#C | 6.929 | R ASP 162 R VAL 163 R ILE 213 R LEU 232 R ILE 233 R SER 243 R THR 244 R ALA 247 R PHE 343 R PHE 344 R SER 347 R ARG 350 R GLU 366 R LEU 370 |
| N1=C(SCCC1)Nc1c(OC(C)(C)C)cccc1 | 6.917 | R PHE 158 R ASP 162 R VAL 163 R CYS 166 R THR 167 R LEU 232 R ILE 233 R SER 234 R TYR 239 R THR 240 R SER 243 R THR 244 R ALA 247 R PHE 343 R PHE 344 R SER 347 R ARG 367 R LEU 370 R TYR 374 |
| N1=C(SCCC1)Nc1cc(c(F)cc1)C | 6.915 | R ASP 162 R VAL 163 R ILE 213 R LEU 232 R ILE 233 R GLN 235 R SER 243 R THR 244 R ALA 247 R PHE 343 R PHE 344 R SER 347 R ARG 350 R LEU 370 |
| N1=C(SCCC1)N[C@H]1CCCn2nccc12 | 6.914 | R PHE 158 R ILE 159 R ASP 162 R VAL 163 R ILE 213 R LEU 232 R ILE 233 R THR 240 R SER 243 R THR 244 R ALA 247 R PHE 343 R PHE 344 R SER 347 R LEU 370 |
| N1=C(SCCC1)Oc1c(CN)cc(Cl)cc1 | 6.913 | R ASP 162 R VAL 163 R CYS 166 R THR 167 R ILE 170 R ILE 213 R ILE 233 R SER 234 R TYR 239 R THR 240 R SER 243 R THR 244 R ALA 247 R TRP 340 R PHE 343 R PHE 344 R SER 347 R LEU 370 |
| N1=C(SCCC1)NCc1ccc(Cl)cc1 | 6.901 | R PHE 158 R ILE 159 R ASP 162 R VAL 163 R THR 167 R ILE 213 R CYS 231 R LEU 232 R ILE 233 R SER 243 R THR 244 R ALA 247 R PHE 343 R PHE 344 R SER 347 R LEU 370 |
| N1=C(SCCC1)Nc1c(SC(F)F)cccc1 | 6.899 | R ASP 162 R VAL 163 R CYS 166 R THR 167 R ILE 213 R LEU 232 R ILE 233 R SER 234 R TYR 239 R THR 240 R SER 243 R THR 244 R ALA 247 R PHE 343 R PHE 344 R SER 347 R LEU 370 |
| N1=C(SCCC1)Nc1c(SC(C)C)cccc1 | 6.897 | R PHE 158 R ILE 159 R ASP 162 R VAL 163 R CYS 231 R LEU 232 R ILE 233 R GLN 235 R PHE 343 R PHE 344 R SER 347 R ARG 350 R GLU 366 R LEU 370 |
| N1=C(SCCC1)Nc1c(cc(nc1C)C)C | 6.895 | R PHE 158 R ILE 159 R ASP 162 R VAL 163 R CYS 166 R THR 167 R ILE 213 R LEU 232 R ILE 233 R SER 243 R THR 244 R ALA 247 R PHE 343 R PHE 344 R SER 347 R LEU 370 |
| N1=C(SCCC1)Nc1c(Cl)cccc1F | 6.893 | R ILE 159 R ASP 162 R VAL 163 R CYS 166 R THR 167 R ILE 213 R LEU 232 R ILE 233 R SER 243 R THR 244 R ALA 247 R PHE 343 R PHE 344 R SER 347 R LEU 370 |
| N1=C(SCCC1)Nc1c(N)cc(F)cc1 | 6.887 | R PHE 158 R ILE 159 R ASP 162 R VAL 163 R CYS 166 R CYS 231 R LEU 232 R ILE 233 R TRP 340 R PHE 343 R PHE 344 R ARG 367 R LEU 370 R TYR 374 |
| N1=C(SCCC1)Nc1c(CC)cccc1 | 6.886 | R ILE 159 R ASP 162 R VAL 163 R CYS 166 R THR 167 R ILE 213 R LEU 232 R ILE 233 R SER 243 R THR 244 R ALA 247 R TRP 340 R PHE 343 R PHE 344 R SER 347 R LEU 370 |
| N1=C(SCCC1)Nc1c(F)cc(N)cc1F | 6.884 | R ASP 162 R VAL 163 R CYS 166 R THR 167 R ILE 213 R LEU 232 R ILE 233 R GLN 235 R SER 243 R THR 244 R ALA 247 R PHE 343 R PHE 344 R SER 347 R ARG 350 R LEU 370 |
| N1=C(SCCC1)N(c1c(cccc1C)C)C | 6.88 | R ASP 162 R VAL 163 R CYS 166 R THR 167 R ILE 213 R ILE 233 R SER 234 R TYR 239 R THR 240 R SER 243 R THR 244 R ALA 247 R TRP 340 R PHE 343 R PHE 344 R SER 347 |
| N1=C(SCCC1)Nc1c(N)c2ncsc2cc1 | 6.879 | R PHE 158 R ASP 162 R VAL 163 R ILE 213 R LEU 232 R ILE 233 R SER 243 R THR 244 R ALA 247 R PHE 343 R PHE 344 R SER 347 R ARG 350 R LEU 370 |
| N1=C(SCCC1)Nc1c(ccc(OC)c1)C | 6.874 | R ILE 159 R ASP 162 R VAL 163 R ILE 213 R LEU 232 R ILE 233 R GLN 235 R THR 240 R SER 243 R THR 244 R ALA 247 R PHE 343 R PHE 344 R SER 347 R ARG 350 R LEU 370 |
| N1=C(SCCC1)Nc1c(O)cc(c(Cl)c1)C | 6.873 | R ASP 162 R VAL 163 R CYS 166 R THR 167 R ILE 213 R LEU 232 R ILE 233 R SER 243 R THR 244 R ALA 247 R PHE 343 R PHE 344 R SER 347 R ARG 367 R LEU 370 R TYR 374 |
| N1=C(SCCC1)Nc1ncnc(N)c1OC | 6.873 | R VAL 163 R CYS 166 R THR 167 R ILE 213 R LEU 232 R ILE 233 R GLN 235 R THR 240 R SER 243 R THR 244 R ALA 247 R PHE 343 R PHE 344 R SER 347 R ARG 350 R LEU 370 |
| N1=C(SCCC1)Nc1c(cc(OCC)cc1)C | 6.868 | R ILE 159 R ASP 162 R VAL 163 R CYS 166 R THR 167 R ILE 213 R LEU 232 R ILE 233 R SER 243 R THR 244 R ALA 247 R PHE 343 R PHE 344 R SER 347 R ARG 350 R GLU 366 R ARG 367 R LEU 370 |
| N1=C(SCCC1)Nc1cc2OCOc2cc1 | 6.868 | R PHE 158 R ASP 162 R VAL 163 R ILE 213 R LEU 232 R ILE 233 R SER 243 R THR 244 R ALA 247 R PHE 343 R PHE 344 R SER 347 R ARG 350 R LEU 370 |
| N1=C(SCCC1)Nc1ccc(F)cc1Br | 6.867 | R PHE 158 R ILE 159 R ASP 162 R VAL 163 R CYS 231 R LEU 232 R ILE 233 R PHE 343 R PHE 344 R SER 347 |
| N1=C(SCCC1)Nc1c(F)cccc1F | 6.866 | R PHE 158 R ILE 159 R ASP 162 R VAL 163 R CYS 166 R THR 167 R LEU 232 R ILE 233 R PHE 343 R PHE 344 R SER 347 |
| N1=C(SCCC1)Nc1c(Cl)cc(F)cc1Cl | 6.866 | R PHE 158 R ILE 159 R ASP 162 R VAL 163 R CYS 166 R THR 167 R ILE 213 R LEU 232 R ILE 233 R SER 243 R THR 244 R ALA 247 R PHE 343 R PHE 344 R SER 347 R LEU 370 |
| N1=C(SCCC1)Nc1c(cc(OC)cc1)C | 6.856 | R PHE 158 R ILE 159 R ASP 162 R VAL 163 R CYS 166 R THR 167 R ILE 213 R LEU 232 R ILE 233 R SER 243 R THR 244 R ALA 247 R PHE 343 R PHE 344 R SER 347 R LEU 370 |
| N1=C(SCCC1)Nc1c(OC)ccc(N)c1 | 6.843 | R PHE 158 R ILE 159 R ASP 162 R VAL 163 R CYS 166 R LEU 232 R ILE 233 R SER 243 R THR 244 R ALA 247 R PHE 343 R PHE 344 R SER 347 |
| N1=C(SCCC1)Nc1c(CN(C)C)cccc1 | 6.843 | R PHE 158 R ILE 159 R ASP 162 R VAL 163 R CYS 231 R LEU 232 R ILE 233 R GLN 235 R PHE 343 R PHE 344 R SER 347 R ARG 350 R LEU 370 |
| N1=C(SCCC1)Nc1c(O)c(ccc1)C | 6.839 | R ASP 162 R VAL 163 R ILE 213 R LEU 232 R ILE 233 R GLN 235 R SER 243 R THR 244 R ALA 247 R PHE 343 R PHE 344 R SER 347 R ARG 350 R LEU 370 |
| N1=C(SCCC1)Nc1c(N)c(OCC)ccc1 | 6.837 | R PHE 158 R ILE 159 R ASP 162 R VAL 163 R ILE 213 R LEU 232 R ILE 233 R SER 243 R THR 244 R ALA 247 R PHE 343 R PHE 344 R SER 347 R ARG 350 |
| N1=C(SCCC1)N(c1cc(ccc1)C)C | 6.836 | R PHE 158 R ILE 159 R ASP 162 R VAL 163 R CYS 166 R THR 167 R ILE 213 R LEU 232 R ILE 233 R ALA 247 R TRP 340 R PHE 343 R PHE 344 R SER 347 |
| N1=C(SCCC1)Nc1c2nc(oc2ccc1)C | 6.83 | R PHE 158 R ILE 159 R ASP 162 R VAL 163 R ILE 213 R LEU 232 R ILE 233 R THR 240 R SER 243 R THR 244 R ALA 247 R PHE 343 R PHE 344 R SER 347 R ARG 350 R LEU 370 |
| N1=C(SCCC1)Nc1cc(cc(Br)c1)C | 6.829 | R ILE 159 R ASP 162 R VAL 163 R ILE 213 R LEU 232 R ILE 233 R SER 243 R THR 244 R ALA 247 R PHE 343 R PHE 344 R SER 347 R ARG 350 |
| N1=C(SCCC1)Nc1c(OC)cc(F)cc1 | 6.825 | R PHE 158 R ILE 159 R ASP 162 R VAL 163 R CYS 231 R LEU 232 R ILE 233 R GLN 235 R PHE 343 R PHE 344 R SER 347 R ARG 350 |
| N1=C(SCCC1)N1CCSc2nc(cc(c12)C)C | 6.821 | R PHE 158 R ILE 159 R ASP 162 R VAL 163 R CYS 166 R THR 167 R ILE 213 R LEU 232 R ILE 233 R ALA 247 R TRP 340 R PHE 343 R PHE 344 R SER 347 R LEU 370 |
| N1=C(SCCC1)Nc1cc(OC)c(N)cc1 | 6.817 | R ASP 162 R VAL 163 R CYS 166 R THR 167 R ILE 213 R LEU 232 R ILE 233 R SER 243 R THR 244 R ALA 247 R PHE 343 R PHE 344 R SER 347 R LEU 370 R TYR 374 |
| N1=C(SCCC1)Oc1c(C)cc(Cl)cc1C | 6.815 | R PHE 158 R ILE 159 R ASP 162 R VAL 163 R CYS 166 R THR 167 R ILE 213 R LEU 232 R ILE 233 R SER 243 R THR 244 R ALA 247 R TRP 340 R PHE 343 R PHE 344 R SER 347 |
| N1=C(SCCC1)Nc1c(N)ccc(c1)C | 6.814 | R ASP 162 R VAL 163 R ILE 213 R LEU 232 R ILE 233 R GLN 235 R SER 243 R THR 244 R ALA 247 R PHE 343 R PHE 344 R SER 347 R ARG 350 |
| N1=C(SCCC1)Nc1c(O)cc(N)cc1 | 6.814 | R PHE 158 R ILE 159 R ASP 162 R VAL 163 R CYS 166 R THR 167 R ILE 213 R LEU 232 R ILE 233 R SER 243 R ALA 247 R PHE 343 R PHE 344 R SER 347 R LEU 370 |
| N1=C(SCCC1)Nc1c(OC)cc(OC)c(Cl)c1 | 6.812 | R PHE 158 R ILE 159 R ASP 162 R VAL 163 R CYS 166 R THR 167 R ILE 213 R LEU 232 R ILE 233 R SER 243 R THR 244 R ALA 247 R PHE 343 R PHE 344 R SER 347 R LEU 370 |
| N1=C(SCCC1)Nc1ccc(Cl)cc1Cl | 6.809 | R PHE 158 R ILE 159 R ASP 162 R VAL 163 R CYS 231 R LEU 232 R ILE 233 R THR 244 R PHE 343 R PHE 344 R SER 347 |
| N1=C(SCCC1)Nc1c(Cl)c(Cl)c(Cl)cc1 | 6.807 | R VAL 138 R PHE 158 R ILE 159 R ASP 162 R VAL 163 R CYS 166 R CYS 231 R LEU 232 R ILE 233 R SER 243 R PHE 343 R PHE 344 R ARG 367 R LEU 370 |
| N1=C(SCCC1)Nc1c(C)cccc1 | 6.806 | R PHE 158 R ILE 159 R ASP 162 R VAL 163 R CYS 231 R LEU 232 R ILE 233 R PHE 343 R PHE 344 R SER 347 R LEU 370 |
| N1=C(SCCC1)Nc1cc(F)ccc1Cl | 6.805 | R PHE 158 R ILE 159 R ASP 162 R VAL 163 R CYS 166 R THR 167 R ILE 213 R LEU 232 R ILE 233 R SER 243 R THR 244 R ALA 247 R PHE 343 R PHE 344 R SER 347 |
| N1=C(SCCC1)N1[C@H]2[C@H](SCC1)CCCC2 | 6.802 | R PHE 158 R ILE 159 R ASP 162 R VAL 163 R CYS 166 R THR 167 R LEU 232 R ILE 233 R ALA 247 R TRP 340 R PHE 343 R PHE 344 R SER 347 R LEU 370 |
| N1=C(SCCC1)Nc1cc(cc(Cl)c1)C | 6.801 | R ILE 159 R ASP 162 R VAL 163 R ILE 213 R LEU 232 R ILE 233 R SER 243 R THR 244 R ALA 247 R PHE 343 R PHE 344 R SER 347 R ARG 350 |
| N1=C(SCCC1)C1Sc2c(S1)cccc2 | 6.801 | R PHE 158 R ILE 159 R ASP 162 R VAL 163 R CYS 166 R THR 167 R ILE 213 R LEU 232 R ILE 233 R SER 243 R THR 244 R ALA 247 R PHE 343 R PHE 344 R SER 347 |
| N1=C(SCCC1)Nc1c(N)cc(cc1)C#N | 6.8 | R ASP 162 R VAL 163 R THR 167 R ILE 213 R LEU 232 R ILE 233 R SER 243 R THR 244 R ALA 247 R PHE 343 R PHE 344 R SER 347 R ARG 350 R ARG 367 R LEU 370 |
| N1=C(SCCC1)Nc1c(ccnc1)C(F)(F)F | 6.799 | R PHE 158 R ILE 159 R ASP 162 R VAL 163 R LEU 232 R ILE 233 R PHE 343 R ARG 350 R LEU 363 R GLU 366 R ARG 367 R LEU 370 |
| N1=C(SCCC1)Nc1c(N)cc(Cl)c(F)c1 | 6.793 | R ASP 162 R VAL 163 R THR 167 R ILE 213 R LEU 232 R ILE 233 R GLN 235 R SER 243 R THR 244 R ALA 247 R PHE 343 R PHE 344 R SER 347 R ARG 350 R LEU 370 |
| N1=C(SCCC1)Nc1c(Cl)c(ccc1)C | 6.792 | R VAL 138 R PHE 158 R ILE 159 R ASP 162 R VAL 163 R CYS 166 R CYS 231 R LEU 232 R ILE 233 R TRP 340 R PHE 343 R PHE 344 R ARG 367 R LEU 370 |
| N1=C(SCCC1)Nc1cc(N)c(Cl)cc1 | 6.787 | R ASP 162 R VAL 163 R ILE 213 R LEU 232 R ILE 233 R SER 243 R THR 244 R ALA 247 R PHE 343 R PHE 344 R SER 347 R ARG 350 R LEU 370 |
| N1=C(SCCC1)Nc1c(ncc(F)c1)CC | 6.787 | R VAL 163 R THR 167 R ILE 213 R LEU 232 R ILE 233 R SER 234 R GLN 235 R TYR 239 R THR 240 R SER 243 R THR 244 R ALA 247 R PHE 343 R PHE 344 R SER 347 R ARG 350 R LEU 370 |
| N1=C(SCCC1)N(c1c(cc(cc1)C)C)C | 6.785 | R PHE 158 R ILE 159 R ASP 162 R VAL 163 R CYS 166 R CYS 231 R LEU 232 R ILE 233 R TYR 239 R THR 240 R SER 243 R THR 244 R PHE 343 R PHE 344 R SER 347 R LEU 370 |
| N1=C(SCCC1)N1[C@@H](c2c(C1)cccc2)C | 6.782 | R PHE 158 R ILE 159 R ASP 162 R VAL 163 R CYS 166 R THR 167 R ILE 213 R LEU 232 R ILE 233 R SER 243 R ALA 247 R PHE 343 R PHE 344 R SER 347 R LEU 370 |
| N1=C(SCCC1)NCc1c(O)cccc1 | 6.78 | R VAL 163 R CYS 166 R THR 167 R ILE 213 R LEU 232 R ILE 233 R GLN 235 R THR 240 R SER 243 R THR 244 R ALA 247 R PHE 343 R PHE 344 R SER 347 R ARG 350 R LEU 370 |
| N1=C(SCCC1)N(c1cc(C)ccc1C)C | 6.776 | R ASP 162 R VAL 163 R CYS 166 R THR 167 R ILE 213 R ILE 233 R SER 234 R TYR 239 R THR 240 R SER 243 R THR 244 R ALA 247 R TRP 340 R PHE 343 R PHE 344 R SER 347 R THR 348 |
| N1=C(SCCC1)Nc1c(OC)ccc(Cl)c1 | 6.775 | R PHE 158 R ILE 159 R ASP 162 R VAL 163 R CYS 166 R THR 167 R LEU 232 R ILE 233 R SER 243 R THR 244 R ALA 247 R TRP 340 R PHE 343 R PHE 344 R SER 347 R LEU 370 R TYR 374 |
| N1=C(SCCC1)Nc1c(Cl)ccc(Cl)c1 | 6.769 | R PHE 158 R ILE 159 R ASP 162 R VAL 163 R CYS 166 R THR 167 R LEU 232 R ILE 233 R SER 243 R THR 244 R ALA 247 R PHE 343 R PHE 344 R SER 347 |
| N1=C(SCCC1)Nc1cc(Cl)c(N)cc1 | 6.767 | R ASP 162 R VAL 163 R CYS 166 R THR 167 R ILE 213 R LEU 232 R ILE 233 R SER 243 R THR 244 R ALA 247 R PHE 343 R PHE 344 R SER 347 R LEU 370 R TYR 374 |
| N1=C(SCCC1)Cc1c(F)cccc1F | 6.766 | R ASP 162 R VAL 163 R CYS 166 R THR 167 R ILE 213 R ILE 233 R SER 234 R TYR 239 R THR 240 R SER 243 R THR 244 R ALA 247 R TRP 340 R PHE 343 R PHE 344 R SER 347 |
| N1=C(SCCC1)NCc1c(F)cccc1 | 6.765 | R PHE 158 R ILE 159 R ASP 162 R VAL 163 R CYS 166 R THR 167 R ILE 213 R LEU 232 R ILE 233 R SER 243 R THR 244 R ALA 247 R PHE 343 R PHE 344 R SER 347 |
| N1=C(SCCC1)Nc1c(cc(N)cc1)C | 6.764 | R ASP 162 R VAL 163 R CYS 166 R THR 167 R ILE 213 R LEU 232 R ILE 233 R SER 243 R ALA 247 R PHE 343 R PHE 344 R SER 347 R LEU 370 R TYR 374 |
| N1=C(SCCC1)NCc1c(Br)cccc1 | 6.763 | R PHE 158 R ILE 159 R ASP 162 R VAL 163 R CYS 166 R THR 167 R ILE 213 R CYS 231 R LEU 232 R ILE 233 R SER 243 R ALA 247 R PHE 343 R PHE 344 R SER 347 R LEU 370 |
| N1=C(SCCC1)Nc1c(N)cc(F)c(OC)c1 | 6.759 | R ASP 162 R VAL 163 R CYS 166 R THR 167 R ILE 213 R LEU 232 R ILE 233 R THR 240 R SER 243 R THR 244 R ALA 247 R PHE 343 R PHE 344 R SER 347 R ARG 367 R LEU 370 R TYR 374 |
| N1=C(SCCC1)Nc1c(N)cc(Cl)c(Cl)c1 | 6.754 | R ASP 162 R VAL 163 R THR 167 R ILE 213 R LEU 232 R ILE 233 R GLN 235 R SER 243 R THR 244 R ALA 247 R PHE 343 R PHE 344 R SER 347 R ARG 350 R LEU 370 |
| N1=C(SCCC1)N(c1c(N)cccc1)C | 6.753 | R ASP 162 R VAL 163 R CYS 166 R THR 167 R ILE 213 R ILE 233 R SER 234 R TYR 239 R THR 240 R SER 243 R THR 244 R ALA 247 R TRP 340 R PHE 343 R PHE 344 R SER 347 |
| N1=C(SCCC1)Nc1c(F)c(ccc1)C | 6.75 | R VAL 138 R PHE 158 R ILE 159 R ASP 162 R VAL 163 R CYS 166 R CYS 231 R LEU 232 R ILE 233 R TRP 340 R PHE 343 R PHE 344 R ARG 367 R LEU 370 |
| N1=C(SCCC1)Nc1c(ccnc1C)C | 6.748 | R ASP 162 R VAL 163 R CYS 166 R THR 167 R ILE 213 R ILE 233 R SER 234 R THR 240 R SER 243 R THR 244 R ALA 247 R PHE 343 R PHE 344 R SER 347 |
| N1=C(SCCC1)N[C@@H]1[C@H](CCC[C@H]1C)C | 6.742 | R PHE 158 R ILE 159 R ASP 162 R VAL 163 R CYS 166 R THR 167 R ILE 213 R LEU 232 R ILE 233 R TYR 239 R SER 243 R THR 244 R ALA 247 R TRP 340 R PHE 343 R PHE 344 R SER 347 |
| N1=C(SCCC1)N(CC)c1ccc(cc1)C | 6.742 | R PHE 158 R ILE 159 R ASP 162 R VAL 163 R LEU 232 R ILE 233 R PHE 343 R PHE 344 R SER 347 R ARG 350 R GLU 366 R ARG 367 R LEU 370 |
| N1=C(SCCC1)Nc1c(Cl)cc(Cl)cc1Cl | 6.741 | R PHE 158 R ILE 159 R ASP 162 R VAL 163 R CYS 166 R ILE 213 R LEU 232 R ILE 233 R SER 243 R THR 244 R ALA 247 R PHE 343 R PHE 344 R SER 347 |
| N1=C(SCCC1)Nc1c(OC)cc(OC)cc1 | 6.734 | R PHE 158 R ILE 159 R ASP 162 R VAL 163 R CYS 166 R THR 167 R ILE 213 R LEU 232 R ILE 233 R SER 243 R THR 244 R ALA 247 R TRP 340 R PHE 343 R PHE 344 R SER 347 |
| N1=C(SCCC1)Cc1c([nH+]c(N)cc1C)C | 6.732 | R PHE 158 R ILE 159 R ASP 162 R VAL 163 R CYS 166 R THR 167 R ILE 213 R LEU 232 R ILE 233 R SER 243 R ALA 247 R TRP 340 R PHE 343 R PHE 344 R SER 347 R LEU 370 |
| N1=C(SCCC1)Nc1c(OCC)cccc1 | 6.731 | R ILE 159 R ASP 162 R VAL 163 R CYS 166 R THR 167 R ILE 213 R LEU 232 R ILE 233 R SER 243 R THR 244 R ALA 247 R TRP 340 R PHE 343 R PHE 344 R SER 347 R LEU 370 |
| N1=C(SCCC1)Nc1c(OC)cc(F)c(F)c1 | 6.73 | R ASP 162 R VAL 163 R CYS 166 R THR 167 R LEU 232 R ILE 233 R GLN 235 R THR 240 R SER 243 R THR 244 R ALA 247 R PHE 343 R PHE 344 R SER 347 R ARG 350 R LEU 370 |
| N1=C(SCCC1)Oc1c(C)cc(F)cc1C | 6.729 | R PHE 158 R ILE 159 R ASP 162 R VAL 163 R CYS 166 R THR 167 R ILE 213 R LEU 232 R ILE 233 R SER 243 R ALA 247 R TRP 340 R PHE 343 R PHE 344 R SER 347 R LEU 370 |
| N1=C(SCCC1)Nc1cc(ccc1Cl)C | 6.727 | R PHE 158 R ILE 159 R ASP 162 R VAL 163 R LEU 232 R ILE 233 R PHE 343 R SER 347 R ARG 350 R LEU 363 R GLU 366 R ARG 367 R LEU 370 |
| N1=C(SCCC1)Nc1c(Cl)cccc1Cl | 6.72 | R ILE 159 R ASP 162 R VAL 163 R CYS 166 R THR 167 R ILE 213 R LEU 232 R ILE 233 R SER 243 R THR 244 R ALA 247 R PHE 343 R PHE 344 R SER 347 R LEU 370 |
| N1=C(SCCC1)Nc1c(O)cc(cc1)C | 6.71 | R PHE 158 R ILE 159 R ASP 162 R VAL 163 R CYS 166 R CYS 231 R LEU 232 R ILE 233 R SER 243 R THR 244 R PHE 343 R PHE 344 R ARG 367 R LEU 370 R TYR 374 |
| N1=C(SCCC1)N[C@H](c1c(F)cncc1F)C | 6.709 | R PHE 158 R ILE 159 R ASP 162 R VAL 163 R THR 167 R ILE 213 R LEU 232 R ILE 233 R TYR 239 R SER 243 R ALA 247 R PHE 343 R PHE 344 R SER 347 R LEU 370 R TYR 374 |
| N1=C(SCCC1)Nc1c(N)ccc(F)c1F | 6.708 | R PHE 158 R ILE 159 R ASP 162 R VAL 163 R LEU 232 R ILE 233 R PHE 343 R SER 347 R ARG 350 R LEU 363 R GLU 366 R ARG 367 R LEU 370 |
| N1=C(SCCC1)Nc1c(O)ccc(F)c1 | 6.706 | R PHE 158 R ILE 159 R ASP 162 R VAL 163 R CYS 166 R LEU 232 R ILE 233 R THR 244 R ALA 247 R PHE 343 R PHE 344 R SER 347 |
| N1=C(SCCC1)C1Oc2c(O1)cccc2 | 6.698 | R ASP 162 R VAL 163 R CYS 166 R THR 167 R ILE 213 R ILE 233 R SER 234 R TYR 239 R THR 240 R SER 243 R THR 244 R ALA 247 R TRP 340 R PHE 343 R PHE 344 R SER 347 |
| N1=C(SCCC1)Nc1c(nc(O)nc1O)C | 6.697 | R PHE 158 R ILE 159 R ASP 162 R VAL 163 R CYS 166 R THR 167 R LEU 232 R ILE 233 R THR 244 R ALA 247 R PHE 343 R PHE 344 R SER 347 R LEU 370 |
| N1=C(SCCC1)Nc1c(OC)ccc(c1)C | 6.686 | R PHE 158 R ILE 159 R ASP 162 R VAL 163 R CYS 231 R LEU 232 R ILE 233 R PHE 343 R SER 347 R ARG 350 R LEU 363 R GLU 366 R ARG 367 R LEU 370 |
| N1=C(SCCC1)Nc1c(OC)cc(Cl)cc1 | 6.685 | R PHE 158 R ILE 159 R ASP 162 R VAL 163 R CYS 231 R LEU 232 R ILE 233 R PHE 343 R PHE 344 R SER 347 |
| N1=C(SCCC1)Nc1cc(O)ccc1F | 6.684 | R PHE 158 R ILE 159 R ASP 162 R VAL 163 R CYS 166 R LEU 232 R ILE 233 R THR 244 R ALA 247 R PHE 343 R PHE 344 R SER 347 |
| N1=C(SCCC1)Nc1c(F)cc(O)cc1 | 6.683 | R PHE 158 R ILE 159 R ASP 162 R VAL 163 R CYS 231 R LEU 232 R ILE 233 R PHE 343 R PHE 344 R SER 347 |
| N1=C(SCCC1)Nc1c(OC)cc(OC)cc1Cl | 6.681 | R ASP 162 R VAL 163 R THR 167 R ILE 213 R LEU 232 R ILE 233 R GLN 235 R SER 243 R THR 244 R ALA 247 R PHE 343 R PHE 344 R SER 347 R ARG 350 R GLU 366 R ARG 367 R LEU 370 |
| N1=C(SCCC1)[C@@H](N)c1cc(Cl)ccc1 | 6.679 | R ASP 162 R VAL 163 R CYS 166 R THR 167 R ILE 213 R ILE 233 R SER 234 R TYR 239 R THR 240 R SER 243 R THR 244 R ALA 247 R PHE 343 R PHE 344 R SER 347 |
| N1=C(SCCC1)NCc1ccccc1 | 6.675 | R ILE 159 R ASP 162 R VAL 163 R THR 167 R ILE 213 R LEU 232 R ILE 233 R SER 243 R THR 244 R ALA 247 R PHE 343 R PHE 344 R SER 347 |
| N1=C(SCCC1)Nc1c(F)cc(cc1)C | 6.669 | R PHE 158 R ILE 159 R ASP 162 R VAL 163 R CYS 166 R CYS 231 R LEU 232 R ILE 233 R SER 243 R THR 244 R PHE 343 R PHE 344 R ARG 367 R LEU 370 |
| N1=C(SCCC1)Nc1c(O)ccc(c1)C | 6.649 | R ASP 162 R VAL 163 R ILE 213 R LEU 232 R ILE 233 R GLN 235 R SER 243 R THR 244 R ALA 247 R PHE 343 R PHE 344 R SER 347 R ARG 350 |
| N1=C(SCCC1)N(c1c(Cl)cc(Cl)cc1)C | 6.649 | R PHE 158 R ILE 159 R ASP 162 R VAL 163 R CYS 166 R ILE 213 R LEU 232 R ILE 233 R SER 243 R ALA 247 R PHE 343 R PHE 344 R SER 347 |
| N1=C(SCCC1)N(C1CC1)C1=CCCCC1 | 6.637 | R ILE 159 R ASP 162 R VAL 163 R CYS 166 R THR 167 R ILE 213 R ILE 233 R SER 234 R TYR 239 R THR 240 R SER 243 R THR 244 R ALA 247 R PHE 343 R PHE 344 R SER 347 R THR 348 R LEU 370 |
| N1=C(SCCC1)[C@@H](N)c1cc(F)ccc1 | 6.634 | R ASP 162 R VAL 163 R CYS 166 R THR 167 R ILE 213 R ILE 233 R SER 234 R TYR 239 R THR 240 R SER 243 R THR 244 R ALA 247 R PHE 343 R PHE 344 R SER 347 |
| N1=C(SCCC1)Nc1c(C)cncc1C | 6.632 | R ILE 159 R ASP 162 R VAL 163 R CYS 166 R THR 167 R ILE 213 R LEU 232 R ILE 233 R SER 243 R THR 244 R ALA 247 R TRP 340 R PHE 343 R PHE 344 R SER 347 R LEU 370 |
| N1=C(SCCC1)Nc1c(ccnc1)C | 6.632 | R ILE 159 R ASP 162 R VAL 163 R CYS 166 R THR 167 R ILE 213 R LEU 232 R ILE 233 R SER 243 R THR 244 R ALA 247 R PHE 343 R PHE 344 R SER 347 R LEU 370 |
| N1=C(SCCC1)Nc1c(O)cc(O)cc1 | 6.629 | R PHE 158 R ILE 159 R ASP 162 R VAL 163 R CYS 166 R THR 167 R ILE 213 R LEU 232 R ILE 233 R SER 243 R ALA 247 R TRP 340 R PHE 343 R PHE 344 R SER 347 R LEU 370 |
| N1=C(SCCC1)Nc1c(OCC)cc(F)cc1 | 6.627 | R VAL 163 R CYS 166 R THR 167 R LEU 232 R ILE 233 R GLN 235 R TYR 239 R THR 240 R SER 243 R THR 244 R ALA 247 R PHE 343 R PHE 344 R SER 347 R ARG 350 R LEU 370 |
| N1=C(SCCC1)N[C@@H](C1CCC1)C(F)(F)F | 6.627 | R PHE 158 R ILE 159 R ASP 162 R VAL 163 R LEU 232 R ILE 233 R PHE 343 R PHE 344 R SER 347 R ARG 367 R LEU 370 |
| N1=C(SCCC1)Nc1ccc(F)c(F)c1 | 6.627 | R PHE 158 R ILE 159 R ASP 162 R VAL 163 R ILE 213 R ILE 233 R SER 243 R THR 244 R PHE 343 R PHE 344 R SER 347 R LEU 370 |
| N1=C(SCCC1)N[C@H]1[C@@H](CCCC1)CC | 6.625 | R PHE 158 R ILE 159 R ASP 162 R VAL 163 R CYS 166 R CYS 231 R LEU 232 R ILE 233 R PHE 343 R PHE 344 R SER 347 R ARG 367 R LEU 370 |
| N1=C(SCCC1)N[C@H]1[C@H](NC(=O)CC1)C | 6.624 | R PHE 158 R ILE 159 R ASP 162 R VAL 163 R CYS 166 R THR 167 R LEU 232 R ILE 233 R ALA 247 R TRP 340 R PHE 343 R PHE 344 R SER 347 R LEU 370 |
| N1=C(SCCC1)N(c1cc(ccc1)C)CC | 6.62 | R PHE 158 R ILE 159 R ASP 162 R VAL 163 R CYS 166 R THR 167 R ILE 213 R LEU 232 R ILE 233 R THR 240 R SER 243 R ALA 247 R TRP 340 R PHE 343 R PHE 344 R SER 347 |
| N1=C(SCCC1)Nc1c(cccc1N)C | 6.618 | R PHE 158 R ILE 159 R ASP 162 R VAL 163 R LEU 232 R ILE 233 R PHE 343 R SER 347 R ARG 350 R GLU 366 R ARG 367 R LEU 370 |
| N1=C(SCCC1)Nc1ccc(cc1)C | 6.618 | R ASP 162 R VAL 163 R ILE 213 R LEU 232 R ILE 233 R SER 243 R THR 244 R ALA 247 R PHE 343 R PHE 344 R SER 347 R ARG 350 R LEU 370 |
| N1=C(SCCC1)Nc1c(F)c(Cl)ccc1 | 6.615 | R VAL 138 R PHE 158 R ILE 159 R ASP 162 R VAL 163 R CYS 166 R CYS 231 R LEU 232 R ILE 233 R PHE 343 R PHE 344 R ARG 367 R LEU 370 |
| N1=C(SCCC1)Nc1cc(Cl)cc(Cl)c1 | 6.613 | R ASP 162 R VAL 163 R ILE 213 R LEU 232 R ILE 233 R SER 243 R THR 244 R ALA 247 R PHE 343 R PHE 344 R SER 347 R ARG 350 |
| N1=C(SCCC1)Nc1c(onc1C)CC | 6.61 | R ILE 159 R ASP 162 R VAL 163 R CYS 166 R THR 167 R ILE 213 R LEU 232 R ILE 233 R THR 240 R SER 243 R THR 244 R ALA 247 R PHE 343 R PHE 344 R SER 347 R LEU 370 |
| N1=C(SCCC1)Nc1ccc(Br)cc1F | 6.61 | R PHE 158 R ILE 159 R ASP 162 R VAL 163 R CYS 231 R LEU 232 R ILE 233 R SER 243 R PHE 343 R PHE 344 R ARG 367 R LEU 370 |
| N1=C(SCCC1)Nc1c(cc(O)cc1)C | 6.609 | R ASP 162 R VAL 163 R CYS 166 R THR 167 R ILE 213 R LEU 232 R ILE 233 R SER 243 R THR 244 R ALA 247 R PHE 343 R PHE 344 R SER 347 R LEU 370 |
| N1=C(SCCC1)N[C@@H]1[C@H](CCCC1)C | 6.606 | R PHE 158 R ILE 159 R ASP 162 R VAL 163 R CYS 166 R THR 167 R LEU 232 R ILE 233 R TRP 340 R PHE 343 R PHE 344 R SER 347 R LEU 370 |
| N1=C(SCCC1)Nc1cc(F)c(OC)cc1 | 6.602 | R ASP 162 R VAL 163 R ILE 213 R LEU 232 R ILE 233 R SER 243 R THR 244 R ALA 247 R PHE 343 R PHE 344 R SER 347 R ARG 350 R ARG 367 R LEU 370 |
| N1=C(SCCC1)Cc1c(cccc1F)C | 6.599 | R ASP 162 R VAL 163 R CYS 166 R THR 167 R ILE 213 R ILE 233 R SER 234 R TYR 239 R THR 240 R SER 243 R THR 244 R ALA 247 R TRP 340 R PHE 343 R PHE 344 R SER 347 |
| N1=C(SCCC1)Nc1c(Cl)cc(OC)cc1 | 6.594 | R VAL 138 R PHE 158 R ILE 159 R ASP 162 R VAL 163 R CYS 166 R THR 167 R ILE 213 R CYS 231 R LEU 232 R ILE 233 R SER 243 R THR 244 R ALA 247 R TRP 340 R PHE 343 R PHE 344 R ARG 367 R LEU 370 |
| N1=C(SCCC1)Nc1c(OCC)ccc(Cl)c1 | 6.594 | R PHE 158 R ILE 159 R ASP 162 R VAL 163 R ILE 213 R LEU 232 R ILE 233 R THR 240 R SER 243 R THR 244 R ALA 247 R PHE 343 R PHE 344 R SER 347 R ARG 350 |
| N1=C(SCCC1)Nc1c(Br)cc(N)cc1 | 6.59 | R ASP 162 R VAL 163 R CYS 166 R THR 167 R ILE 213 R LEU 232 R ILE 233 R ALA 247 R PHE 343 R PHE 344 R SER 347 R LEU 370 R TYR 374 |
| N1=C(SCCC1)Nc1c(OC)c(Cl)ccc1 | 6.586 | R PHE 158 R ILE 159 R ASP 162 R VAL 163 R CYS 166 R THR 167 R LEU 232 R ILE 233 R THR 244 R ALA 247 R TRP 340 R PHE 343 R PHE 344 R SER 347 R LEU 370 R TYR 374 |
| N1=C(SCCC1)Nc1c(N)ccc(F)c1 | 6.584 | R ASP 162 R VAL 163 R ILE 213 R LEU 232 R ILE 233 R GLN 235 R SER 243 R THR 244 R ALA 247 R PHE 343 R PHE 344 R SER 347 R ARG 350 |
| N1=C(SCCC1)Nc1c(Cl)cccc1 | 6.578 | R PHE 158 R ILE 159 R ASP 162 R VAL 163 R CYS 231 R LEU 232 R ILE 233 R PHE 343 R PHE 344 R SER 347 |
| N1=C(SCCC1)Nc1c(OC)c(OC)ccc1 | 6.575 | R PHE 158 R ILE 159 R ASP 162 R VAL 163 R CYS 166 R THR 167 R ILE 213 R LEU 232 R ILE 233 R THR 244 R ALA 247 R TRP 340 R PHE 343 R PHE 344 R SER 347 R LEU 370 R TYR 374 |
| N1=C(SCCC1)Nc1c(N)c(F)ccc1 | 6.573 | R PHE 158 R ILE 159 R ASP 162 R VAL 163 R LEU 232 R ILE 233 R PHE 343 R ARG 350 R LEU 363 R GLU 366 R ARG 367 R LEU 370 |
| N1=C(SCCC1)Cc1c(cccc1C)C | 6.566 | R PHE 158 R ILE 159 R ASP 162 R VAL 163 R CYS 166 R THR 167 R ILE 213 R LEU 232 R ILE 233 R SER 243 R ALA 247 R TRP 340 R PHE 343 R PHE 344 R SER 347 R LEU 370 |
| N1=C(SCCC1)Nc1c(F)cc(Cl)cc1 | 6.562 | R PHE 158 R ILE 159 R ASP 162 R VAL 163 R CYS 231 R LEU 232 R ILE 233 R SER 243 R PHE 343 R PHE 344 R ARG 367 R LEU 370 |
| N1=C(SCCC1)Nc1ccc(nc1O)C | 6.562 | R PHE 158 R ILE 159 R ASP 162 R VAL 163 R CYS 166 R THR 167 R ILE 213 R LEU 232 R ILE 233 R SER 243 R ALA 247 R PHE 343 R PHE 344 R SER 347 |
| N1=C(SCCC1)Nc1c(COC)cccc1 | 6.559 | R PHE 158 R ILE 159 R ASP 162 R VAL 163 R CYS 231 R LEU 232 R ILE 233 R GLN 235 R PHE 343 R PHE 344 R SER 347 R ARG 350 R LEU 370 |
| N1=C(SCCC1)Nc1c(N)c(OC)ccc1 | 6.559 | R PHE 158 R ASP 162 R VAL 163 R ILE 213 R LEU 232 R ILE 233 R SER 243 R THR 244 R ALA 247 R PHE 343 R PHE 344 R SER 347 R ARG 350 R LEU 370 |
| N1=C(SCCC1)Nc1c(N)ccc(Cl)c1 | 6.555 | R ASP 162 R VAL 163 R THR 167 R ILE 213 R LEU 232 R ILE 233 R SER 243 R THR 244 R ALA 247 R PHE 343 R PHE 344 R SER 347 R ARG 350 |
| N1=C(SCCC1)N(CC)c1cc(Cl)ccc1 | 6.55 | R ILE 159 R ASP 162 R VAL 163 R CYS 166 R THR 167 R LEU 232 R ILE 233 R SER 234 R TYR 239 R THR 240 R SER 243 R THR 244 R PHE 343 R PHE 344 R SER 347 |
| N1=C(SCCC1)Nc1cc(NC)ccc1 | 6.545 | R PHE 158 R ILE 159 R ASP 162 R VAL 163 R LEU 232 R ILE 233 R PHE 343 R SER 347 R ARG 350 R GLU 366 R ARG 367 R LEU 370 |
| N1=C(SCCC1)Nc1c(Cl)cc(N)cc1 | 6.543 | R ASP 162 R VAL 163 R CYS 166 R THR 167 R ILE 213 R LEU 232 R ILE 233 R ALA 247 R PHE 343 R PHE 344 R SER 347 R LEU 370 R TYR 374 |
| N1=C(SCCC1)Nc1cc(Br)cnc1O | 6.542 | R PHE 158 R ILE 159 R ASP 162 R VAL 163 R LEU 232 R ILE 233 R SER 243 R THR 244 R ALA 247 R PHE 343 R PHE 344 R SER 347 |
| N1=C(SCCC1)Nc1c(OC)cc(N)cc1 | 6.539 | R ASP 162 R VAL 163 R CYS 166 R THR 167 R ILE 213 R LEU 232 R ILE 233 R SER 243 R THR 244 R ALA 247 R PHE 343 R PHE 344 R SER 347 R LEU 370 R TYR 374 |
| N1=C(SCCC1)N[C@H](C1CC1)c1sccc1 | 6.538 | R PHE 158 R ILE 159 R ASP 162 R VAL 163 R CYS 166 R LEU 232 R ILE 233 R PHE 343 R PHE 344 R SER 347 R ARG 350 R GLU 366 R LEU 370 |
| N1=C(SCCC1)Nc1ccc(F)cc1Cl | 6.535 | R VAL 138 R PHE 158 R ILE 159 R ASP 162 R VAL 163 R CYS 166 R CYS 231 R LEU 232 R ILE 233 R SER 243 R TRP 340 R PHE 343 R PHE 344 R ARG 367 R LEU 370 |
| N1=C(SCCC1)Nc1c(N)c(Cl)ccc1 | 6.532 | R ASP 162 R VAL 163 R ILE 213 R LEU 232 R ILE 233 R SER 243 R THR 244 R ALA 247 R PHE 343 R PHE 344 R SER 347 R ARG 350 |
| N1=C(SCCC1)Nc1c(Cl)c(F)ccc1 | 6.532 | R VAL 138 R PHE 158 R ILE 159 R ASP 162 R VAL 163 R CYS 166 R CYS 231 R LEU 232 R ILE 233 R PHE 343 R PHE 344 R ARG 367 R LEU 370 |
| N1=C(SCCC1)N[C@@H](C1CCC1)C1CC1 | 6.529 | R PHE 158 R ILE 159 R ASP 162 R VAL 163 R CYS 166 R THR 167 R LEU 232 R ILE 233 R TRP 340 R PHE 343 R PHE 344 R SER 347 R ARG 350 R LEU 370 |
| N1=C(SCCC1)Nc1c(OCC)nccc1 | 6.526 | R ILE 159 R ASP 162 R VAL 163 R THR 167 R ILE 213 R LEU 232 R ILE 233 R SER 234 R TYR 239 R THR 240 R SER 243 R THR 244 R ALA 247 R PHE 343 R PHE 344 R SER 347 R LEU 370 |
| N1=C(SCCC1)Nc1c(Cl)c(Cl)ccc1 | 6.523 | R VAL 138 R PHE 158 R ILE 159 R ASP 162 R VAL 163 R CYS 166 R CYS 231 R LEU 232 R ILE 233 R PHE 343 R PHE 344 R ARG 367 R LEU 370 |
| N1=C(SCCC1)Nc1ccc(F)cc1 | 6.514 | R ASP 162 R VAL 163 R ILE 213 R LEU 232 R ILE 233 R SER 243 R THR 244 R ALA 247 R PHE 343 R PHE 344 R SER 347 R ARG 350 R LEU 370 |
| N1=C(SCCC1)N(c1c(CN)cccc1)C | 6.512 | R ASP 162 R VAL 163 R CYS 166 R THR 167 R ILE 213 R ILE 233 R SER 234 R TYR 239 R THR 240 R SER 243 R THR 244 R ALA 247 R TRP 340 R PHE 343 R PHE 344 R SER 347 |
| N1=C(SCCC1)CN1[C@H](CCCCC1)C | 6.51 | R ASP 162 R VAL 163 R CYS 166 R THR 167 R ILE 213 R LEU 232 R ILE 233 R GLN 235 R SER 243 R THR 244 R ALA 247 R TRP 340 R PHE 343 R PHE 344 R SER 347 R ARG 350 R LEU 370 |
| N1=C(SCCC1)N[C@@H](c1c(n(nc1)C)C)C | 6.509 | R PHE 158 R ILE 159 R ASP 162 R VAL 163 R CYS 166 R THR 167 R ILE 213 R LEU 232 R ILE 233 R SER 243 R THR 244 R ALA 247 R TRP 340 R PHE 343 R PHE 344 R SER 347 R LEU 370 |
| N1=C(SCCC1)Nc1c(N)cccc1Cl | 6.506 | R PHE 158 R ILE 159 R ASP 162 R VAL 163 R LEU 232 R ILE 233 R PHE 343 R ARG 350 R GLU 366 R ARG 367 R LEU 370 |
| N1=C(SCCC1)Nc1c(F)cc(N)cc1 | 6.506 | R ASP 162 R VAL 163 R CYS 166 R THR 167 R ILE 213 R LEU 232 R ILE 233 R ALA 247 R PHE 343 R PHE 344 R SER 347 R LEU 370 R TYR 374 |
| N1=C(SCCC1)Nc1cc(F)ccc1 | 6.503 | R ASP 162 R VAL 163 R ILE 213 R LEU 232 R ILE 233 R SER 243 R THR 244 R ALA 247 R PHE 343 R PHE 344 R SER 347 R ARG 350 |
| N1=C(SCCC1)Nc1c(N)cc(Cl)cc1 | 6.501 | R ASP 162 R VAL 163 R ILE 213 R LEU 232 R ILE 233 R SER 243 R THR 244 R ALA 247 R PHE 343 R PHE 344 R SER 347 R ARG 350 R LEU 370 |
| N1=C(SCCC1)Nc1c(cncc1)C | 6.494 | R ASP 162 R VAL 163 R CYS 166 R THR 167 R ILE 213 R ILE 233 R SER 234 R THR 240 R SER 243 R THR 244 R ALA 247 R TRP 340 R PHE 343 R PHE 344 R SER 347 |
| N1=C(SCCC1)N(c1c(F)cccc1)C | 6.494 | R ASP 162 R VAL 163 R CYS 166 R THR 167 R ILE 213 R ILE 233 R SER 234 R TYR 239 R THR 240 R SER 243 R THR 244 R ALA 247 R TRP 340 R PHE 343 R PHE 344 R SER 347 |
| N1=C(SCCC1)Nc1ncc(Cl)cc1N | 6.491 | R ASP 162 R VAL 163 R ILE 213 R LEU 232 R ILE 233 R SER 243 R THR 244 R ALA 247 R PHE 343 R PHE 344 R SER 347 R ARG 350 |
| N1=C(SCCC1)Nc1c(CN)cccc1 | 6.487 | R PHE 158 R ILE 159 R ASP 162 R VAL 163 R CYS 166 R THR 167 R ILE 213 R LEU 232 R ILE 233 R SER 243 R THR 244 R ALA 247 R TRP 340 R PHE 343 R PHE 344 R SER 347 |
| N1=C(SCCC1)N[C@@H](C(C)C)c1sccc1 | 6.484 | R PHE 158 R ILE 159 R ASP 162 R VAL 163 R CYS 166 R THR 167 R LEU 232 R ILE 233 R TRP 340 R PHE 343 R PHE 344 R SER 347 R ARG 350 R LEU 370 |
| N1=C(SCCC1)Nc1c(N)ccc(OC)c1 | 6.479 | R ASP 162 R VAL 163 R ILE 213 R LEU 232 R ILE 233 R SER 243 R THR 244 R ALA 247 R PHE 343 R PHE 344 R SER 347 R ARG 350 R GLU 366 |
| N1=C(SCCC1)Nc1c(OC)ccc(F)c1 | 6.478 | R ASP 162 R VAL 163 R ILE 213 R LEU 232 R ILE 233 R GLN 235 R SER 243 R THR 244 R ALA 247 R PHE 343 R PHE 344 R SER 347 R ARG 350 R LEU 370 |
| N1=C(SCCC1)Nc1c([nH+]ccc1)N | 6.469 | R ASP 162 R VAL 163 R CYS 166 R THR 167 R ILE 233 R SER 234 R THR 240 R SER 243 R THR 244 R ALA 247 R TRP 340 R PHE 343 R PHE 344 R SER 347 R LEU 370 |
| N1=C(SCCC1)Cc1c(cccc1Cl)C | 6.467 | R PHE 158 R ILE 159 R ASP 162 R VAL 163 R CYS 166 R THR 167 R LEU 232 R ILE 233 R SER 243 R ALA 247 R PHE 343 R PHE 344 R SER 347 R LEU 370 |
| N1=C(SCCC1)Cc1ncccc1C | 6.466 | R ASP 162 R VAL 163 R CYS 166 R THR 167 R ILE 213 R ILE 233 R SER 234 R TYR 239 R THR 240 R SER 243 R THR 244 R ALA 247 R PHE 343 R PHE 344 R SER 347 |
| N1=C(SCCC1)Nc1c(SCC)cccc1 | 6.465 | R PHE 158 R ILE 159 R ASP 162 R VAL 163 R CYS 231 R LEU 232 R ILE 233 R PHE 343 R PHE 344 R SER 347 R ARG 350 R GLU 366 R LEU 370 |
| N1=C(SCCC1)Nc1ccc(OC)nc1OC | 6.455 | R PHE 158 R ILE 159 R ASP 162 R VAL 163 R CYS 231 R LEU 232 R ILE 233 R PHE 343 R PHE 344 R SER 347 R ARG 367 |
| N1=C(SCCC1)Oc1c(CN)c(Cl)ccc1 | 6.45 | R PHE 158 R ILE 159 R ASP 162 R VAL 163 R CYS 166 R THR 167 R ILE 213 R LEU 232 R ILE 233 R TYR 239 R SER 243 R ALA 247 R TRP 340 R PHE 343 R PHE 344 R SER 347 |
| N1=C(SCCC1)N(c1c(C)cccc1)CC | 6.441 | R PHE 158 R ILE 159 R ASP 162 R VAL 163 R CYS 166 R LEU 232 R ILE 233 R TYR 239 R THR 240 R SER 243 R THR 244 R PHE 343 R PHE 344 R SER 347 R LEU 370 |
| N1=C(SCCC1)Nc1c(N)cc(Br)cc1 | 6.438 | R ASP 162 R VAL 163 R THR 167 R ILE 213 R LEU 232 R ILE 233 R SER 243 R THR 244 R ALA 247 R PHE 343 R PHE 344 R SER 347 R ARG 350 R LEU 370 |
| N1=C(SCCC1)[C@H]1CCCc2sccc12 | 6.438 | R PHE 158 R ILE 159 R ASP 162 R VAL 163 R CYS 166 R THR 167 R LEU 232 R ILE 233 R SER 243 R THR 244 R TRP 340 R PHE 343 R PHE 344 R SER 347 R LEU 370 |
| N1=C(SCCC1)Nc1c(N)cccc1F | 6.432 | R PHE 158 R ILE 159 R ASP 162 R VAL 163 R LEU 232 R ILE 233 R PHE 343 R ARG 350 R GLU 366 R ARG 367 R LEU 370 |
| N1=C(SCCC1)Nc1c(O)ccc(Cl)c1 | 6.428 | R ASP 162 R VAL 163 R ILE 213 R LEU 232 R ILE 233 R SER 243 R THR 244 R ALA 247 R PHE 343 R PHE 344 R SER 347 R ARG 350 |
| N1=C(SCCC1)N[C@H](C)c1nccnc1 | 6.421 | R ASP 162 R VAL 163 R CYS 166 R THR 167 R ILE 213 R ILE 233 R TYR 239 R THR 240 R SER 243 R THR 244 R ALA 247 R TRP 340 R PHE 343 R PHE 344 R SER 347 R THR 348 R LEU 370 |
| N1=C(SCCC1)Nc1cc(N)ccc1Cl | 6.42 | R ASP 162 R VAL 163 R ILE 213 R LEU 232 R ILE 233 R THR 240 R SER 243 R THR 244 R ALA 247 R PHE 343 R PHE 344 R SER 347 R ARG 350 |
| N1=C(SCCC1)Nc1cc(Cl)cnc1O | 6.419 | R PHE 158 R ILE 159 R ASP 162 R VAL 163 R CYS 166 R THR 167 R LEU 232 R ILE 233 R SER 243 R THR 244 R ALA 247 R PHE 343 R PHE 344 R SER 347 |
| N1=C(SCCC1)Nc1ccc(Br)cc1 | 6.413 | R ASP 162 R VAL 163 R ILE 213 R LEU 232 R ILE 233 R SER 243 R THR 244 R ALA 247 R PHE 343 R PHE 344 R SER 347 R ARG 350 R LEU 370 |
| N1=C(SCCC1)Nc1c(N)cccc1 | 6.412 | R ASP 162 R VAL 163 R ILE 213 R LEU 232 R ILE 233 R SER 243 R THR 244 R ALA 247 R PHE 343 R PHE 344 R SER 347 R ARG 350 |
| N1=C(SCCC1)Oc1c(cccc1C)C | 6.412 | R ILE 159 R ASP 162 R VAL 163 R CYS 166 R THR 167 R ILE 213 R ILE 233 R TYR 239 R THR 240 R SER 243 R THR 244 R ALA 247 R TRP 340 R PHE 343 R PHE 344 R SER 347 R LEU 370 |
| N1=C(SCCC1)Nc1c(n(nc1C)CC)C | 6.408 | R ILE 159 R ASP 162 R VAL 163 R ILE 213 R LEU 232 R ILE 233 R GLN 235 R SER 243 R THR 244 R ALA 247 R PHE 343 R PHE 344 R SER 347 R ARG 350 R LEU 370 |
| N1=C(SCCC1)Nc1cc(N)ccc1 | 6.408 | R ASP 162 R VAL 163 R ILE 213 R LEU 232 R ILE 233 R GLN 235 R SER 243 R THR 244 R ALA 247 R PHE 343 R PHE 344 R SER 347 R ARG 350 |
| N1=C(SCCC1)Nc1cc(N)ccc1Br | 6.407 | R PHE 158 R ILE 159 R ASP 162 R VAL 163 R LEU 232 R ILE 233 R PHE 343 R SER 347 R ARG 350 R GLU 366 R ARG 367 R LEU 370 |
| N1=C(SCCC1)N[C@@H]1[C@@H](F)CCCC1 | 6.396 | R PHE 158 R ILE 159 R ASP 162 R VAL 163 R CYS 166 R THR 167 R CYS 231 R LEU 232 R ILE 233 R PHE 343 R PHE 344 R SER 347 R ARG 367 R LEU 370 |
| N1=C(SCCC1)Nc1c(N(C)C)c(Cl)ccc1 | 6.392 | R ASP 162 R VAL 163 R CYS 166 R THR 167 R ILE 213 R LEU 232 R ILE 233 R TYR 239 R SER 243 R THR 244 R ALA 247 R PHE 343 R PHE 344 R SER 347 R LEU 370 R TYR 374 |
| N1=C(SCCC1)Nc1c(N)cc(OC)cc1 | 6.392 | R PHE 158 R ASP 162 R VAL 163 R CYS 166 R THR 167 R ILE 213 R LEU 232 R ILE 233 R SER 243 R THR 244 R ALA 247 R PHE 343 R PHE 344 R SER 347 R ARG 367 R LEU 370 |
| N1=C(SCCC1)C[n+]1c(cc(cc1C)C)C | 6.39 | R ILE 159 R ASP 162 R VAL 163 R CYS 166 R THR 167 R ILE 213 R ILE 233 R TYR 239 R THR 240 R SER 243 R THR 244 R ALA 247 R TRP 340 R PHE 343 R PHE 344 R SER 347 R LEU 370 R TYR 374 |
| N1=C(SCCC1)N(c1c(C)cccc1)C | 6.389 | R ASP 162 R VAL 163 R CYS 166 R THR 167 R ILE 213 R ILE 233 R SER 234 R TYR 239 R THR 240 R SER 243 R THR 244 R ALA 247 R TRP 340 R PHE 343 R PHE 344 R SER 347 |
| N1=C(SCCC1)Nc1c([nH]nc1C(F)F)C | 6.385 | R PHE 158 R ILE 159 R ASP 162 R CYS 231 R LEU 232 R ILE 233 R GLN 235 R PHE 343 R SER 347 R ARG 350 R GLU 366 R ARG 367 R LEU 370 |
| N1=C(SCCC1)Nc1c(OC)cccc1 | 6.382 | R ASP 162 R VAL 163 R THR 167 R ILE 213 R LEU 232 R ILE 233 R THR 240 R SER 243 R THR 244 R ALA 247 R PHE 343 R PHE 344 R SER 347 R LEU 370 |
| N1=C(SCCC1)Nc1c([nH]nc1)CC | 6.374 | R ASP 162 R VAL 163 R CYS 166 R THR 167 R ILE 213 R ILE 233 R GLN 235 R THR 240 R SER 243 R THR 244 R ALA 247 R TRP 340 R PHE 343 R PHE 344 R SER 347 |
| N1=C(SCCC1)Nc1c(SC)cccc1 | 6.369 | R PHE 158 R ILE 159 R ASP 162 R VAL 163 R CYS 231 R LEU 232 R ILE 233 R PHE 343 R PHE 344 R SER 347 R LEU 370 |
| N1=C(SCCC1)N1C[C@H]2[C@H](CCC2)C1 | 6.367 | R PHE 158 R ILE 159 R ASP 162 R VAL 163 R THR 167 R ILE 213 R LEU 232 R ILE 233 R THR 240 R SER 243 R THR 244 R ALA 247 R PHE 343 R PHE 344 R SER 347 R LEU 370 |
| N1=C(SCCC1)Nc1c(O)cc(Cl)cc1 | 6.358 | R ASP 162 R VAL 163 R ILE 213 R LEU 232 R ILE 233 R SER 243 R THR 244 R ALA 247 R PHE 343 R PHE 344 R SER 347 R ARG 350 R LEU 370 |
| N1=C(SCCC1)N(c1c(CC)cccc1)C | 6.355 | R ILE 159 R ASP 162 R VAL 163 R CYS 166 R THR 167 R ILE 213 R LEU 232 R ILE 233 R TYR 239 R SER 243 R ALA 247 R TRP 340 R PHE 343 R PHE 344 R SER 347 R ARG 350 |
| N1=C(SCCC1)Oc1c(CNC)cccc1 | 6.348 | R PHE 158 R ILE 159 R ASP 162 R VAL 163 R CYS 166 R THR 167 R LEU 232 R ILE 233 R THR 240 R SER 243 R THR 244 R PHE 343 R PHE 344 R SER 347 R LEU 370 |
| N1=C(SCCC1)Nc1c(nsc1)c1ccccc1 | 6.33 | R ILE 159 R ASP 162 R VAL 163 R CYS 166 R THR 167 R ILE 213 R ILE 233 R THR 240 R SER 243 R THR 244 R ALA 247 R TRP 340 R PHE 343 R PHE 344 R SER 347 |
| N1=C(SCCC1)N[C@H]1CCNC1(C)C | 6.326 | R PHE 158 R ILE 159 R ASP 162 R VAL 163 R CYS 166 R LEU 232 R ILE 233 R PHE 343 R PHE 344 R SER 347 R LEU 370 R TYR 374 |
| N1=C(SCCC1)Nc1c(nn(c1C)C)C | 6.325 | R ASP 162 R VAL 163 R CYS 166 R THR 167 R ILE 213 R LEU 232 R ILE 233 R GLN 235 R THR 240 R SER 243 R THR 244 R ALA 247 R PHE 343 R PHE 344 R SER 347 R ARG 350 R LEU 370 |
| N1=C(SCCC1)NCc1c[nH]nc1C | 6.324 | R ASP 162 R VAL 163 R CYS 166 R THR 167 R ILE 213 R ILE 233 R SER 234 R TYR 239 R THR 240 R SER 243 R THR 244 R ALA 247 R TRP 340 R PHE 343 R PHE 344 R SER 347 R LEU 370 |
| N1=C(SCCC1)Nc1cccnc1O | 6.317 | R ASP 162 R VAL 163 R CYS 166 R THR 167 R ILE 213 R ILE 233 R SER 234 R THR 240 R SER 243 R THR 244 R ALA 247 R PHE 343 R PHE 344 R SER 347 |
| N1=C(SCCC1)NC1(OCCO1)C(F)F | 6.314 | R ASP 162 R VAL 163 R CYS 166 R THR 167 R ILE 213 R ILE 233 R TYR 239 R THR 240 R SER 243 R THR 244 R ALA 247 R TRP 340 R PHE 343 R PHE 344 R SER 347 |
| N1=C(SCCC1)N[C@@H]1[C@@H](OC)CCCC1 | 6.311 | R PHE 158 R ILE 159 R ASP 162 R VAL 163 R CYS 166 R THR 167 R LEU 232 R ILE 233 R TRP 340 R PHE 343 R PHE 344 R SER 347 R LEU 370 |
| N1=C(SCCC1)N[C@@H]1C[C@@H](O[C@H]1C)C | 6.305 | R VAL 163 R ILE 213 R LEU 232 R ILE 233 R SER 234 R GLN 235 R TYR 239 R THR 240 R SER 243 R THR 244 R ALA 247 R PHE 343 R PHE 344 R SER 347 R ARG 350 R LEU 370 |
| N1=C(SCCC1)Nc1c(S)cccc1 | 6.304 | R PHE 158 R ILE 159 R ASP 162 R VAL 163 R CYS 231 R LEU 232 R ILE 233 R PHE 343 R PHE 344 R SER 347 R LEU 370 |
| N1=C(SCCC1)Oc1c(CN)cccc1C | 6.298 | R PHE 158 R ILE 159 R ASP 162 R VAL 163 R CYS 166 R THR 167 R ILE 213 R LEU 232 R ILE 233 R SER 243 R ALA 247 R TRP 340 R PHE 343 R PHE 344 R SER 347 R LEU 370 |
| N1=C(SCCC1)N[C@H]1[C@@H](CCN[C@@H]1C)C | 6.297 | R PHE 158 R ILE 159 R ASP 162 R VAL 163 R CYS 166 R THR 167 R ILE 213 R LEU 232 R ILE 233 R TYR 239 R SER 243 R ALA 247 R TRP 340 R PHE 343 R PHE 344 R SER 347 |
| N1=C(SCCC1)Nc1c(nccc1)C | 6.292 | R PHE 158 R ILE 159 R ASP 162 R VAL 163 R CYS 231 R LEU 232 R ILE 233 R PHE 343 R PHE 344 R SER 347 R LEU 370 |
| N1=C(SCCC1)N[C@@H]1C(CCSC1)(C)C | 6.29 | R PHE 158 R ILE 159 R ASP 162 R VAL 163 R CYS 166 R THR 167 R ILE 213 R LEU 232 R ILE 233 R SER 243 R ALA 247 R PHE 343 R PHE 344 R SER 347 |
| N1=C(SCCC1)N[C@@H](C1CC1)C(F)(F)F | 6.284 | R PHE 158 R ILE 159 R ASP 162 R VAL 163 R LEU 232 R ILE 233 R PHE 343 R PHE 344 R SER 347 R ARG 367 R LEU 370 |
| N1=C(SCCC1)Oc1c([C@@H](N)C)cccc1 | 6.283 | R PHE 158 R ILE 159 R ASP 162 R VAL 163 R CYS 166 R LEU 232 R ILE 233 R TRP 340 R PHE 343 R PHE 344 R SER 347 R LEU 370 R TYR 374 |
| N1=C(SCCC1)Oc1c(cccc1Cl)C | 6.279 | R ASP 162 R VAL 163 R CYS 166 R THR 167 R ILE 213 R ILE 233 R TYR 239 R THR 240 R SER 243 R THR 244 R ALA 247 R TRP 340 R PHE 343 R PHE 344 R SER 347 R LEU 370 |
| N1=C(SCCC1)Nc1ccc(OC)cc1 | 6.277 | R PHE 158 R ASP 162 R VAL 163 R ILE 213 R LEU 232 R ILE 233 R SER 243 R THR 244 R ALA 247 R PHE 343 R PHE 344 R SER 347 R ARG 350 R ARG 367 R LEU 370 |
| N1=C(SCCC1)N[C@@H]([C@@H]1CCCOC1)C | 6.272 | R PHE 158 R ILE 159 R ASP 162 R VAL 163 R CYS 166 R THR 167 R LEU 232 R ILE 233 R SER 243 R ALA 247 R TRP 340 R PHE 343 R PHE 344 R SER 347 R LEU 370 |
| N1=C(SCCC1)N[C@@H]1[C@H](CCCC1)CN | 6.269 | R PHE 158 R ILE 159 R ASP 162 R VAL 163 R CYS 166 R LEU 232 R ILE 233 R PHE 343 R PHE 344 R SER 347 R ARG 367 R LEU 370 |
| N1=C(SCCC1)Oc1c(cccc1F)C | 6.263 | R PHE 158 R ILE 159 R ASP 162 R VAL 163 R CYS 166 R THR 167 R LEU 232 R ILE 233 R SER 243 R ALA 247 R PHE 343 R PHE 344 R SER 347 R LEU 370 |
| N1=C(SCCC1)Nc1c(Br)cccc1 | 6.262 | R PHE 158 R ILE 159 R ASP 162 R VAL 163 R LEU 232 R ILE 233 R PHE 343 R ARG 350 R GLU 366 R ARG 367 R LEU 370 |
| N1=C(SCCC1)N[C@H]([C@H](CC)C)C(=O)OC | 6.255 | R PHE 158 R ILE 159 R ASP 162 R VAL 163 R CYS 166 R CYS 231 R LEU 232 R ILE 233 R PHE 343 R PHE 344 R SER 347 R ARG 350 R LEU 370 |
| N1=C(SCCC1)Nc1c(n(nc1)C)C | 6.237 | R ILE 159 R ASP 162 R VAL 163 R THR 167 R ILE 213 R LEU 232 R ILE 233 R THR 240 R SER 243 R THR 244 R ALA 247 R PHE 343 R PHE 344 R SER 347 R LEU 370 |
| N1=C(SCCC1)C[n+]1c(cccc1)C | 6.235 | R ASP 162 R VAL 163 R CYS 166 R THR 167 R ILE 213 R ILE 233 R SER 234 R TYR 239 R THR 240 R SER 243 R THR 244 R ALA 247 R PHE 343 R PHE 344 R SER 347 |
| N1=C(SCCC1)N(CC)c1c(N)cccc1 | 6.233 | R PHE 158 R ILE 159 R ASP 162 R VAL 163 R CYS 166 R LEU 232 R ILE 233 R TYR 239 R THR 240 R SER 243 R THR 244 R PHE 343 R PHE 344 R SER 347 R LEU 370 |
| N1=C(SCCC1)N[C@H]1C(CN(C1)C)(C)C | 6.229 | R PHE 158 R ILE 159 R ASP 162 R VAL 163 R CYS 166 R THR 167 R CYS 231 R LEU 232 R ILE 233 R PHE 343 R PHE 344 R SER 347 R LEU 370 |
| N1=C(SCCC1)Cc1[n+](cccc1)C | 6.226 | R ASP 162 R VAL 163 R CYS 166 R THR 167 R ILE 213 R ILE 233 R SER 234 R TYR 239 R THR 240 R SER 243 R THR 244 R ALA 247 R PHE 343 R PHE 344 R SER 347 |
| N1=C(SCCC1)Nc1n(nc(c1N)C)CC | 6.222 | R PHE 158 R ILE 159 R ASP 162 R VAL 163 R LEU 232 R ILE 233 R GLN 235 R PHE 343 R SER 347 R ARG 350 R GLU 366 R ARG 367 R LEU 370 |
| N1=C(SCCC1)N(CC)c1c(F)cccc1 | 6.219 | R ILE 159 R ASP 162 R VAL 163 R CYS 166 R THR 167 R ILE 213 R LEU 232 R ILE 233 R TYR 239 R THR 240 R SER 243 R ALA 247 R TRP 340 R PHE 343 R PHE 344 R SER 347 |
| N1=C(SCCC1)Cc1ccncc1Br | 6.214 | R ASP 162 R VAL 163 R CYS 166 R THR 167 R ILE 213 R ILE 233 R SER 234 R TYR 239 R THR 240 R SER 243 R THR 244 R ALA 247 R PHE 343 R PHE 344 R SER 347 |
| N1=C(SCCC1)N[C@H]1[C@@H](CCCC1)CO | 6.208 | R PHE 158 R ILE 159 R ASP 162 R VAL 163 R CYS 166 R THR 167 R ILE 213 R LEU 232 R ILE 233 R SER 243 R THR 244 R ALA 247 R TRP 340 R PHE 343 R PHE 344 R SER 347 R LEU 370 |
| N1=C(SCCC1)N[C@H](CC)c1scc(n1)C | 6.208 | R PHE 158 R ILE 159 R ASP 162 R VAL 163 R CYS 166 R THR 167 R ILE 213 R LEU 232 R ILE 233 R SER 243 R THR 244 R ALA 247 R TRP 340 R PHE 343 R PHE 344 R SER 347 R LEU 370 R TYR 374 |
| N1=C(SCCC1)Nc1c(F)cccc1 | 6.202 | R PHE 158 R ILE 159 R ASP 162 R VAL 163 R LEU 232 R ILE 233 R PHE 343 R ARG 350 R GLU 366 R ARG 367 R LEU 370 |
| N1=C(SCCC1)N[C@@H]([C@H]1CCOC1)C | 6.197 | R ASP 162 R VAL 163 R CYS 166 R THR 167 R ILE 233 R SER 234 R TYR 239 R THR 240 R SER 243 R THR 244 R ALA 247 R TRP 340 R PHE 343 R PHE 344 R SER 347 R LEU 370 |
| N1=C(SCCC1)NC[C@@H]1N(CCC1)CCC | 6.197 | R PHE 158 R ILE 159 R ASP 162 R VAL 163 R CYS 166 R THR 167 R ILE 213 R CYS 231 R LEU 232 R ILE 233 R TYR 239 R SER 243 R THR 244 R ALA 247 R TRP 340 R PHE 343 R PHE 344 R SER 347 R LEU 370 |
| N1=C(SCCC1)N1[C@@H]([C@H](S(=O)(=O)CC1)C)C | 6.173 | R ILE 159 R ASP 162 R VAL 163 R CYS 166 R THR 167 R ILE 213 R LEU 232 R ILE 233 R THR 240 R SER 243 R THR 244 R ALA 247 R TRP 340 R PHE 343 R PHE 344 R SER 347 R LEU 370 |
| N1=C(SCCC1)N[C@@H](C1CCCC1)CN | 6.171 | R PHE 158 R ILE 159 R ASP 162 R VAL 163 R CYS 166 R THR 167 R ILE 213 R LEU 232 R ILE 233 R THR 240 R SER 243 R THR 244 R ALA 247 R PHE 343 R PHE 344 R SER 347 R LEU 370 |
| N1=C(SCCC1)Nc1c(N)cncc1 | 6.17 | R ASP 162 R VAL 163 R CYS 166 R THR 167 R ILE 233 R SER 234 R THR 240 R SER 243 R THR 244 R ALA 247 R TRP 340 R PHE 343 R PHE 344 R SER 347 R LEU 370 |
| N1=C(SCCC1)N[C@@H](c1sccc1C)C | 6.169 | R PHE 158 R ILE 159 R ASP 162 R VAL 163 R CYS 166 R THR 167 R ILE 213 R LEU 232 R ILE 233 R SER 243 R THR 244 R ALA 247 R TRP 340 R PHE 343 R PHE 344 R SER 347 R LEU 370 |
| N1=C(SCCC1)CN1[C@H](CCC[C@H]1C)C | 6.166 | R PHE 158 R ILE 159 R ASP 162 R VAL 163 R CYS 166 R THR 167 R ILE 213 R LEU 232 R ILE 233 R TYR 239 R SER 243 R THR 244 R ALA 247 R TRP 340 R PHE 343 R PHE 344 R SER 347 R LEU 370 |
| N1=C(SCCC1)Nc1cnn(c1Cl)C | 6.166 | R ILE 159 R ASP 162 R VAL 163 R THR 167 R ILE 213 R LEU 232 R ILE 233 R THR 240 R SER 243 R THR 244 R ALA 247 R PHE 343 R PHE 344 R SER 347 R LEU 370 |
| N1=C(SCCC1)Cc1[n+](CC)cccc1 | 6.162 | R PHE 158 R ILE 159 R ASP 162 R VAL 163 R CYS 166 R THR 167 R LEU 232 R ILE 233 R ALA 247 R TRP 340 R PHE 343 R PHE 344 R SER 347 R LEU 370 |
| N1=C(SCCC1)Nc1c(CO)cccc1 | 6.161 | R ILE 159 R ASP 162 R VAL 163 R ILE 213 R LEU 232 R ILE 233 R THR 240 R SER 243 R THR 244 R ALA 247 R PHE 343 R PHE 344 R SER 347 R ARG 350 |
| N1=C(SCCC1)Oc1c(CN)cccc1Cl | 6.15 | R PHE 158 R ILE 159 R ASP 162 R VAL 163 R CYS 166 R THR 167 R ILE 213 R LEU 232 R ILE 233 R SER 243 R ALA 247 R PHE 343 R PHE 344 R SER 347 R LEU 370 |
| N1=C(SCCC1)Nc1c(O)cccc1 | 6.149 | R ASP 162 R VAL 163 R ILE 213 R LEU 232 R ILE 233 R SER 243 R THR 244 R ALA 247 R PHE 343 R PHE 344 R SER 347 R ARG 350 |
| N1=C(SCCC1)N[C@@H]1[C@H](NCCC1)C | 6.147 | R PHE 158 R ILE 159 R ASP 162 R VAL 163 R CYS 166 R THR 167 R LEU 232 R ILE 233 R ALA 247 R TRP 340 R PHE 343 R PHE 344 R SER 347 R LEU 370 |
| N1=C(SCCC1)Nc1c(Cl)cncc1Cl | 6.145 | R ILE 159 R ASP 162 R VAL 163 R CYS 166 R THR 167 R ILE 213 R LEU 232 R ILE 233 R SER 243 R THR 244 R ALA 247 R TRP 340 R PHE 343 R PHE 344 R SER 347 R LEU 370 |
| N1=C(SCCC1)N[C@@H]1[C@@H](N)CCCC1 | 6.14 | R PHE 158 R ILE 159 R ASP 162 R VAL 163 R CYS 166 R THR 167 R LEU 232 R ILE 233 R TRP 340 R PHE 343 R PHE 344 R SER 347 R LEU 370 |
| N1=C(SCCC1)N(c1ccccc1)C | 6.134 | R ILE 159 R ASP 162 R VAL 163 R CYS 166 R LEU 232 R ILE 233 R TYR 239 R THR 240 R SER 243 R THR 244 R ALA 247 R PHE 343 R PHE 344 R SER 347 |
| N1=C(SCCC1)N(Cc1sccc1C)C | 6.134 | R PHE 158 R ILE 159 R ASP 162 R VAL 163 R CYS 166 R THR 167 R ILE 213 R LEU 232 R ILE 233 R ALA 247 R TRP 340 R PHE 343 R PHE 344 R SER 347 R LEU 370 |
| N1=C(SCCC1)NC(C(C)C)C(C)C | 6.122 | R PHE 158 R ILE 159 R ASP 162 R VAL 163 R CYS 166 R LEU 232 R ILE 233 R PHE 343 R PHE 344 R SER 347 R LEU 370 |
| N1=C(SCCC1)Nc1c(CC)ccnc1 | 6.108 | R ILE 159 R ASP 162 R VAL 163 R ILE 213 R LEU 232 R ILE 233 R THR 240 R SER 243 R THR 244 R ALA 247 R PHE 343 R PHE 344 R SER 347 R ARG 350 |
| N1=C(SCCC1)N[C@H]1C(OCC1)(C)C | 6.108 | R PHE 158 R ILE 159 R ASP 162 R VAL 163 R CYS 166 R LEU 232 R ILE 233 R PHE 343 R PHE 344 R SER 347 R LEU 370 |
| N1=C(SCCC1)NC1CCCCC1 | 6.097 | R PHE 158 R ILE 159 R ASP 162 R VAL 163 R CYS 166 R THR 167 R LEU 232 R ILE 233 R ALA 247 R TRP 340 R PHE 343 R PHE 344 R SER 347 R LEU 370 |
| N1=C(SCCC1)[C@H]1NCCc2c1scc2 | 6.095 | R PHE 158 R ILE 159 R ASP 162 R VAL 163 R CYS 166 R THR 167 R LEU 232 R ILE 233 R SER 243 R THR 244 R ALA 247 R PHE 343 R PHE 344 R SER 347 |
| N1=C(SCCC1)N[C@H]1[C@@H](CCNC1)C | 6.089 | R PHE 158 R ILE 159 R ASP 162 R VAL 163 R CYS 166 R CYS 231 R LEU 232 R ILE 233 R PHE 343 R PHE 344 R SER 347 R ARG 367 R LEU 370 |
| N1=C(SCCC1)Cc1c(O)cccc1Cl | 6.084 | R VAL 138 R PHE 158 R ILE 159 R ASP 162 R VAL 163 R CYS 231 R LEU 232 R ILE 233 R PHE 343 R ARG 350 R LEU 363 R GLU 366 R ARG 367 R LEU 370 |
| N1=C(SCCC1)NCc1scc(c1)C | 6.077 | R ASP 162 R VAL 163 R CYS 166 R THR 167 R ILE 213 R LEU 232 R ILE 233 R GLN 235 R SER 243 R THR 244 R ALA 247 R PHE 343 R PHE 344 R SER 347 R ARG 350 R LEU 370 |
| N1=C(SCCC1)Nc1cccnc1Cl | 6.06 | R PHE 158 R ILE 159 R ASP 162 R VAL 163 R LEU 232 R ILE 233 R PHE 343 R SER 347 R ARG 350 R GLU 366 R ARG 367 R LEU 370 |
| N1=C(SCCC1)Sc1c(cccc1C)C | 6.059 | R ASP 162 R VAL 163 R CYS 166 R THR 167 R ILE 213 R ILE 233 R SER 234 R TYR 239 R THR 240 R SER 243 R THR 244 R TRP 340 R PHE 343 R PHE 344 R SER 347 |
| N1=C(SCCC1)Nc1c(N)ccnc1 | 6.057 | R ASP 162 R VAL 163 R CYS 166 R THR 167 R ILE 233 R THR 240 R SER 243 R THR 244 R ALA 247 R TRP 340 R PHE 343 R PHE 344 R SER 347 R LEU 370 |
| N1=C(SCCC1)N[C@@H]1[C@H](OCC1)CC | 6.039 | R PHE 158 R ILE 159 R ASP 162 R VAL 163 R CYS 166 R THR 167 R CYS 231 R LEU 232 R ILE 233 R PHE 343 R PHE 344 R SER 347 R LEU 370 R TYR 374 |
| N1=C(SCCC1)Nn1c(C)ccc1C | 6.036 | R ASP 162 R VAL 163 R CYS 166 R THR 167 R ILE 213 R ILE 233 R SER 234 R TYR 239 R THR 240 R SER 243 R THR 244 R ALA 247 R TRP 340 R PHE 343 R PHE 344 R SER 347 |
| N1=C(SCCC1)[C@H]1NCCc2sccc12 | 6.034 | R PHE 158 R ILE 159 R ASP 162 R VAL 163 R CYS 166 R THR 167 R LEU 232 R ILE 233 R SER 243 R TRP 340 R PHE 343 R PHE 344 R SER 347 R LEU 370 |
| N1=C(SCCC1)Nc1c(N(C)C)ccc(Cl)c1 | 6.029 | R ILE 159 R ASP 162 R VAL 163 R CYS 166 R THR 167 R ILE 213 R ILE 233 R SER 234 R TYR 239 R THR 240 R SER 243 R THR 244 R ALA 247 R TRP 340 R PHE 343 R PHE 344 R SER 347 R THR 348 |
| N1=C(SCCC1)N[C@@H](C(C)C)C(=O)NC | 6.016 | R PHE 158 R ILE 159 R ASP 162 R VAL 163 R CYS 166 R THR 167 R LEU 232 R ILE 233 R TYR 239 R THR 240 R SER 243 R PHE 343 R PHE 344 R SER 347 R LEU 370 |
| N1=C(SCCC1)Nc1c([nH]nc1C)C | 6.009 | R ASP 162 R VAL 163 R CYS 166 R ILE 213 R ILE 233 R SER 234 R GLN 235 R THR 240 R SER 243 R THR 244 R ALA 247 R PHE 343 R PHE 344 R SER 347 R LEU 370 |
| N1=C(SCCC1)N[C@@H]1CCC=CC1 | 6.003 | R PHE 158 R ILE 159 R ASP 162 R VAL 163 R CYS 166 R THR 167 R LEU 232 R ILE 233 R ALA 247 R TRP 340 R PHE 343 R PHE 344 R SER 347 R LEU 370 |
| N1=C(SCCC1)NCc1c(occ1)C | 5.997 | R ASP 162 R VAL 163 R CYS 166 R THR 167 R ILE 233 R SER 234 R TYR 239 R THR 240 R SER 243 R THR 244 R ALA 247 R TRP 340 R PHE 343 R PHE 344 R SER 347 R LEU 370 |
| N1=C(SCCC1)N[C@](C(C)C)(C)C#N | 5.989 | R PHE 158 R ILE 159 R ASP 162 R VAL 163 R LEU 232 R ILE 233 R PHE 343 R PHE 344 R SER 347 R LEU 370 |
| N1=C(SCCC1)NCc1nc[nH]c1C | 5.979 | R VAL 163 R ILE 213 R LEU 232 R ILE 233 R TYR 239 R THR 240 R SER 243 R THR 244 R PHE 343 R PHE 344 R SER 347 R ARG 350 R LEU 370 |
| N1=C(SCCC1)N[C@H](CC)c1sccc1 | 5.977 | R PHE 158 R ILE 159 R ASP 162 R VAL 163 R CYS 166 R THR 167 R LEU 232 R ILE 233 R PHE 343 R PHE 344 R SER 347 R ARG 350 R LEU 370 |
| N1=C(SCCC1)N[C@@H](C(O)(C)C)C(C)C | 5.97 | R PHE 158 R ILE 159 R ASP 162 R VAL 163 R CYS 166 R LEU 232 R ILE 233 R SER 243 R PHE 343 R PHE 344 R SER 347 R ARG 350 R LEU 370 |
| N1=C(SCCC1)N[C@@H](C(C)C)C(=O)OC | 5.969 | R PHE 158 R ILE 159 R ASP 162 R VAL 163 R CYS 166 R THR 167 R LEU 232 R ILE 233 R TYR 239 R THR 240 R SER 243 R PHE 343 R PHE 344 R SER 347 R LEU 370 |
| N1=C(SCCC1)N[C@H](CC)C(=O)N | 5.964 | R ASP 162 R VAL 163 R CYS 166 R THR 167 R ILE 213 R ILE 233 R TYR 239 R THR 240 R SER 243 R THR 244 R ALA 247 R TRP 340 R PHE 343 R PHE 344 R SER 347 |
| N1=C(SCCC1)N[C@H](CC)C(F)(F)F | 5.96 | R ASP 162 R VAL 163 R CYS 166 R THR 167 R ILE 233 R SER 234 R TYR 239 R THR 240 R SER 243 R THR 244 R ALA 247 R TRP 340 R PHE 343 R PHE 344 R SER 347 |
| N1=C(SCCC1)NC[C@@H]1N(CCC1)CC | 5.953 | R PHE 158 R ILE 159 R ASP 162 R VAL 163 R CYS 166 R THR 167 R ILE 213 R CYS 231 R LEU 232 R ILE 233 R TYR 239 R SER 243 R ALA 247 R PHE 343 R PHE 344 R SER 347 R LEU 370 |
| N1=C(SCCC1)NC[C@H]1N(CCC1)C | 5.945 | R PHE 158 R ILE 159 R ASP 162 R VAL 163 R THR 167 R ILE 213 R LEU 232 R ILE 233 R SER 243 R THR 244 R ALA 247 R PHE 343 R PHE 344 R SER 347 |
| N1=C(SCCC1)Cc1c(Cl)cncc1Cl | 5.923 | R PHE 158 R ILE 159 R ASP 162 R VAL 163 R CYS 166 R LEU 232 R ILE 233 R SER 243 R PHE 343 R PHE 344 R SER 347 R LEU 370 |
| N1=C(SCCC1)N1C[C@@H]2N(CC1)CCC2 | 5.915 | R ILE 159 R ASP 162 R VAL 163 R ILE 213 R LEU 232 R ILE 233 R TYR 239 R THR 240 R SER 243 R THR 244 R ALA 247 R PHE 343 R PHE 344 R SER 347 R LEU 370 |
| N1=C(SCCC1)Nc1c(OC)ccnc1 | 5.912 | R ASP 162 R VAL 163 R ILE 213 R LEU 232 R ILE 233 R THR 240 R SER 243 R THR 244 R ALA 247 R PHE 343 R PHE 344 R SER 347 R THR 348 R LEU 370 |
| N1=C(SCCC1)Nc1c(O)ccnc1 | 5.909 | R ASP 162 R VAL 163 R CYS 166 R THR 167 R ILE 233 R THR 240 R SER 243 R THR 244 R ALA 247 R TRP 340 R PHE 343 R PHE 344 R SER 347 R LEU 370 |
| N1=C(SCCC1)NC1(C2CC2)CC1 | 5.904 | R PHE 158 R ILE 159 R ASP 162 R VAL 163 R CYS 166 R LEU 232 R ILE 233 R PHE 343 R PHE 344 R SER 347 R LEU 370 |
| N1=C(SCCC1)N[C@H](c1sccc1)C | 5.898 | R PHE 158 R ILE 159 R ASP 162 R VAL 163 R THR 167 R ILE 213 R ILE 233 R SER 243 R THR 244 R ALA 247 R PHE 343 R PHE 344 R SER 347 |
| N1=C(SCCC1)Nc1c(nn(c1)C)CC | 5.885 | R PHE 158 R ILE 159 R ASP 162 R VAL 163 R LEU 232 R ILE 233 R GLN 235 R PHE 343 R SER 347 R ARG 350 R GLU 366 R ARG 367 R LEU 370 |
| N1=C(SCCC1)N[C@@H](C(C)C)C(=O)N | 5.869 | R PHE 158 R ILE 159 R ASP 162 R VAL 163 R CYS 166 R THR 167 R LEU 232 R ILE 233 R TRP 340 R PHE 343 R PHE 344 R SER 347 R LEU 370 |
| N1=C(SCCC1)Nc1c(N(C)C)cccc1 | 5.868 | R PHE 158 R ILE 159 R ASP 162 R VAL 163 R CYS 166 R LEU 232 R ILE 233 R TRP 340 R PHE 343 R PHE 344 R SER 347 R LEU 370 |
| N1=C(SCCC1)N[C@H]1[C@@H](COCC1)C | 5.867 | R PHE 158 R ILE 159 R ASP 162 R VAL 163 R LEU 232 R ILE 233 R PHE 343 R PHE 344 R SER 347 R ARG 367 R LEU 370 |
| N1=C(SCCC1)NC1CCOCC1 | 5.864 | R ASP 162 R VAL 163 R CYS 166 R THR 167 R ILE 233 R SER 234 R TYR 239 R THR 240 R SER 243 R THR 244 R ALA 247 R TRP 340 R PHE 343 R PHE 344 R SER 347 |
| N1=C(SCCC1)N[C@@H](CC)c1sccn1 | 5.855 | R VAL 163 R THR 167 R ILE 213 R LEU 232 R ILE 233 R SER 234 R GLN 235 R TYR 239 R THR 240 R SER 243 R THR 244 R ALA 247 R PHE 343 R PHE 344 R SER 347 R ARG 350 R LEU 370 |
| N1=C(SCCC1)N[C@H](C(CC)CC)C | 5.853 | R PHE 158 R ILE 159 R ASP 162 R VAL 163 R THR 167 R ILE 213 R LEU 232 R ILE 233 R SER 243 R THR 244 R ALA 247 R PHE 343 R PHE 344 R SER 347 R LEU 370 |
| N1=C(SCCC1)N[C@@H]1[C@H](OCC1)C=C | 5.845 | R ASP 162 R VAL 163 R CYS 166 R THR 167 R ILE 213 R ILE 233 R SER 234 R TYR 239 R THR 240 R SER 243 R THR 244 R ALA 247 R TRP 340 R PHE 343 R PHE 344 R SER 347 R LEU 370 |
| N1=C(SCCC1)N[C@H](C(O)(C)C)CC | 5.829 | R PHE 158 R ILE 159 R ASP 162 R VAL 163 R CYS 166 R THR 167 R LEU 232 R ILE 233 R PHE 343 R PHE 344 R SER 347 R LEU 370 |
| N1=C(SCCC1)Nc1c(csc1)C | 5.809 | R ILE 159 R ASP 162 R VAL 163 R CYS 166 R THR 167 R ILE 213 R LEU 232 R ILE 233 R SER 243 R THR 244 R ALA 247 R PHE 343 R PHE 344 R SER 347 |
| N1=C(SCCC1)N1[C@@H]([C@H](SCC1)C)C | 5.802 | R ILE 159 R ASP 162 R VAL 163 R CYS 166 R THR 167 R ILE 213 R LEU 232 R ILE 233 R SER 243 R THR 244 R ALA 247 R TRP 340 R PHE 343 R PHE 344 R SER 347 R LEU 370 |
| N1=C(SCCC1)CN1[C@@H](CN(CC1)C)C | 5.801 | R ASP 162 R VAL 163 R CYS 166 R THR 167 R ILE 213 R ILE 233 R SER 234 R TYR 239 R THR 240 R SER 243 R THR 244 R ALA 247 R TRP 340 R PHE 343 R PHE 344 R SER 347 R LEU 370 R TYR 374 |
| N1=C(SCCC1)NCC1(OCCO1)C | 5.799 | R VAL 163 R THR 167 R ILE 213 R LEU 232 R ILE 233 R GLN 235 R TYR 239 R THR 240 R SER 243 R THR 244 R ALA 247 R PHE 343 R PHE 344 R SER 347 R ARG 350 R LEU 370 |
| N1=C(SCCC1)NCc1cc[nH]c1 | 5.797 | R ASP 162 R VAL 163 R CYS 166 R THR 167 R ILE 233 R SER 234 R TYR 239 R THR 240 R SER 243 R THR 244 R ALA 247 R TRP 340 R PHE 343 R PHE 344 R SER 347 R LEU 370 |
| N1=C(SCCC1)N/C=C(/CC)C | 5.793 | R ILE 159 R ASP 162 R VAL 163 R CYS 166 R THR 167 R ILE 213 R LEU 232 R ILE 233 R SER 243 R THR 244 R ALA 247 R PHE 343 R PHE 344 R SER 347 R LEU 370 |
| N1=C(SCCC1)N1CCSC[C@@H](C1)C | 5.772 | R PHE 158 R ILE 159 R ASP 162 R VAL 163 R CYS 231 R LEU 232 R ILE 233 R PHE 343 R ARG 350 R GLU 366 R ARG 367 R LEU 370 |
| N1=C(SCCC1)N[C@@H](C(C)C)C#C | 5.747 | R PHE 158 R ILE 159 R ASP 162 R VAL 163 R CYS 166 R LEU 232 R ILE 233 R PHE 343 R PHE 344 R SER 347 R LEU 370 |
| N1=C(SCCC1)[C@@H]1[C@@H](N(CC1)C)C | 5.747 | R ASP 162 R VAL 163 R CYS 166 R THR 167 R ILE 213 R ILE 233 R SER 234 R TYR 239 R THR 240 R SER 243 R THR 244 R ALA 247 R TRP 340 R PHE 343 R PHE 344 R SER 347 |
| N1=C(SCCC1)NCc1sccc1Br | 5.718 | R PHE 158 R ILE 159 R ASP 162 R VAL 163 R CYS 166 R THR 167 R ILE 213 R LEU 232 R ILE 233 R SER 243 R THR 244 R ALA 247 R PHE 343 R PHE 344 R SER 347 R LEU 370 |
| N1=C(SCCC1)N[C@@H](C)c1sccn1 | 5.69 | R ASP 162 R VAL 163 R CYS 166 R THR 167 R ILE 213 R ILE 233 R TYR 239 R THR 240 R SER 243 R THR 244 R TRP 340 R PHE 343 R PHE 344 R SER 347 |
| N1=C(SCCC1)[C@@H](N)c1sccc1 | 5.678 | R ASP 162 R VAL 163 R CYS 166 R THR 167 R ILE 213 R ILE 233 R SER 234 R TYR 239 R THR 240 R SER 243 R THR 244 R ALA 247 R TRP 340 R PHE 343 R PHE 344 R SER 347 |
| N1=C(SCCC1)NCc1ccsc1 | 5.669 | R ILE 159 R ASP 162 R VAL 163 R THR 167 R ILE 213 R LEU 232 R ILE 233 R SER 243 R THR 244 R ALA 247 R PHE 343 R PHE 344 R SER 347 |
| N1=C(SCCC1)NCc1c(Cl)csc1 | 5.667 | R PHE 158 R ILE 159 R ASP 162 R VAL 163 R CYS 166 R THR 167 R ILE 233 R ALA 247 R PHE 343 R PHE 344 R SER 347 R ARG 367 R LEU 370 |
| N1=C(SCCC1)Nc1csnc1C | 5.642 | R ASP 162 R VAL 163 R CYS 166 R THR 167 R ILE 213 R ILE 233 R THR 240 R SER 243 R THR 244 R ALA 247 R TRP 340 R PHE 343 R PHE 344 R SER 347 |
| N1=C(SCCC1)N[C@H](C)C(=O)C | 5.604 | R ASP 162 R VAL 163 R CYS 166 R THR 167 R ILE 233 R SER 234 R TYR 239 R THR 240 R SER 243 R THR 244 R ALA 247 R TRP 340 R PHE 343 R PHE 344 R SER 347 R LEU 370 |
| N1=C(SCCC1)N[C@H]1CC[S@@](=O)C1 | 5.601 | R PHE 158 R ILE 159 R ASP 162 R VAL 163 R CYS 166 R THR 167 R CYS 231 R LEU 232 R ILE 233 R TRP 340 R PHE 343 R PHE 344 R SER 347 |
| N1=C(SCCC1)CN([C@@H]1CCN(C1)C)C | 5.599 | R PHE 158 R ILE 159 R ASP 162 R VAL 163 R LEU 232 R ILE 233 R TYR 239 R THR 240 R SER 243 R THR 244 R PHE 343 R PHE 344 R SER 347 R LEU 370 |
| N1=C(SCCC1)NC1CCSCC1 | 5.598 | R PHE 158 R ILE 159 R ASP 162 R VAL 163 R CYS 166 R THR 167 R LEU 232 R ILE 233 R PHE 343 R PHE 344 R SER 347 R LEU 370 |
| N1=C(SCCC1)N[C@H]1[C@H](SCC1)C | 5.595 | R ASP 162 R VAL 163 R CYS 166 R THR 167 R ILE 233 R TYR 239 R THR 240 R SER 243 R THR 244 R ALA 247 R TRP 340 R PHE 343 R PHE 344 R SER 347 R LEU 370 |
| N1=C(SCCC1)N[C@@H](C)C(=O)N | 5.577 | R ASP 162 R VAL 163 R CYS 166 R THR 167 R ILE 213 R ILE 233 R TYR 239 R THR 240 R SER 243 R THR 244 R ALA 247 R TRP 340 R PHE 343 R PHE 344 R SER 347 R THR 348 |
| N1=C(SCCC1)N1C[C@H](F)[C@@H](F)C1 | 5.57 | R ASP 162 R VAL 163 R CYS 166 R THR 167 R ILE 213 R ILE 233 R SER 234 R TYR 239 R THR 240 R SER 243 R THR 244 R ALA 247 R TRP 340 R PHE 343 R PHE 344 R SER 347 |
| N1=C(SCCC1)N[C@@H](CCCN)C | 5.563 | R ASP 162 R VAL 163 R CYS 166 R THR 167 R ILE 233 R SER 234 R TYR 239 R THR 240 R ILE 241 R SER 243 R THR 244 R ALA 247 R TRP 340 R PHE 343 R PHE 344 R SER 347 R THR 348 |
| N1=C(SCCC1)N[C@H](C(C)C)C | 5.551 | R PHE 158 R ILE 159 R ASP 162 R VAL 163 R CYS 166 R THR 167 R LEU 232 R ILE 233 R PHE 343 R PHE 344 R SER 347 R LEU 370 |
| N1=C(SCCC1)N[C@@H](C(C)C)CC | 5.544 | R PHE 158 R ILE 159 R ASP 162 R VAL 163 R CYS 166 R THR 167 R LEU 232 R ILE 233 R PHE 343 R PHE 344 R SER 347 R LEU 370 |
| N1=C(SCCC1)N[C@@H](CN(C)C)C | 5.522 | R ASP 162 R VAL 163 R CYS 166 R THR 167 R ILE 233 R TYR 239 R THR 240 R SER 243 R THR 244 R ALA 247 R TRP 340 R PHE 343 R PHE 344 R SER 347 R THR 348 R LEU 370 |
| N1=C(SCCC1)NCC1(CC1)C | 5.522 | R PHE 158 R ILE 159 R ASP 162 R VAL 163 R CYS 166 R THR 167 R LEU 232 R ILE 233 R ALA 247 R PHE 343 R PHE 344 R SER 347 R LEU 370 R TYR 374 |
| N1=C(SCCC1)N[C@H](CN)C(C)C | 5.52 | R ASP 162 R VAL 163 R CYS 166 R THR 167 R ILE 213 R ILE 233 R SER 234 R TYR 239 R THR 240 R SER 243 R THR 244 R ALA 247 R TRP 340 R PHE 343 R PHE 344 R SER 347 R THR 348 |
| N1=C(SCCC1)Nc1c([nH+]ccc1)N(C)C | 5.509 | R PHE 158 R ILE 159 R ASP 162 R VAL 163 R CYS 166 R LEU 232 R ILE 233 R TRP 340 R PHE 343 R PHE 344 R SER 347 R LEU 370 |
| N1=C(SCCC1)N[C@H]1CCN(C1)C | 5.453 | R PHE 158 R ILE 159 R ASP 162 R VAL 163 R CYS 166 R THR 167 R LEU 232 R ILE 233 R ALA 247 R PHE 343 R PHE 344 R SER 347 R LEU 370 |
| N1=C(SCCC1)[C@@H](CN)c1sccc1 | 5.417 | R ASP 162 R VAL 163 R CYS 166 R THR 167 R ILE 213 R ILE 233 R SER 234 R TYR 239 R THR 240 R SER 243 R THR 244 R ALA 247 R PHE 343 R PHE 344 R SER 347 |
| N1=C(SCCC1)N[C@@H](CCC=C)C | 5.41 | R ILE 159 R ASP 162 R VAL 163 R CYS 166 R THR 167 R ILE 213 R ILE 233 R SER 234 R TYR 239 R THR 240 R SER 243 R THR 244 R ALA 247 R PHE 343 R PHE 344 R SER 347 |
| N1=C(SCCC1)N[C@@H]([C@@H](CO)C)C | 5.406 | R ASP 162 R VAL 163 R CYS 166 R THR 167 R ILE 213 R ILE 233 R TYR 239 R THR 240 R SER 243 R THR 244 R ALA 247 R TRP 340 R PHE 343 R PHE 344 R SER 347 R LEU 370 |
| N1=C(SCCC1)N1C[C@@H]2N[C@@H](C2)C1 | 5.351 | R ASP 162 R VAL 163 R CYS 166 R THR 167 R ILE 213 R ILE 233 R TYR 239 R THR 240 R SER 243 R THR 244 R ALA 247 R TRP 340 R PHE 343 R PHE 344 R SER 347 R LEU 370 |
| N1=C(SCCC1)N[C@H](CF)C | 5.17 | R VAL 163 R CYS 166 R THR 167 R ILE 213 R ILE 233 R SER 234 R TYR 239 R THR 240 R SER 243 R THR 244 R ALA 247 R PHE 343 R PHE 344 R SER 347 |

Table S2: SMILES, Bind.energy and Contacting receptor residues for the 500 molecules resulted from the scaffold-hopping approach for KOR.

| SMILES | Bind.energy[kcal/mol] | Contacting receptor residues |
| --- | --- | --- |
| N1=C(SCCC1)Nc1c2CCCc2cc2CCCc12 | 9.008 | A VAL 108 A THR 111 A PHE 114 A GLN 115 A TRP 124 A VAL 134 A ASP 138 A MET 142 A TRP 287 A ILE 290 A ILE 294 A TYR 312 A ILE 316 A GLY 319 A TYR 320 |
| N1=C(SCCC1)Nc1c2OC(Cc2c(cc1C)C)(C)C | 8.926 | A VAL 108 A THR 111 A PHE 114 A GLN 115 A VAL 118 A TYR 119 A TRP 124 A VAL 134 A LEU 135 A ASP 138 A GLU 209 A CYS 210 A LEU 309 A TYR 312 A TYR 313 A TYR 320 |
| N1=C(SCCC1)Nc1c(N)cc(cc1)C(F)(F)F | 8.287 | A VAL 108 A THR 111 A GLN 115 A TRP 124 A VAL 134 A LEU 135 A ASP 138 A TRP 287 A ILE 290 A CYS 315 A ILE 316 A GLY 319 A TYR 320 |
| N1=C(SCCC1)Nc1c2c(ccc1)cccc2O | 8.259 | A VAL 108 A THR 111 A GLN 115 A VAL 118 A TRP 124 A VAL 134 A LEU 135 A ILE 137 A ASP 138 A CYS 210 A TYR 312 A ILE 316 A TYR 320 |
| N1=C(SCCC1)N[C@@H]1CCCOc2c1ccc(F)c2 | 8.226 | A VAL 108 A ASP 138 A TYR 139 A MET 142 A LYS 227 A VAL 230 A TRP 287 A ILE 290 A HIS 291 A ILE 294 A TYR 312 A ILE 316 A GLY 319 A TYR 320 |
| N1=C(SCCC1)Cc1c2c(ccc1C)cccc2 | 8.052 | A VAL 108 A THR 111 A GLN 115 A VAL 134 A LEU 135 A ASP 138 A TRP 287 A ILE 290 A TYR 312 A ILE 316 A GLY 319 A TYR 320 |
| N1=C(SCCC1)Nc1c(cc(cc1)C(F)(F)F)C | 8.04 | A VAL 108 A THR 111 A GLN 115 A TRP 124 A VAL 134 A LEU 135 A ILE 137 A ASP 138 A ASN 141 A CYS 210 A TRP 287 A ILE 316 A GLY 319 A TYR 320 |
| N1=C(SCCC1)Nc1c2c(sc1N)cccc2 | 7.918 | A VAL 108 A ASP 138 A TYR 139 A MET 142 A LYS 227 A VAL 230 A TRP 287 A ILE 290 A HIS 291 A ILE 294 A TYR 312 A ILE 316 A GLY 319 A TYR 320 |
| N1=C(SCCC1)Nc1c(c2c([nH]cc2)cc1)C | 7.905 | A THR 111 A PHE 114 A GLN 115 A TRP 124 A VAL 134 A LEU 135 A ASP 138 A TRP 287 A ILE 290 A CYS 315 A ILE 316 A GLY 319 A TYR 320 |
| N1=C(SCCC1)Nc1c2c(ccc1)cccc2 | 7.893 | A VAL 108 A THR 111 A GLN 115 A VAL 118 A TRP 124 A VAL 134 A LEU 135 A ILE 137 A ASP 138 A CYS 210 A SER 211 A TYR 312 A ILE 316 A TYR 320 |
| N1=C(SCCC1)Nc1cc(C(C)C)ccc1C | 7.876 | A VAL 108 A THR 111 A GLN 115 A TRP 124 A VAL 134 A LEU 135 A ASP 138 A CYS 210 A TRP 287 A ILE 290 A TYR 312 A ILE 316 A GLY 319 A TYR 320 |
| N1=C(SCCC1)Nc1c(n2nccc2)cccc1 | 7.865 | A VAL 108 A THR 111 A GLN 115 A VAL 134 A LEU 135 A ASP 138 A MET 142 A TRP 287 A ILE 290 A ILE 294 A ILE 316 A GLY 319 A TYR 320 |
| N1=C(SCCC1)Nc1c(N)ccc(c1)C(=O)N | 7.861 | A VAL 108 A ASP 138 A TYR 139 A MET 142 A VAL 230 A TRP 287 A ILE 290 A HIS 291 A ILE 294 A TYR 312 A ILE 316 A GLY 319 A TYR 320 |
| N1=C(SCCC1)Nc1c2[nH]ccc2ccc1 | 7.826 | A VAL 108 A THR 111 A GLN 115 A TRP 124 A VAL 134 A LEU 135 A ASP 138 A TRP 287 A ILE 290 A TYR 312 A ILE 316 A GLY 319 A TYR 320 |
| N1=C(SCCC1)NCc1c(F)cc(F)cc1 | 7.824 | A VAL 108 A THR 111 A PHE 114 A GLN 115 A TRP 124 A VAL 134 A LEU 135 A ASP 138 A TRP 287 A ILE 290 A ILE 316 A GLY 319 A TYR 320 |
| N1=C(SCCC1)Nc1ccc(cc1F)C(F)(F)F | 7.793 | A VAL 108 A THR 111 A TRP 124 A VAL 134 A LEU 135 A ASP 138 A TRP 287 A ILE 290 A TYR 312 A ILE 316 A GLY 319 A TYR 320 |
| N1=C(SCCC1)Nc1c2c(ccc1)c(=O)[nH]cc2 | 7.792 | A GLN 115 A TRP 124 A VAL 134 A LEU 135 A ASP 138 A TRP 287 A ILE 290 A ILE 294 A TYR 312 A ILE 316 A GLY 319 A TYR 320 |
| N1=C(SCCC1)Nc1c2c(CC[C@H](O)C2)ccc1 | 7.784 | A VAL 108 A THR 111 A PHE 114 A GLN 115 A TRP 124 A VAL 134 A ASP 138 A MET 142 A TRP 287 A ILE 290 A ILE 294 A TYR 312 A ILE 316 A GLY 319 A TYR 320 |
| N1=C(SCCC1)Nc1c(n2ccnc2)cccc1 | 7.781 | A VAL 108 A THR 111 A PHE 114 A GLN 115 A VAL 134 A LEU 135 A ASP 138 A MET 142 A TRP 287 A ILE 290 A ILE 294 A TYR 312 A ILE 316 A GLY 319 A TYR 320 |
| N1=C(SCCC1)N1c2c(CCC1)c(ccc2)C(=O)N | 7.777 | A LEU 135 A ASP 138 A TYR 139 A MET 142 A SER 211 A LEU 212 A TYR 219 A ASP 223 A LYS 227 A VAL 230 A TRP 287 A ILE 290 A HIS 291 A ILE 294 A TYR 312 A ILE 316 |
| N1=C(SCCC1)Nc1c2CCCc2ccc1OC | 7.771 | A VAL 108 A THR 111 A PHE 114 A GLN 115 A TRP 124 A VAL 134 A ASP 138 A TRP 287 A ILE 290 A ILE 294 A TYR 312 A ILE 316 A GLY 319 A TYR 320 |
| N1=C(SCCC1)Nc1c(NC(C)C)cccc1F | 7.755 | A VAL 108 A THR 111 A GLN 115 A ASP 138 A TYR 139 A TRP 287 A ILE 290 A ILE 294 A TYR 312 A ILE 316 A GLY 319 A TYR 320 |
| N1=C(SCCC1)Nc1c(CC(F)(F)F)cccc1 | 7.748 | A VAL 108 A THR 111 A GLN 115 A VAL 134 A ASP 138 A MET 142 A TRP 287 A ILE 290 A TYR 312 A CYS 315 A ILE 316 A GLY 319 A TYR 320 |
| N1=C(SCCC1)Nc1c(c2sccc2)cccc1 | 7.737 | A VAL 108 A THR 111 A GLN 115 A TRP 124 A VAL 134 A LEU 135 A ASP 138 A MET 142 A TRP 287 A ILE 290 A ILE 294 A TYR 312 A ILE 316 A GLY 319 A TYR 320 |
| N1=C(SCCC1)Nc1cc(C(F)(F)F)ccc1C | 7.709 | A VAL 108 A THR 111 A GLN 115 A TRP 124 A VAL 134 A LEU 135 A ILE 137 A ASP 138 A CYS 210 A SER 211 A TRP 287 A TYR 312 A ILE 316 A GLY 319 A TYR 320 |
| N1=C(SCCC1)Nc1c(c2c([nH]cc2)nc1)C | 7.666 | A VAL 108 A THR 111 A GLN 115 A TRP 124 A VAL 134 A LEU 135 A ILE 137 A ASP 138 A TRP 287 A ILE 290 A TYR 312 A CYS 315 A ILE 316 A GLY 319 A TYR 320 |
| N1=C(SCCC1)Nc1c(O)cc(c(c1)C)C | 7.652 | A VAL 108 A ASP 138 A TYR 139 A MET 142 A LYS 227 A VAL 230 A TRP 287 A ILE 290 A HIS 291 A ILE 294 A ILE 316 A GLY 319 A TYR 320 |
| N1=C(SCCC1)N[C@@H](c1c(F)cc(F)cc1)C | 7.652 | A VAL 108 A THR 111 A PHE 114 A GLN 115 A TRP 124 A VAL 134 A LEU 135 A ASP 138 A TRP 287 A ILE 290 A ILE 316 A GLY 319 A TYR 320 |
| N1=C(SCCC1)Nc1cc(ccc1Cl)C(F)(F)F | 7.64 | A VAL 108 A THR 111 A GLN 115 A VAL 134 A LEU 135 A ILE 137 A ASP 138 A CYS 210 A SER 211 A TRP 287 A TYR 312 A ILE 316 A TYR 320 |
| N1=C(SCCC1)Nc1c2nc(oc2ccc1)C | 7.631 | A VAL 108 A THR 111 A GLN 115 A VAL 118 A TRP 124 A VAL 134 A LEU 135 A ILE 137 A ASP 138 A CYS 210 A TYR 312 A ILE 316 A TYR 320 |
| N1=C(SCCC1)Nc1c2c(ccc1O)cccc2 | 7.627 | A THR 111 A GLN 115 A TRP 124 A VAL 134 A LEU 135 A ASP 138 A MET 142 A TRP 287 A ILE 290 A ILE 294 A TYR 312 A ILE 316 A GLY 319 A TYR 320 |
| N1=C(SCCC1)Nc1cc(C(C)(C)C)ccc1C | 7.626 | A VAL 108 A THR 111 A GLN 115 A TRP 124 A VAL 134 A LEU 135 A ILE 137 A ASP 138 A ASN 141 A CYS 210 A TRP 287 A ILE 290 A TYR 312 A ILE 316 A GLY 319 A TYR 320 |
| N1=C(SCCC1)Nc1c(c(ccc1)C(F)(F)F)C | 7.625 | A VAL 108 A THR 111 A GLN 115 A TRP 124 A VAL 134 A LEU 135 A ASP 138 A TRP 287 A ILE 290 A TYR 312 A ILE 316 A GLY 319 A TYR 320 |
| N1=C(SCCC1)Nc1c2CCCCc2ccc1 | 7.624 | A LEU 135 A ASP 138 A TYR 139 A MET 142 A SER 211 A LEU 212 A LYS 227 A VAL 230 A TRP 287 A ILE 290 A HIS 291 A ILE 294 A TYR 312 A ILE 316 |
| N1=C(SCCC1)NCc1ccc(F)cc1 | 7.599 | A VAL 108 A THR 111 A PHE 114 A GLN 115 A TRP 124 A VAL 134 A LEU 135 A ASP 138 A TRP 287 A ILE 290 A CYS 315 A ILE 316 A GLY 319 A TYR 320 |
| N1=C(SCCC1)Nc1c(cc2c([nH]cc2)c1)C | 7.597 | A VAL 108 A THR 111 A GLN 115 A TRP 124 A VAL 134 A LEU 135 A ASP 138 A ASN 141 A CYS 210 A TRP 287 A ILE 316 A GLY 319 A TYR 320 |
| N1=C(SCCC1)NCc1c(F)cccc1 | 7.595 | A VAL 108 A THR 111 A GLN 115 A TRP 124 A VAL 134 A LEU 135 A ILE 137 A ASP 138 A TRP 287 A ILE 290 A CYS 315 A ILE 316 A GLY 319 A TYR 320 |
| N1=C(SCCC1)Nc1c(cc(F)cc1)C(F)(F)F | 7.594 | A VAL 108 A GLN 115 A ASP 138 A TYR 139 A MET 142 A VAL 230 A TRP 287 A ILE 290 A ILE 294 A TYR 312 A ILE 316 A GLY 319 A TYR 320 |
| N1=C(SCCC1)C[n+]1c2c(ccc1)cccc2 | 7.583 | A VAL 108 A THR 111 A GLN 115 A VAL 134 A LEU 135 A ILE 137 A ASP 138 A TRP 287 A ILE 290 A TYR 312 A ILE 316 A GLY 319 A TYR 320 |
| N1=C(SCCC1)Nc1c(F)cc(F)cc1F | 7.581 | A VAL 108 A THR 111 A GLN 115 A VAL 134 A ASP 138 A TRP 287 A ILE 290 A TYR 312 A ILE 316 A GLY 319 A TYR 320 |
| N1=C(SCCC1)N[C@@H](c1cc(ccc1)C)C | 7.578 | A VAL 108 A THR 111 A PHE 114 A GLN 115 A TRP 124 A VAL 134 A LEU 135 A ASP 138 A MET 142 A TRP 287 A ILE 290 A ILE 316 A GLY 319 A TYR 320 |
| N1=C(SCCC1)Nc1c(Cl)c2c([nH]nc2)cc1 | 7.576 | A VAL 108 A THR 111 A PHE 114 A GLN 115 A TRP 124 A VAL 134 A ASP 138 A TRP 287 A ILE 290 A ILE 316 A GLY 319 A TYR 320 |
| N1=C(SCCC1)Nc1c(F)c(F)cc(F)c1F | 7.574 | A VAL 108 A THR 111 A GLN 115 A VAL 118 A TRP 124 A VAL 134 A LEU 135 A ILE 137 A ASP 138 A CYS 210 A TYR 312 A ILE 316 A TYR 320 |
| N1=C(SCCC1)Nc1cc(C(C)C)ccc1O | 7.573 | A VAL 108 A THR 111 A PHE 114 A GLN 115 A TRP 124 A VAL 134 A ASP 138 A TRP 287 A ILE 290 A TYR 312 A CYS 315 A ILE 316 A GLY 319 A TYR 320 |
| N1=C(SCCC1)Nc1c(N)cc(Cl)c(F)c1 | 7.561 | A VAL 108 A THR 111 A GLN 115 A TRP 124 A VAL 134 A LEU 135 A ASP 138 A TRP 287 A ILE 290 A ILE 316 A GLY 319 A TYR 320 |
| N1=C(SCCC1)Nc1c(cc(cc1C)C#N)C | 7.557 | A VAL 108 A THR 111 A PHE 114 A GLN 115 A VAL 118 A TRP 124 A VAL 134 A LEU 135 A ASP 138 A ILE 208 A GLU 209 A CYS 210 A TYR 312 A TYR 313 A TYR 320 |
| N1=C(SCCC1)Nc1cc(OC(F)F)ccc1 | 7.553 | A VAL 108 A THR 111 A GLN 115 A TRP 124 A VAL 134 A LEU 135 A ASP 138 A ASN 141 A TRP 287 A ILE 290 A CYS 315 A ILE 316 A GLY 319 A TYR 320 |
| N1=C(SCCC1)Nc1c(c(F)cc(c1)C(=O)N)C | 7.541 | A VAL 108 A THR 111 A GLN 115 A TRP 124 A VAL 134 A LEU 135 A ILE 137 A ASP 138 A CYS 210 A ILE 290 A TYR 312 A ILE 316 A GLY 319 A TYR 320 |
| N1=C(SCCC1)NCc1c(O)cccc1 | 7.537 | A VAL 108 A THR 111 A PHE 114 A GLN 115 A TRP 124 A VAL 134 A LEU 135 A ASP 138 A TRP 287 A ILE 290 A CYS 315 A ILE 316 A GLY 319 A TYR 320 |
| N1=C(SCCC1)Nc1c(ccc(c1)C(=O)NC)C | 7.535 | A VAL 108 A THR 111 A GLN 115 A TRP 124 A VAL 134 A LEU 135 A ASP 138 A CYS 210 A SER 211 A TRP 287 A ILE 290 A TYR 312 A CYS 315 A ILE 316 A GLY 319 A TYR 320 |
| N1=C(SCCC1)NCc1cc(N)c(Cl)cc1 | 7.53 | A VAL 108 A THR 111 A PHE 114 A GLN 115 A TRP 124 A VAL 134 A LEU 135 A ASP 138 A TRP 287 A ILE 290 A CYS 315 A ILE 316 A GLY 319 A TYR 320 |
| N1=C(SCCC1)Nc1c(N)cc(Cl)c(Cl)c1 | 7.527 | A VAL 108 A ASP 138 A TYR 139 A MET 142 A LYS 227 A VAL 230 A TRP 287 A ILE 290 A HIS 291 A ILE 294 A TYR 312 A ILE 316 A GLY 319 A TYR 320 |
| N1=C(SCCC1)Nc1c(n2nccc2)cccc1F | 7.518 | A THR 111 A GLN 115 A TRP 124 A VAL 134 A LEU 135 A ASP 138 A MET 142 A TRP 287 A ILE 290 A ILE 294 A TYR 312 A ILE 316 A GLY 319 A TYR 320 |
| N1=C(SCCC1)Nc1c(N2CCCC2)cccc1F | 7.515 | A VAL 108 A THR 111 A GLN 115 A VAL 134 A LEU 135 A ASP 138 A MET 142 A TRP 287 A ILE 290 A ILE 294 A TYR 312 A ILE 316 A GLY 319 A TYR 320 |
| N1=C(SCCC1)Nc1c2[nH]ncc2ccc1 | 7.512 | A VAL 108 A THR 111 A GLN 115 A TRP 124 A VAL 134 A LEU 135 A ASP 138 A TRP 287 A ILE 290 A TYR 312 A ILE 316 A GLY 319 A TYR 320 |
| N1=C(SCCC1)Nc1c(cc(O)c(C(C)C)c1)C | 7.508 | A VAL 108 A GLN 115 A ASP 138 A TYR 139 A ASN 141 A MET 142 A LYS 227 A VAL 230 A TRP 287 A ILE 290 A HIS 291 A ILE 294 A TYR 312 A ILE 316 A GLY 319 A TYR 320 |
| N1=C(SCCC1)Nc1c(N)c(F)cc(F)c1 | 7.504 | A VAL 108 A ASP 138 A ASN 141 A MET 142 A VAL 230 A TRP 287 A ILE 290 A HIS 291 A ILE 294 A TYR 312 A CYS 315 A ILE 316 A GLY 319 A TYR 320 |
| N1=C(SCCC1)N[C@H](c1c(F)c(F)ccc1)C | 7.497 | A VAL 108 A ASP 138 A TYR 139 A MET 142 A LYS 227 A VAL 230 A TRP 287 A ILE 290 A HIS 291 A ILE 294 A TYR 312 A CYS 315 A ILE 316 A GLY 319 A TYR 320 |
| N1=C(SCCC1)Nc1c(F)c(ccc1)C | 7.492 | A VAL 108 A THR 111 A GLN 115 A TRP 124 A VAL 134 A LEU 135 A ASP 138 A TRP 287 A ILE 290 A CYS 315 A ILE 316 A GLY 319 A TYR 320 |
| N1=C(SCCC1)NCc1ccc(Cl)cc1 | 7.481 | A VAL 108 A THR 111 A PHE 114 A GLN 115 A TRP 124 A VAL 134 A LEU 135 A ASP 138 A TRP 287 A ILE 290 A CYS 315 A ILE 316 A GLY 319 A TYR 320 |
| N1=C(SCCC1)Nc1cc(ccc1)C#C | 7.48 | A VAL 108 A THR 111 A PHE 114 A GLN 115 A TRP 124 A VAL 134 A LEU 135 A ASP 138 A ASN 141 A TRP 287 A ILE 290 A CYS 315 A ILE 316 A GLY 319 A TYR 320 |
| N1=C(SCCC1)Nc1c(O)cc(c(Cl)c1)C | 7.475 | A VAL 108 A ASP 138 A TYR 139 A MET 142 A LYS 227 A VAL 230 A TRP 287 A ILE 290 A HIS 291 A ILE 294 A ILE 316 A GLY 319 A TYR 320 |
| N1=C(SCCC1)Nc1c(O)c(cc(c1)C)C | 7.471 | A VAL 108 A THR 111 A GLN 115 A TRP 124 A VAL 134 A LEU 135 A ASP 138 A ASN 141 A TRP 287 A ILE 290 A TYR 312 A ILE 316 A GLY 319 A TYR 320 |
| N1=C(SCCC1)Nc1c(c(ccc1)C(=O)N)C | 7.464 | A VAL 108 A ASP 138 A TYR 139 A ASN 141 A MET 142 A VAL 230 A TRP 287 A ILE 290 A HIS 291 A ILE 294 A TYR 312 A CYS 315 A ILE 316 A GLY 319 A TYR 320 |
| N1=C(SCCC1)Nc1cc2c([nH]cc2)cc1 | 7.461 | A ASP 138 A TYR 139 A MET 142 A LYS 227 A VAL 230 A TRP 287 A ILE 290 A HIS 291 A ILE 294 A ILE 316 A GLY 319 A TYR 320 |
| N1=C(SCCC1)Nc1c(NS(=O)(=O)C)cccc1 | 7.456 | A VAL 108 A THR 111 A GLN 115 A TRP 124 A VAL 134 A LEU 135 A ASP 138 A MET 142 A TRP 287 A ILE 290 A ILE 294 A ILE 316 A GLY 319 A TYR 320 |
| N1=C(SCCC1)Nc1c(F)cc(F)c(F)c1 | 7.449 | A ASP 138 A TYR 139 A MET 142 A VAL 230 A TRP 287 A ILE 290 A ILE 294 A ILE 316 A GLY 319 A TYR 320 |
| N1=C(SCCC1)Nc1c(cccc1N)C(=O)N | 7.448 | A VAL 108 A THR 111 A PHE 114 A GLN 115 A VAL 134 A ASP 138 A TRP 287 A ILE 290 A TYR 312 A ILE 316 A GLY 319 A TYR 320 |
| N1=C(SCCC1)Nc1c(N)cc(cc1)C#N | 7.438 | A VAL 108 A THR 111 A GLN 115 A TRP 124 A VAL 134 A LEU 135 A ASP 138 A TRP 287 A ILE 290 A ILE 316 A GLY 319 A TYR 320 |
| N1=C(SCCC1)Nc1cc(Cl)c(cc1Cl)C | 7.431 | A VAL 108 A ASP 138 A TYR 139 A MET 142 A LYS 227 A VAL 230 A TRP 287 A ILE 290 A ILE 294 A ILE 316 A GLY 319 A TYR 320 |
| N1=C(SCCC1)Nc1cc(c(F)cc1Cl)C | 7.425 | A ASP 138 A TYR 139 A MET 142 A LYS 227 A VAL 230 A TRP 287 A ILE 290 A HIS 291 A ILE 294 A CYS 315 A ILE 316 A GLY 319 A TYR 320 |
| N1=C(SCCC1)Nc1c(N2CCCC2)cccc1 | 7.415 | A THR 111 A GLN 115 A TRP 124 A VAL 134 A LEU 135 A ASP 138 A MET 142 A TRP 287 A ILE 290 A ILE 294 A TYR 312 A ILE 316 A GLY 319 A TYR 320 |
| N1=C(SCCC1)N[C@@H]1CCN(c2c1cccc2)C | 7.415 | A VAL 108 A ASP 138 A TYR 139 A MET 142 A LYS 227 A VAL 230 A TRP 287 A ILE 290 A HIS 291 A ILE 294 A TYR 312 A CYS 315 A ILE 316 A GLY 319 A TYR 320 |
| N1=C(SCCC1)Nc1ccc(nc1O)C | 7.402 | A VAL 108 A THR 111 A PHE 114 A GLN 115 A TRP 124 A VAL 134 A ASP 138 A TRP 287 A ILE 290 A TYR 312 A ILE 316 A GLY 319 A TYR 320 |
| N1=C(SCCC1)Nc1c(c(O)ccc1F)C | 7.38 | A VAL 108 A THR 111 A GLN 115 A VAL 134 A ILE 137 A ASP 138 A TRP 287 A ILE 290 A TYR 312 A ILE 316 A GLY 319 A TYR 320 |
| N1=C(SCCC1)Nc1c(cc(OC)c(F)c1)C | 7.372 | A VAL 108 A THR 111 A PHE 114 A GLN 115 A TRP 124 A VAL 134 A ASP 138 A TRP 287 A ILE 290 A TYR 312 A ILE 316 A GLY 319 A TYR 320 |
| N1=C(SCCC1)N[C@@H](c1cc(cc(c1)C)C)C | 7.372 | A VAL 108 A THR 111 A PHE 114 A GLN 115 A TRP 124 A VAL 134 A ASP 138 A TRP 287 A ILE 290 A ILE 294 A CYS 315 A ILE 316 A GLY 319 A TYR 320 |
| N1=C(SCCC1)N1CCC[C@@H](c2c1cccc2)C | 7.363 | A THR 111 A PHE 114 A GLN 115 A VAL 118 A TRP 124 A VAL 134 A LEU 135 A ASP 138 A CYS 210 A SER 211 A ILE 290 A ILE 294 A TYR 312 A ILE 316 A TYR 320 |
| N1=C(SCCC1)NCc1c(Cl)c(Cl)ccc1 | 7.362 | A VAL 108 A ASP 138 A TYR 139 A MET 142 A LYS 227 A VAL 230 A TRP 287 A ILE 290 A HIS 291 A ILE 294 A ILE 316 A GLY 319 A TYR 320 |
| N1=C(SCCC1)Nc1c2nsnc2ccc1Cl | 7.354 | A VAL 108 A THR 111 A PHE 114 A GLN 115 A VAL 118 A TRP 124 A VAL 134 A LEU 135 A ASP 138 A CYS 210 A TYR 312 A TYR 313 A TYR 320 |
| N1=C(SCCC1)Nc1c(C(C)C)cccc1CC | 7.35 | A ASP 138 A TYR 139 A MET 142 A SER 211 A LEU 212 A MET 226 A LYS 227 A VAL 230 A TRP 287 A ILE 290 A HIS 291 A ILE 294 A GLU 297 A TYR 312 A ILE 316 |
| N1=C(SCCC1)Nc1c(N)c2ncsc2cc1 | 7.348 | A VAL 108 A ASP 138 A TYR 139 A MET 142 A LYS 227 A VAL 230 A TRP 287 A ILE 290 A HIS 291 A ILE 294 A ILE 316 A GLY 319 A TYR 320 |
| N1=C(SCCC1)Nc1cc(N)c(Cl)cc1 | 7.335 | A VAL 108 A ASP 138 A TYR 139 A MET 142 A LYS 227 A VAL 230 A TRP 287 A ILE 290 A HIS 291 A ILE 294 A CYS 315 A ILE 316 A GLY 319 A TYR 320 |
| N1=C(SCCC1)N[C@H](c1c(OC)ccc(c1)C)C | 7.334 | A GLN 115 A ASP 138 A TYR 139 A MET 142 A LYS 227 A VAL 230 A TRP 287 A ILE 290 A HIS 291 A ILE 294 A TYR 312 A CYS 315 A ILE 316 A GLY 319 A TYR 320 |
| N1=C(SCCC1)Nc1c(OC)cc(F)c(F)c1 | 7.323 | A GLN 115 A ASP 138 A TYR 139 A MET 142 A VAL 230 A TRP 287 A ILE 290 A ILE 294 A TYR 312 A ILE 316 A GLY 319 A TYR 320 |
| N1=C(SCCC1)Nc1c(OCC)cc(cc1)C | 7.313 | A VAL 108 A THR 111 A GLN 115 A VAL 134 A LEU 135 A ASP 138 A TRP 287 A ILE 290 A ILE 294 A TYR 312 A CYS 315 A ILE 316 A GLY 319 A TYR 320 |
| N1=C(SCCC1)Nc1c(Cl)c(ccc1)C | 7.309 | A VAL 108 A THR 111 A GLN 115 A TRP 124 A VAL 134 A LEU 135 A ASP 138 A TRP 287 A ILE 290 A CYS 315 A ILE 316 A GLY 319 A TYR 320 |
| N1=C(SCCC1)NC(c1ccccc1)(C)C | 7.309 | A VAL 108 A THR 111 A PHE 114 A GLN 115 A VAL 118 A TRP 124 A VAL 134 A LEU 135 A ASP 138 A CYS 210 A SER 211 A TRP 287 A ILE 316 A TYR 320 |
| N1=C(SCCC1)Nc1c(N)c(ccc1)C#N | 7.304 | A GLN 115 A ASP 138 A TYR 139 A MET 142 A LYS 227 A VAL 230 A TRP 287 A ILE 290 A ILE 294 A ILE 316 A GLY 319 A TYR 320 |
| N1=C(SCCC1)Nc1cc(c(F)cc1)C | 7.304 | A VAL 108 A THR 111 A PHE 114 A GLN 115 A TRP 124 A VAL 134 A LEU 135 A ASP 138 A TRP 287 A ILE 290 A ILE 316 A GLY 319 A TYR 320 |
| N1=C(SCCC1)Nc1c(C(C)C)cccc1C | 7.3 | A VAL 108 A THR 111 A GLN 115 A VAL 134 A ASP 138 A MET 142 A TRP 287 A ILE 290 A TYR 312 A CYS 315 A ILE 316 A GLY 319 A TYR 320 |
| N1=C(SCCC1)N[C@H]1[C@@H](CCc2c1cccc2)C | 7.299 | A VAL 108 A GLN 115 A TRP 124 A VAL 134 A LEU 135 A ASP 138 A TRP 287 A ILE 290 A ILE 294 A TYR 312 A ILE 316 A GLY 319 A TYR 320 |
| N1=C(SCCC1)N[C@@H](c1ccc(cc1)C)CC | 7.299 | A VAL 108 A ASP 138 A TYR 139 A ASN 141 A MET 142 A LYS 227 A VAL 230 A PHE 231 A TRP 287 A ILE 290 A HIS 291 A ILE 294 A TYR 312 A ILE 316 A GLY 319 A TYR 320 |
| N1=C(SCCC1)Nc1c(c(O)c(cc1)C)C | 7.297 | A VAL 108 A THR 111 A GLN 115 A TRP 124 A VAL 134 A LEU 135 A ASP 138 A TRP 287 A ILE 290 A TYR 312 A ILE 316 A GLY 319 A TYR 320 |
| N1=C(SCCC1)Nc1cc(c(cc1)C)C | 7.294 | A VAL 108 A THR 111 A GLN 115 A TRP 124 A VAL 134 A LEU 135 A ASP 138 A ASN 141 A TRP 287 A ILE 290 A TYR 312 A CYS 315 A ILE 316 A GLY 319 A TYR 320 |
| N1=C(SCCC1)Nc1c2c([nH]cc2)ccc1 | 7.294 | A VAL 108 A THR 111 A GLN 115 A VAL 118 A TRP 124 A VAL 134 A LEU 135 A ILE 137 A ASP 138 A CYS 210 A TYR 312 A ILE 316 A TYR 320 |
| N1=C(SCCC1)N[n+]1c(cc(cc1C)C)C | 7.291 | A VAL 108 A THR 111 A PHE 114 A GLN 115 A VAL 118 A TRP 124 A VAL 134 A ILE 137 A ASP 138 A TRP 287 A TYR 312 A ILE 316 A GLY 319 A TYR 320 |
| N1=C(SCCC1)Nc1c(F)c(Cl)ccc1 | 7.287 | A VAL 108 A THR 111 A GLN 115 A TRP 124 A VAL 134 A LEU 135 A ASP 138 A TRP 287 A ILE 290 A ILE 316 A GLY 319 A TYR 320 |
| N1=C(SCCC1)Nc1c(CN(C)C)cc(cc1)C | 7.285 | A VAL 108 A THR 111 A GLN 115 A VAL 134 A ASP 138 A MET 142 A TRP 287 A ILE 290 A ILE 294 A TYR 312 A CYS 315 A ILE 316 A LEU 318 A GLY 319 A TYR 320 |
| N1=C(SCCC1)Nc1c(cc(cc1C)C)C | 7.28 | A VAL 108 A THR 111 A GLN 115 A TRP 124 A VAL 134 A LEU 135 A ILE 137 A ASP 138 A TRP 287 A ILE 290 A TYR 312 A ILE 316 A GLY 319 A TYR 320 |
| N1=C(SCCC1)Nc1c(N)ccc(c1)C | 7.28 | A VAL 108 A ASP 138 A TYR 139 A MET 142 A VAL 230 A TRP 287 A ILE 290 A HIS 291 A ILE 294 A ILE 316 A GLY 319 A TYR 320 |
| N1=C(SCCC1)N1c2c(CCC1)cccc2C | 7.28 | A THR 111 A PHE 114 A GLN 115 A VAL 118 A TRP 124 A VAL 134 A LEU 135 A ASP 138 A CYS 210 A SER 211 A TYR 312 A TYR 320 |
| N1=C(SCCC1)Nc1c(cc2OCOc2c1)C | 7.279 | A VAL 108 A THR 111 A GLN 115 A TRP 124 A VAL 134 A LEU 135 A ASP 138 A CYS 210 A TRP 287 A TYR 312 A ILE 316 A TYR 320 |
| N1=C(SCCC1)Nc1c(c(Cl)c(cc1O)C)C | 7.27 | A VAL 108 A ASP 138 A TYR 139 A MET 142 A LYS 227 A VAL 230 A TRP 287 A ILE 290 A HIS 291 A ILE 294 A ILE 316 A GLY 319 A TYR 320 |
| N1=C(SCCC1)Nc1c(c(OC)c(cc1)C)C | 7.269 | A VAL 108 A ASP 138 A TYR 139 A MET 142 A LYS 227 A VAL 230 A TRP 287 A ILE 290 A HIS 291 A ILE 294 A TYR 312 A ILE 316 A GLY 319 A TYR 320 |
| N1=C(SCCC1)Nc1c(cc(N)cc1)C(F)F | 7.268 | A VAL 108 A THR 111 A GLN 115 A TRP 124 A VAL 134 A LEU 135 A ILE 137 A ASP 138 A ASN 141 A CYS 210 A TRP 287 A TYR 312 A ILE 316 A GLY 319 A TYR 320 |
| N1=C(SCCC1)Nc1c(SC(F)F)cccc1 | 7.253 | A VAL 108 A THR 111 A PHE 114 A GLN 115 A TRP 124 A VAL 134 A LEU 135 A ASP 138 A MET 142 A TRP 287 A ILE 290 A ILE 316 A GLY 319 A TYR 320 |
| N1=C(SCCC1)Nc1c(Cl)c(F)ccc1 | 7.248 | A VAL 108 A THR 111 A GLN 115 A TRP 124 A VAL 134 A LEU 135 A ASP 138 A TRP 287 A ILE 290 A ILE 316 A GLY 319 A TYR 320 |
| N1=C(SCCC1)Nc1c(F)c(ccc1F)C | 7.246 | A VAL 108 A THR 111 A GLN 115 A VAL 118 A TRP 124 A VAL 134 A LEU 135 A ILE 137 A ASP 138 A CYS 210 A TYR 312 A ILE 316 A TYR 320 |
| N1=C(SCCC1)Nc1c(CC)cccc1C | 7.239 | A VAL 108 A THR 111 A GLN 115 A VAL 134 A ASP 138 A ASN 141 A TRP 287 A ILE 290 A TYR 312 A CYS 315 A ILE 316 A GLY 319 A TYR 320 A SER 323 |
| N1=C(SCCC1)Nc1c(N)cccc1F | 7.238 | A VAL 108 A ASP 138 A TYR 139 A MET 142 A VAL 230 A TRP 287 A ILE 290 A ILE 294 A ILE 316 A GLY 319 A TYR 320 |
| N1=C(SCCC1)Nc1cc2OCOc2cc1 | 7.235 | A VAL 108 A ASP 138 A TYR 139 A MET 142 A VAL 230 A TRP 287 A ILE 290 A HIS 291 A ILE 294 A ILE 316 A GLY 319 A TYR 320 |
| N1=C(SCCC1)Nc1c(cccc1N)C | 7.232 | A ASP 138 A TYR 139 A MET 142 A VAL 230 A TRP 287 A ILE 290 A ILE 294 A CYS 315 A ILE 316 A GLY 319 A TYR 320 |
| N1=C(SCCC1)Nc1c(N)cc(cc1)C | 7.227 | A VAL 108 A ASP 138 A TYR 139 A MET 142 A LYS 227 A VAL 230 A TRP 287 A ILE 290 A HIS 291 A ILE 294 A TYR 312 A CYS 315 A ILE 316 A GLY 319 A TYR 320 |
| N1=C(SCCC1)Nc1c(N)cc(F)c(OC)c1 | 7.226 | A VAL 108 A THR 111 A GLN 115 A TRP 124 A VAL 134 A LEU 135 A ASP 138 A ASN 141 A TRP 287 A ILE 290 A ILE 316 A GLY 319 A TYR 320 A SER 323 |
| N1=C(SCCC1)N[C@@H](c1c(F)cccc1F)C | 7.222 | A VAL 108 A THR 111 A PHE 114 A GLN 115 A VAL 118 A TRP 124 A VAL 134 A LEU 135 A ASP 138 A CYS 210 A SER 211 A ILE 316 A TYR 320 |
| N1=C(SCCC1)N1c2c(CN(CC1)C)cccc2 | 7.221 | A THR 111 A PHE 114 A GLN 115 A VAL 118 A TRP 124 A VAL 134 A LEU 135 A ASP 138 A CYS 210 A TYR 312 A ILE 316 A TYR 320 |
| N1=C(SCCC1)Nc1c(c(N)ccc1)C | 7.214 | A VAL 108 A THR 111 A GLN 115 A VAL 134 A ILE 137 A ASP 138 A TRP 287 A ILE 290 A TYR 312 A ILE 316 A GLY 319 A TYR 320 |
| N1=C(SCCC1)Nc1c(cc(cc1Cl)C)C | 7.211 | A VAL 108 A THR 111 A GLN 115 A TRP 124 A VAL 134 A LEU 135 A ILE 137 A ASP 138 A TRP 287 A ILE 290 A TYR 312 A CYS 315 A ILE 316 A GLY 319 A TYR 320 |
| N1=C(SCCC1)Nc1c(cc(OCC)cc1)C | 7.208 | A VAL 108 A THR 111 A GLN 115 A TRP 124 A VAL 134 A LEU 135 A ASP 138 A ASN 141 A CYS 210 A TRP 287 A TYR 312 A ILE 316 A GLY 319 A TYR 320 |
| N1=C(SCCC1)Nc1c(N)cc(Cl)cc1 | 7.205 | A VAL 108 A THR 111 A GLN 115 A TRP 124 A VAL 134 A LEU 135 A ASP 138 A TRP 287 A ILE 290 A ILE 316 A GLY 319 A TYR 320 |
| N1=C(SCCC1)Nc1c(N)c(F)ccc1 | 7.202 | A VAL 108 A THR 111 A GLN 115 A TRP 124 A VAL 134 A LEU 135 A ASP 138 A TRP 287 A ILE 290 A TYR 312 A ILE 316 A GLY 319 A TYR 320 |
| N1=C(SCCC1)N[C@@H](c1c(OC)cccc1)C | 7.2 | A VAL 108 A THR 111 A PHE 114 A GLN 115 A TRP 124 A VAL 134 A LEU 135 A ASP 138 A ASN 141 A TRP 287 A ILE 290 A ILE 316 A GLY 319 A TYR 320 |
| N1=C(SCCC1)N[C@@H]1C2(CCCC1)CCC2 | 7.197 | A VAL 108 A THR 111 A GLN 115 A VAL 134 A LEU 135 A ASP 138 A ASN 141 A TRP 287 A ILE 290 A TYR 312 A ILE 316 A GLY 319 A TYR 320 |
| N1=C(SCCC1)Nc1c(N)ccc(F)c1F | 7.196 | A VAL 108 A ASP 138 A TYR 139 A MET 142 A VAL 230 A TRP 287 A ILE 290 A ILE 294 A ILE 316 A GLY 319 A TYR 320 |
| N1=C(SCCC1)Nc1cc(c(nc1C)C)C | 7.196 | A VAL 108 A THR 111 A PHE 114 A GLN 115 A TRP 124 A VAL 134 A ASP 138 A TRP 287 A ILE 290 A TYR 312 A ILE 316 A GLY 319 A TYR 320 |
| N1=C(SCCC1)NCc1c(OC)cc(C)cc1 | 7.191 | A VAL 108 A THR 111 A PHE 114 A GLN 115 A TRP 124 A VAL 134 A LEU 135 A ASP 138 A TRP 287 A ILE 290 A CYS 315 A ILE 316 A LEU 318 A GLY 319 A TYR 320 |
| N1=C(SCCC1)[C@H]1NCCc2c1cccc2 | 7.188 | A VAL 108 A THR 111 A GLN 115 A TRP 124 A VAL 134 A ASP 138 A TRP 287 A ILE 290 A TYR 312 A ILE 316 A GLY 319 A TYR 320 |
| N1=C(SCCC1)Nc1c(N)cc(F)cc1 | 7.186 | A VAL 108 A ASP 138 A TYR 139 A MET 142 A VAL 230 A TRP 287 A ILE 290 A HIS 291 A ILE 294 A TYR 312 A CYS 315 A ILE 316 A GLY 319 A TYR 320 |
| N1=C(SCCC1)Nc1c(F)cc(N)cc1F | 7.181 | A VAL 108 A THR 111 A GLN 115 A VAL 134 A LEU 135 A ASP 138 A TRP 287 A ILE 290 A TYR 312 A ILE 316 A GLY 319 A TYR 320 |
| N1=C(SCCC1)Nc1cc(cc(Cl)c1)C | 7.169 | A VAL 108 A ASP 138 A TYR 139 A MET 142 A VAL 230 A TRP 287 A ILE 290 A HIS 291 A ILE 294 A ILE 316 A GLY 319 A TYR 320 |
| N1=C(SCCC1)NCc1ccccc1 | 7.166 | A VAL 108 A THR 111 A PHE 114 A GLN 115 A TRP 124 A VAL 134 A LEU 135 A ILE 137 A ASP 138 A TRP 287 A ILE 290 A CYS 315 A ILE 316 A GLY 319 A TYR 320 |
| N1=C(SCCC1)Nc1c(cccc1C)C | 7.161 | A VAL 108 A THR 111 A GLN 115 A VAL 134 A ASP 138 A ASN 141 A TRP 287 A ILE 290 A TYR 312 A CYS 315 A ILE 316 A GLY 319 A TYR 320 |
| N1=C(SCCC1)Nc1c(c(ccc1)C(=O)OC)C | 7.161 | A VAL 108 A ASP 138 A TYR 139 A ASN 141 A TRP 287 A ILE 290 A ILE 294 A TYR 312 A ILE 316 A GLY 319 A TYR 320 A SER 323 |
| N1=C(SCCC1)Nc1c(ccc(c1)C(=O)C)C | 7.151 | A VAL 108 A ASP 138 A TYR 139 A ASN 141 A MET 142 A LYS 227 A VAL 230 A TRP 287 A ILE 290 A HIS 291 A ILE 294 A TYR 312 A ILE 316 A GLY 319 A TYR 320 |
| N1=C(SCCC1)Nc1c(nn(c1N1CCC1)C)C | 7.149 | A VAL 108 A THR 111 A PHE 114 A GLN 115 A TRP 124 A VAL 134 A LEU 135 A ASP 138 A TRP 287 A ILE 290 A ILE 294 A TYR 312 A ILE 316 A GLY 319 A TYR 320 |
| N1=C(SCCC1)Nc1c(cccc1Cl)C(F)(F)F | 7.146 | A VAL 108 A THR 111 A PHE 114 A GLN 115 A TRP 124 A VAL 134 A ASP 138 A TRP 287 A ILE 290 A ILE 294 A TYR 312 A ILE 316 A TYR 320 |
| N1=C(SCCC1)Nc1ncc(Cl)cc1N | 7.146 | A VAL 108 A THR 111 A PHE 114 A GLN 115 A TRP 124 A VAL 134 A ASP 138 A TRP 287 A ILE 290 A TYR 312 A ILE 316 A GLY 319 A TYR 320 |
| N1=C(SCCC1)Nc1c(Cl)cc(Cl)cc1Cl | 7.138 | A VAL 108 A THR 111 A GLN 115 A TRP 124 A VAL 134 A LEU 135 A ILE 137 A ASP 138 A TRP 287 A ILE 290 A ILE 316 A GLY 319 A TYR 320 |
| N1=C(SCCC1)Nc1c(OC(F)F)cccc1 | 7.137 | A VAL 108 A THR 111 A GLN 115 A TRP 124 A VAL 134 A LEU 135 A ASP 138 A MET 142 A TRP 287 A ILE 290 A ILE 316 A GLY 319 A TYR 320 |
| N1=C(SCCC1)Nc1cc(ccc1Cl)C | 7.134 | A VAL 108 A THR 111 A ASP 138 A TYR 139 A MET 142 A VAL 230 A TRP 287 A ILE 290 A ILE 294 A ILE 316 A GLY 319 A TYR 320 |
| N1=C(SCCC1)Nc1c(OC)ccc(c1)C | 7.134 | A VAL 108 A THR 111 A ASP 138 A TYR 139 A TRP 287 A ILE 290 A ILE 294 A TYR 312 A CYS 315 A ILE 316 A GLY 319 A TYR 320 |
| N1=C(SCCC1)Nc1c(ccc(c1)C(=O)N)C | 7.13 | A VAL 108 A THR 111 A GLN 115 A TRP 124 A VAL 134 A LEU 135 A ASP 138 A CYS 210 A ILE 290 A TYR 312 A ILE 316 A GLY 319 A TYR 320 |
| N1=C(SCCC1)Nc1c(Cl)cc(F)cc1Cl | 7.124 | A VAL 108 A THR 111 A GLN 115 A VAL 134 A LEU 135 A ILE 137 A ASP 138 A TRP 287 A ILE 290 A ILE 316 A GLY 319 A TYR 320 |
| N1=C(SCCC1)Nc1c(OC)ccc(F)c1 | 7.119 | A GLN 115 A ASP 138 A TYR 139 A MET 142 A VAL 230 A TRP 287 A ILE 290 A ILE 294 A ILE 316 A GLY 319 A TYR 320 |
| N1=C(SCCC1)Nc1c(OC)cc(cc1)C | 7.117 | A VAL 108 A THR 111 A GLN 115 A VAL 134 A ASP 138 A TRP 287 A ILE 290 A TYR 312 A CYS 315 A ILE 316 A GLY 319 A TYR 320 |
| N1=C(SCCC1)Nc1ccc(F)c(F)c1 | 7.117 | A VAL 108 A THR 111 A GLN 115 A TRP 124 A VAL 134 A LEU 135 A ASP 138 A TRP 287 A ILE 290 A TYR 312 A ILE 316 A GLY 319 A TYR 320 |
| N1=C(SCCC1)Nc1cc(F)c(OC)cc1 | 7.114 | A VAL 108 A ASP 138 A TYR 139 A MET 142 A LYS 227 A VAL 230 A TRP 287 A ILE 290 A HIS 291 A ILE 294 A CYS 315 A ILE 316 A GLY 319 A TYR 320 |
| N1=C(SCCC1)Nc1c2CCOCc2ccc1 | 7.113 | A LEU 135 A ASP 138 A TYR 139 A MET 142 A SER 211 A LEU 212 A LYS 227 A VAL 230 A TRP 287 A ILE 290 A HIS 291 A ILE 294 A TYR 312 A ILE 316 |
| N1=C(SCCC1)Nc1c(Cl)c(Cl)ccc1 | 7.113 | A VAL 108 A THR 111 A GLN 115 A TRP 124 A VAL 134 A LEU 135 A ASP 138 A TRP 287 A ILE 290 A ILE 316 A GLY 319 A TYR 320 |
| N1=C(SCCC1)Nc1c(cc(nc1C)C)C | 7.112 | A VAL 108 A THR 111 A GLN 115 A TRP 124 A VAL 134 A LEU 135 A ILE 137 A ASP 138 A TRP 287 A ILE 290 A TYR 312 A ILE 316 A GLY 319 A TYR 320 |
| N1=C(SCCC1)Nc1c(cccc1F)C | 7.11 | A VAL 108 A THR 111 A GLN 115 A VAL 134 A ASP 138 A ASN 141 A TRP 287 A ILE 290 A TYR 312 A ILE 316 A GLY 319 A TYR 320 |
| N1=C(SCCC1)Nc1c(O)ccc(c1)C | 7.11 | A VAL 108 A THR 111 A GLN 115 A TRP 124 A VAL 134 A LEU 135 A ASP 138 A TRP 287 A ILE 290 A TYR 312 A CYS 315 A ILE 316 A GLY 319 A TYR 320 |
| N1=C(SCCC1)Nc1c(N)ccc(Cl)c1 | 7.109 | A VAL 108 A ASP 138 A TYR 139 A MET 142 A VAL 230 A TRP 287 A ILE 290 A HIS 291 A ILE 294 A ILE 316 A GLY 319 A TYR 320 |
| N1=C(SCCC1)Nc1cc(CC)ccc1O | 7.103 | A VAL 108 A THR 111 A GLN 115 A TRP 124 A VAL 134 A LEU 135 A ASP 138 A CYS 210 A TRP 287 A ILE 316 A GLY 319 A TYR 320 |
| N1=C(SCCC1)Nc1c(cc(Cl)cc1OC)C | 7.101 | A VAL 108 A THR 111 A PHE 114 A GLN 115 A VAL 118 A TRP 124 A VAL 134 A LEU 135 A ASP 138 A GLU 209 A CYS 210 A TYR 312 A TYR 313 A TYR 320 |
| N1=C(SCCC1)Nc1c(N)ccc(F)c1 | 7.099 | A VAL 108 A THR 111 A GLN 115 A TRP 124 A VAL 134 A LEU 135 A ASP 138 A TRP 287 A ILE 290 A TYR 312 A ILE 316 A GLY 319 A TYR 320 |
| N1=C(SCCC1)N[C@H]1CCCOc2c1cccc2 | 7.098 | A VAL 108 A THR 111 A PHE 114 A GLN 115 A VAL 118 A TRP 124 A VAL 134 A ILE 137 A ASP 138 A CYS 210 A TYR 312 A ILE 316 A TYR 320 |
| N1=C(SCCC1)Nc1c(Cl)c(ccc1Cl)C | 7.091 | A VAL 108 A THR 111 A GLN 115 A VAL 118 A TRP 124 A VAL 134 A LEU 135 A ILE 137 A ASP 138 A CYS 210 A TYR 312 A ILE 316 A TYR 320 |
| N1=C(SCCC1)Nc1c(nc(O)nc1O)C | 7.082 | A VAL 108 A THR 111 A PHE 114 A GLN 115 A TRP 124 A VAL 134 A ASP 138 A TRP 287 A ILE 290 A TYR 312 A ILE 316 A GLY 319 A TYR 320 |
| N1=C(SCCC1)Nc1cc(F)ccc1 | 7.082 | A VAL 108 A THR 111 A GLN 115 A TRP 124 A VAL 134 A LEU 135 A ASP 138 A TRP 287 A ILE 290 A ILE 316 A GLY 319 A TYR 320 |
| N1=C(SCCC1)Nc1c(F)cc(cc1)C | 7.078 | A VAL 108 A ASP 138 A TYR 139 A MET 142 A LYS 227 A VAL 230 A TRP 287 A ILE 290 A HIS 291 A ILE 294 A ILE 316 A GLY 319 A TYR 320 |
| N1=C(SCCC1)Nc1c(OCC)ccc(Cl)c1 | 7.078 | A VAL 108 A THR 111 A VAL 134 A ASP 138 A TYR 139 A TRP 287 A ILE 290 A ILE 294 A TYR 312 A ILE 316 A GLY 319 A TYR 320 |
| N1=C(SCCC1)N1Cc2c(OC)cccc2C1 | 7.078 | A GLN 115 A ASP 138 A TYR 139 A MET 142 A LYS 227 A VAL 230 A TRP 287 A ILE 290 A ILE 294 A TYR 312 A ILE 316 A GLY 319 A TYR 320 |
| N1=C(SCCC1)Nc1c(cc(N)cc1C)C | 7.074 | A VAL 108 A THR 111 A GLN 115 A TRP 124 A VAL 134 A LEU 135 A ILE 137 A ASP 138 A TRP 287 A ILE 290 A TYR 312 A ILE 316 A GLY 319 A TYR 320 |
| N1=C(SCCC1)Nc1c(cc(F)cc1)C | 7.07 | A VAL 108 A THR 111 A GLN 115 A VAL 134 A ASP 138 A TRP 287 A ILE 290 A TYR 312 A CYS 315 A ILE 316 A GLY 319 A TYR 320 |
| N1=C(SCCC1)[C@@H](N)c1cc(Cl)ccc1 | 7.069 | A VAL 108 A THR 111 A GLN 115 A VAL 134 A LEU 135 A ILE 137 A ASP 138 A TRP 287 A ILE 290 A TYR 312 A ILE 316 A GLY 319 A TYR 320 |
| N1=C(SCCC1)Nc1c(C(C)(C)C)cccc1 | 7.068 | A LEU 135 A ASP 138 A TYR 139 A MET 142 A SER 211 A LEU 212 A ASP 223 A LYS 227 A VAL 230 A TRP 287 A ILE 290 A HIS 291 A ILE 294 A TYR 312 A ILE 316 |
| N1=C(SCCC1)Nc1c(N)cc(Br)cc1 | 7.066 | A VAL 108 A ASP 138 A TYR 139 A MET 142 A LYS 227 A VAL 230 A TRP 287 A ILE 290 A ILE 294 A TYR 312 A ILE 316 A GLY 319 A TYR 320 |
| N1=C(SCCC1)NC1CCC2(CC1)CC2 | 7.062 | A VAL 108 A THR 111 A PHE 114 A GLN 115 A VAL 118 A TRP 124 A VAL 134 A LEU 135 A ASP 138 A ASN 141 A TRP 287 A ILE 290 A ILE 316 A GLY 319 A TYR 320 |
| N1=C(SCCC1)Nc1c(O)cc(Cl)cc1 | 7.06 | A VAL 108 A THR 111 A GLN 115 A TRP 124 A VAL 134 A LEU 135 A ASP 138 A TRP 287 A ILE 290 A TYR 312 A ILE 316 A GLY 319 A TYR 320 |
| N1=C(SCCC1)Nc1c(O)cc(cc1)C | 7.058 | A VAL 108 A ASP 138 A TYR 139 A MET 142 A LYS 227 A VAL 230 A TRP 287 A ILE 290 A HIS 291 A ILE 294 A ILE 316 A GLY 319 A TYR 320 |
| N1=C(SCCC1)Nc1c(ccc(F)c1)C | 7.056 | A VAL 108 A THR 111 A GLN 115 A VAL 134 A LEU 135 A ASP 138 A TRP 287 A ILE 290 A TYR 312 A ILE 316 A GLY 319 A TYR 320 |
| N1=C(SCCC1)Cc1c2CCOc2ccc1F | 7.056 | A VAL 108 A THR 111 A GLN 115 A VAL 134 A LEU 135 A ASP 138 A TRP 287 A ILE 290 A TYR 312 A ILE 316 A GLY 319 A TYR 320 |
| N1=C(SCCC1)Nc1c2c(sc1)cccc2 | 7.056 | A VAL 108 A THR 111 A PHE 114 A GLN 115 A TRP 124 A VAL 134 A ASP 138 A TRP 287 A ILE 290 A TYR 312 A ILE 316 A GLY 319 A TYR 320 |
| N1=C(SCCC1)Nc1c([nH]nc1C(F)F)C | 7.051 | A VAL 108 A THR 111 A GLN 115 A VAL 118 A TRP 124 A VAL 134 A LEU 135 A ILE 137 A ASP 138 A CYS 210 A TYR 312 A ILE 316 A TYR 320 |
| N1=C(SCCC1)N[C@@H]1CCOc2c1cccc2 | 7.048 | A VAL 108 A THR 111 A GLN 115 A VAL 118 A TRP 124 A VAL 134 A LEU 135 A ILE 137 A ASP 138 A CYS 210 A TYR 312 A ILE 316 A TYR 320 |
| N1=C(SCCC1)Nc1c(O)c(ccc1)C | 7.045 | A THR 111 A PHE 114 A GLN 115 A TRP 124 A VAL 134 A LEU 135 A ASP 138 A TRP 287 A ILE 290 A ILE 316 A GLY 319 A TYR 320 |
| N1=C(SCCC1)N[C@@H]1C(CCc2c1cccc2)(C)C | 7.043 | A VAL 108 A THR 111 A PHE 114 A GLN 115 A VAL 118 A TRP 124 A VAL 134 A LEU 135 A ASP 138 A TYR 312 A ILE 316 A TYR 320 |
| N1=C(SCCC1)Nc1c(N)c(Cl)ccc1 | 7.04 | A VAL 108 A THR 111 A GLN 115 A TRP 124 A VAL 134 A LEU 135 A ASP 138 A TRP 287 A ILE 290 A TYR 312 A ILE 316 A GLY 319 A TYR 320 |
| N1=C(SCCC1)Nc1cc(Cl)cnc1O | 7.039 | A VAL 108 A THR 111 A PHE 114 A GLN 115 A VAL 134 A ASP 138 A TRP 287 A ILE 290 A TYR 312 A ILE 316 A GLY 319 A TYR 320 |
| N1=C(SCCC1)Nc1cc(Br)cnc1O | 7.034 | A VAL 108 A THR 111 A PHE 114 A GLN 115 A TRP 124 A VAL 134 A ASP 138 A TRP 287 A ILE 290 A TYR 312 A ILE 316 A GLY 319 A TYR 320 |
| N1=C(SCCC1)Nc1c2nc[nH]c2ccc1 | 7.034 | A VAL 108 A THR 111 A GLN 115 A VAL 118 A TRP 124 A VAL 134 A LEU 135 A ILE 137 A ASP 138 A CYS 210 A TYR 312 A ILE 316 A TYR 320 |
| N1=C(SCCC1)Nc1c(F)cccc1F | 7.031 | A VAL 108 A THR 111 A GLN 115 A VAL 134 A LEU 135 A ASP 138 A TRP 287 A ILE 290 A TYR 312 A ILE 316 A GLY 319 A TYR 320 |
| N1=C(SCCC1)Nc1ccc(F)cc1Cl | 7.03 | A VAL 108 A ASP 138 A TYR 139 A MET 142 A VAL 230 A TRP 287 A ILE 290 A HIS 291 A ILE 294 A ILE 316 A GLY 319 A TYR 320 |
| N1=C(SCCC1)Nc1cc(cc(Br)c1)C | 7.03 | A VAL 108 A ASP 138 A TYR 139 A MET 142 A LYS 227 A VAL 230 A TRP 287 A ILE 290 A HIS 291 A ILE 294 A ILE 316 A GLY 319 A TYR 320 |
| N1=C(SCCC1)C1Oc2c(O1)cccc2 | 7.028 | A VAL 108 A THR 111 A GLN 115 A VAL 134 A LEU 135 A ASP 138 A TRP 287 A ILE 290 A TYR 312 A ILE 316 A GLY 319 A TYR 320 |
| N1=C(SCCC1)Nc1c2OCOc2ccc1Cl | 7.026 | A LEU 135 A ASP 138 A TYR 139 A MET 142 A SER 211 A LEU 212 A TYR 219 A ASP 223 A LYS 227 A VAL 230 A TRP 287 A ILE 290 A HIS 291 A ILE 294 A ILE 316 |
| N1=C(SCCC1)Nc1cc(C)ccc1C | 7.019 | A VAL 108 A THR 111 A GLN 115 A VAL 118 A TRP 124 A VAL 134 A LEU 135 A ILE 137 A ASP 138 A CYS 210 A ILE 316 A TYR 320 |
| N1=C(SCCC1)Nc1c(O)cc(N)cc1 | 7.016 | A VAL 108 A THR 111 A GLN 115 A VAL 134 A ASP 138 A TRP 287 A ILE 290 A TYR 312 A CYS 315 A ILE 316 A GLY 319 A TYR 320 |
| N1=C(SCCC1)Nc1c(ccc(N)c1)C | 7.016 | A VAL 108 A THR 111 A GLN 115 A VAL 134 A LEU 135 A ILE 137 A ASP 138 A TRP 287 A ILE 290 A TYR 312 A ILE 316 A GLY 319 A TYR 320 |
| N1=C(SCCC1)Nc1c(ccc(O)c1)C | 7.012 | A VAL 108 A THR 111 A GLN 115 A VAL 134 A ILE 137 A ASP 138 A TRP 287 A ILE 290 A TYR 312 A ILE 316 A GLY 319 A TYR 320 |
| N1=C(SCCC1)Nc1c(ccc(c1)C#N)C | 7.011 | A VAL 108 A THR 111 A GLN 115 A TRP 124 A VAL 134 A LEU 135 A ILE 137 A ASP 138 A CYS 210 A SER 211 A TYR 312 A ILE 316 A GLY 319 A TYR 320 |
| N1=C(SCCC1)Nc1ccc(cc1)C | 7.011 | A VAL 108 A THR 111 A GLN 115 A TRP 124 A VAL 134 A LEU 135 A ASP 138 A TRP 287 A ILE 290 A TYR 312 A CYS 315 A ILE 316 A GLY 319 A TYR 320 |
| N1=C(SCCC1)Nc1c(cc(OC)cc1)C | 7.01 | A VAL 108 A THR 111 A GLN 115 A TRP 124 A VAL 134 A LEU 135 A ASP 138 A TRP 287 A ILE 290 A TYR 312 A ILE 316 A GLY 319 A TYR 320 |
| N1=C(SCCC1)Nc1cc(Cl)cc(Cl)c1 | 7.007 | A VAL 108 A ASP 138 A TYR 139 A MET 142 A VAL 230 A TRP 287 A ILE 290 A HIS 291 A ILE 294 A ILE 316 A GLY 319 A TYR 320 |
| N1=C(SCCC1)Nc1c(N)cccc1Cl | 7.006 | A ASP 138 A TYR 139 A MET 142 A VAL 230 A TRP 287 A ILE 290 A ILE 294 A ILE 316 A GLY 319 A TYR 320 |
| N1=C(SCCC1)N1CCCc2c1c(F)ccc2 | 7.005 | A THR 111 A PHE 114 A GLN 115 A VAL 118 A TRP 124 A VAL 134 A LEU 135 A ASP 138 A TYR 139 A CYS 210 A SER 211 A TYR 312 A TYR 320 |
| N1=C(SCCC1)Nc1c(cc(N(C)C)cc1)C | 7.002 | A VAL 108 A THR 111 A GLN 115 A TRP 124 A VAL 134 A LEU 135 A ASP 138 A ASN 141 A CYS 210 A TRP 287 A TYR 312 A ILE 316 A GLY 319 A TYR 320 |
| N1=C(SCCC1)Nc1c2ncoc2ccc1 | 7 | A ASP 138 A TYR 139 A MET 142 A LYS 227 A VAL 230 A TRP 287 A ILE 290 A HIS 291 A ILE 294 A ILE 316 A GLY 319 A TYR 320 |
| N1=C(SCCC1)N1CCCc2c1c(O)ccc2 | 6.999 | A LEU 135 A ASP 138 A TYR 139 A MET 142 A SER 211 A LEU 212 A ASP 223 A LYS 227 A VAL 230 A PHE 231 A TRP 287 A ILE 290 A HIS 291 A ILE 294 A ILE 316 |
| N1=C(SCCC1)Nc1c([C@H](O)C)cccc1 | 6.998 | A THR 111 A GLN 115 A TRP 124 A VAL 134 A LEU 135 A ASP 138 A MET 142 A TRP 287 A ILE 290 A ILE 294 A ILE 316 A GLY 319 A TYR 320 |
| N1=C(SCCC1)Nc1c([nH+]ccc1)N | 6.997 | A VAL 108 A THR 111 A GLN 115 A TRP 124 A VAL 134 A ILE 137 A ASP 138 A ASN 141 A TRP 287 A ILE 316 A GLY 319 A TYR 320 |
| N1=C(SCCC1)Nc1c(N)cc(OC)cc1 | 6.995 | A VAL 108 A THR 111 A GLN 115 A TRP 124 A VAL 134 A LEU 135 A ASP 138 A TRP 287 A ILE 290 A CYS 315 A ILE 316 A GLY 319 A TYR 320 |
| N1=C(SCCC1)Nc1c(cc(Cl)cc1)C | 6.992 | A VAL 108 A THR 111 A GLN 115 A TRP 124 A VAL 134 A LEU 135 A ASP 138 A TRP 287 A ILE 290 A TYR 312 A ILE 316 A GLY 319 A TYR 320 |
| N1=C(SCCC1)Nc1c(c(Cl)ccc1)C | 6.972 | A VAL 108 A ASP 138 A TYR 139 A MET 142 A LYS 227 A VAL 230 A TRP 287 A ILE 290 A HIS 291 A ILE 294 A TYR 312 A ILE 316 A GLY 319 A TYR 320 |
| N1=C(SCCC1)Nc1c(F)cc(F)cc1Cl | 6.971 | A VAL 108 A THR 111 A GLN 115 A VAL 118 A TRP 124 A VAL 134 A LEU 135 A ILE 137 A ASP 138 A CYS 210 A TYR 312 A ILE 316 A TYR 320 |
| N1=C(SCCC1)Nc1c(c(F)ccc1)C | 6.971 | A VAL 108 A THR 111 A GLN 115 A VAL 134 A ASP 138 A TRP 287 A ILE 290 A TYR 312 A ILE 316 A GLY 319 A TYR 320 |
| N1=C(SCCC1)Nc1c(OC)cc(F)cc1 | 6.965 | A VAL 108 A THR 111 A GLN 115 A VAL 134 A ASP 138 A TRP 287 A ILE 290 A TYR 312 A CYS 315 A ILE 316 A GLY 319 A TYR 320 |
| N1=C(SCCC1)N1CC[C@H](c2c1c(O)ccc2)C | 6.965 | A VAL 108 A THR 111 A GLN 115 A TRP 124 A VAL 134 A LEU 135 A ASP 138 A MET 142 A TRP 287 A ILE 290 A TYR 312 A ILE 316 A GLY 319 A TYR 320 |
| N1=C(SCCC1)Nc1c(N)ccc(OC)c1 | 6.965 | A VAL 108 A ASP 138 A TYR 139 A MET 142 A VAL 230 A TRP 287 A ILE 290 A HIS 291 A ILE 294 A ILE 316 A GLY 319 A TYR 320 |
| N1=C(SCCC1)Nc1c(CC)cccc1CC | 6.963 | A VAL 108 A THR 111 A GLN 115 A VAL 134 A ASP 138 A ASN 141 A MET 142 A TRP 287 A ILE 290 A ILE 294 A TYR 312 A CYS 315 A ILE 316 A GLY 319 A TYR 320 A SER 323 |
| N1=C(SCCC1)N[C@@H]1CCSc2c1cccc2 | 6.962 | A VAL 108 A ASP 138 A TYR 139 A MET 142 A TRP 287 A ILE 290 A HIS 291 A ILE 294 A TYR 312 A CYS 315 A ILE 316 A GLY 319 A TYR 320 |
| N1=C(SCCC1)Nc1c2n(ncc2)ccc1 | 6.956 | A VAL 108 A THR 111 A GLN 115 A VAL 134 A LEU 135 A ASP 138 A TRP 287 A ILE 290 A TYR 312 A ILE 316 A GLY 319 A TYR 320 |
| N1=C(SCCC1)N[C@@H](c1c(n(nc1)C)C)C | 6.955 | A VAL 108 A THR 111 A PHE 114 A GLN 115 A TRP 124 A VAL 134 A ASP 138 A TRP 287 A ILE 290 A CYS 315 A ILE 316 A GLY 319 A TYR 320 |
| N1=C(SCCC1)Nc1c(cccc1Cl)C | 6.949 | A VAL 108 A THR 111 A GLN 115 A VAL 134 A ASP 138 A TRP 287 A ILE 290 A TYR 312 A CYS 315 A ILE 316 A GLY 319 A TYR 320 |
| N1=C(SCCC1)N1[C@@H](c2c(C1)cccc2)C | 6.948 | A VAL 108 A THR 111 A PHE 114 A GLN 115 A TRP 124 A VAL 134 A ILE 137 A ASP 138 A TRP 287 A ILE 290 A TYR 312 A ILE 316 A GLY 319 A TYR 320 |
| N1=C(SCCC1)Nc1c(ccc(OC)c1)C | 6.947 | A GLN 115 A ASP 138 A TYR 139 A MET 142 A VAL 230 A TRP 287 A ILE 290 A ILE 294 A TYR 312 A CYS 315 A ILE 316 A GLY 319 A TYR 320 |
| N1=C(SCCC1)Nc1c(cc(Br)cc1)C | 6.945 | A VAL 108 A THR 111 A GLN 115 A TRP 124 A VAL 134 A LEU 135 A ASP 138 A TRP 287 A ILE 290 A TYR 312 A ILE 316 A GLY 319 A TYR 320 |
| N1=C(SCCC1)[C@@H](N)c1cc(F)ccc1 | 6.944 | A VAL 108 A THR 111 A GLN 115 A VAL 134 A LEU 135 A ILE 137 A ASP 138 A TRP 287 A ILE 290 A TYR 312 A ILE 316 A GLY 319 A TYR 320 |
| N1=C(SCCC1)Nc1c(Cl)c(Cl)c(Cl)cc1 | 6.942 | A VAL 108 A ASP 138 A TYR 139 A MET 142 A LYS 227 A VAL 230 A TRP 287 A ILE 290 A HIS 291 A ILE 294 A ILE 316 A GLY 319 A TYR 320 |
| N1=C(SCCC1)Nc1cc(N)ccc1Cl | 6.941 | A VAL 108 A THR 111 A ASP 138 A TYR 139 A MET 142 A VAL 230 A TRP 287 A ILE 290 A ILE 294 A ILE 316 A GLY 319 A TYR 320 |
| N1=C(SCCC1)Nc1cc(N)ccc1 | 6.94 | A VAL 108 A THR 111 A GLN 115 A VAL 134 A LEU 135 A ASP 138 A ASN 141 A TRP 287 A TYR 312 A ILE 316 A GLY 319 A TYR 320 A SER 323 |
| N1=C(SCCC1)Nc1c(O)ccc(Cl)c1 | 6.928 | A VAL 108 A ASP 138 A TYR 139 A MET 142 A VAL 230 A TRP 287 A ILE 290 A HIS 291 A ILE 294 A ILE 316 A GLY 319 A TYR 320 |
| N1=C(SCCC1)Nc1c(c(Br)ccc1)C | 6.924 | A VAL 108 A THR 111 A GLN 115 A VAL 134 A LEU 135 A ASP 138 A TRP 287 A ILE 290 A TYR 312 A ILE 316 A GLY 319 A TYR 320 |
| N1=C(SCCC1)Nc1c(OCC)cc(F)cc1 | 6.918 | A VAL 108 A THR 111 A GLN 115 A ASP 138 A TYR 139 A MET 142 A VAL 230 A TRP 287 A ILE 290 A ILE 294 A ILE 316 A GLY 319 A TYR 320 |
| N1=C(SCCC1)Nc1c(OC)cc(OC)cc1Cl | 6.91 | A VAL 108 A THR 111 A PHE 114 A GLN 115 A TRP 124 A VAL 134 A ASP 138 A TRP 287 A ILE 290 A TYR 312 A ILE 316 A GLY 319 A TYR 320 |
| N1=C(SCCC1)N1CCCCc2c1cccc2 | 6.91 | A ASP 138 A TYR 139 A MET 142 A MET 226 A LYS 227 A VAL 230 A TRP 287 A ILE 290 A HIS 291 A ILE 294 A TYR 312 A ILE 316 |
| N1=C(SCCC1)Nc1c(CN(C)C)cccc1 | 6.908 | A VAL 108 A THR 111 A GLN 115 A VAL 134 A ASP 138 A MET 142 A TRP 287 A ILE 290 A ILE 294 A TYR 312 A CYS 315 A ILE 316 A GLY 319 A TYR 320 |
| N1=C(SCCC1)Nc1cc(OC)c(N)cc1 | 6.908 | A VAL 108 A THR 111 A GLN 115 A VAL 118 A TRP 124 A VAL 134 A LEU 135 A ILE 137 A ASP 138 A CYS 210 A TYR 312 A ILE 316 A TYR 320 |
| N1=C(SCCC1)N[C@H](c1c(CC)cncc1)C | 6.892 | A VAL 108 A THR 111 A PHE 114 A GLN 115 A TRP 124 A VAL 134 A LEU 135 A ASP 138 A ASN 141 A TRP 287 A ILE 290 A TYR 312 A ILE 316 A GLY 319 A TYR 320 |
| N1=C(SCCC1)N1c2c(CC1)cccc2C | 6.89 | A VAL 108 A THR 111 A GLN 115 A VAL 134 A ASP 138 A TRP 287 A ILE 290 A TYR 312 A ILE 316 A GLY 319 A TYR 320 |
| N1=C(SCCC1)Nc1ccc(Cl)cc1Cl | 6.883 | A VAL 108 A THR 111 A GLN 115 A TRP 124 A VAL 134 A LEU 135 A ASP 138 A TRP 287 A ILE 290 A TYR 312 A ILE 316 A GLY 319 A TYR 320 |
| N1=C(SCCC1)Nc1cc(F)ccc1Cl | 6.883 | A VAL 108 A THR 111 A GLN 115 A VAL 134 A ASP 138 A TRP 287 A ILE 290 A TYR 312 A ILE 316 A GLY 319 A TYR 320 |
| N1=C(SCCC1)Nc1c(O)cc(O)cc1 | 6.881 | A VAL 108 A THR 111 A GLN 115 A VAL 134 A LEU 135 A ASP 138 A TRP 287 A ILE 290 A TYR 312 A CYS 315 A ILE 316 A GLY 319 A TYR 320 |
| N1=C(SCCC1)N1CC[C@H](O)c2c1cccc2 | 6.875 | A THR 111 A PHE 114 A GLN 115 A VAL 118 A TRP 124 A VAL 134 A LEU 135 A ASP 138 A ILE 294 A TYR 312 A ILE 316 A TYR 320 |
| N1=C(SCCC1)NCc1c(Br)cccc1 | 6.873 | A VAL 108 A ASP 138 A TYR 139 A MET 142 A LYS 227 A VAL 230 A TRP 287 A ILE 290 A HIS 291 A ILE 294 A ILE 316 A GLY 319 A TYR 320 |
| N1=C(SCCC1)Nc1cc(ccc1F)C | 6.869 | A VAL 108 A THR 111 A PHE 114 A VAL 134 A LEU 135 A ILE 137 A ASP 138 A TRP 287 A ILE 290 A TYR 312 A CYS 315 A ILE 316 A GLY 319 A TYR 320 |
| N1=C(SCCC1)Cc1c(cc(cc1C)C)C | 6.867 | A VAL 108 A THR 111 A PHE 114 A GLN 115 A VAL 118 A TRP 124 A VAL 134 A LEU 135 A ASP 138 A CYS 210 A ILE 316 A TYR 320 |
| N1=C(SCCC1)Nc1c(CS(=O)(=O)C)cccc1 | 6.866 | A THR 111 A PHE 114 A GLN 115 A VAL 118 A TRP 124 A VAL 134 A ASP 138 A GLU 209 A CYS 210 A TYR 312 A ILE 316 A TYR 320 |
| N1=C(SCCC1)Nc1ccc(F)cc1 | 6.862 | A VAL 108 A THR 111 A GLN 115 A TRP 124 A VAL 134 A LEU 135 A ASP 138 A TRP 287 A ILE 290 A TYR 312 A ILE 316 A GLY 319 A TYR 320 |
| N1=C(SCCC1)Nc1c(N)c(OCC)ccc1 | 6.859 | A VAL 108 A THR 111 A GLN 115 A TRP 124 A VAL 134 A LEU 135 A ASP 138 A MET 142 A TRP 287 A ILE 290 A ILE 294 A ILE 316 A GLY 319 A TYR 320 |
| N1=C(SCCC1)Nc1cc(O)ccc1F | 6.858 | A VAL 108 A THR 111 A VAL 134 A LEU 135 A ASP 138 A TRP 287 A ILE 290 A TYR 312 A CYS 315 A ILE 316 A GLY 319 A TYR 320 |
| N1=C(SCCC1)Nc1c(OCC)cccc1 | 6.856 | A VAL 108 A THR 111 A GLN 115 A VAL 134 A ASP 138 A TRP 287 A ILE 290 A ILE 294 A TYR 312 A ILE 316 A GLY 319 A TYR 320 |
| N1=C(SCCC1)Nc1c(ccc(Cl)c1)C | 6.85 | A VAL 108 A THR 111 A GLN 115 A VAL 118 A TRP 124 A VAL 134 A LEU 135 A ILE 137 A ASP 138 A CYS 210 A ILE 316 A TYR 320 |
| N1=C(SCCC1)Nc1c(cc(O)cc1)C | 6.848 | A VAL 108 A GLN 115 A ASP 138 A TYR 139 A MET 142 A VAL 230 A TRP 287 A ILE 290 A HIS 291 A ILE 294 A TYR 312 A ILE 316 A GLY 319 A TYR 320 |
| N1=C(SCCC1)Nc1cc(NC)ccc1 | 6.848 | A VAL 108 A THR 111 A GLN 115 A TRP 124 A VAL 134 A LEU 135 A ASP 138 A TRP 287 A ILE 290 A ILE 294 A TYR 312 A ILE 316 A GLY 319 A TYR 320 |
| N1=C(SCCC1)Nc1c(OC(C)C)cccc1 | 6.846 | A VAL 108 A THR 111 A GLN 115 A VAL 134 A ASP 138 A MET 142 A TRP 287 A ILE 290 A ILE 294 A TYR 312 A CYS 315 A ILE 316 A GLY 319 A TYR 320 |
| N1=C(SCCC1)Nc1c(C(C)(C)C)cccc1C | 6.845 | A LEU 135 A ASP 138 A TYR 139 A MET 142 A LEU 212 A MET 226 A LYS 227 A VAL 230 A TRP 287 A ILE 290 A HIS 291 A ILE 294 A GLU 297 A TYR 312 A ILE 316 |
| N1=C(SCCC1)Nc1ccc(F)cc1Br | 6.842 | A VAL 108 A THR 111 A GLN 115 A VAL 134 A ILE 137 A ASP 138 A TRP 287 A ILE 290 A ILE 316 A GLY 319 A TYR 320 |
| N1=C(SCCC1)Nc1c(ncc(F)c1)CC | 6.839 | A VAL 108 A THR 111 A GLN 115 A VAL 134 A LEU 135 A ASP 138 A TRP 287 A ILE 290 A TYR 312 A CYS 315 A ILE 316 A GLY 319 A TYR 320 |
| N1=C(SCCC1)Nc1c2sccc2ccc1 | 6.837 | A VAL 108 A THR 111 A GLN 115 A VAL 134 A ASP 138 A TRP 287 A ILE 290 A ILE 294 A TYR 312 A ILE 316 A GLY 319 A TYR 320 |
| N1=C(SCCC1)Nc1c(Br)c(ccc1)C | 6.831 | A VAL 108 A THR 111 A GLN 115 A VAL 134 A LEU 135 A ILE 137 A ASP 138 A TRP 287 A ILE 290 A ILE 316 A GLY 319 A TYR 320 |
| N1=C(SCCC1)Nc1c(F)cc(O)cc1 | 6.828 | A VAL 108 A THR 111 A GLN 115 A VAL 134 A LEU 135 A ASP 138 A TRP 287 A ILE 290 A TYR 312 A ILE 316 A GLY 319 A TYR 320 |
| N1=C(SCCC1)N[C@H](c1c(F)cccc1)C | 6.821 | A VAL 108 A THR 111 A PHE 114 A GLN 115 A VAL 118 A TRP 124 A VAL 134 A LEU 135 A ASP 138 A CYS 210 A ILE 316 A TYR 320 |
| N1=C(SCCC1)Nc1c(OC)cc(Cl)cc1 | 6.819 | A VAL 108 A THR 111 A GLN 115 A VAL 134 A ASP 138 A TRP 287 A ILE 290 A TYR 312 A CYS 315 A ILE 316 A GLY 319 A TYR 320 |
| N1=C(SCCC1)Oc1c([C@@H](N)C)cccc1 | 6.819 | A VAL 108 A THR 111 A GLN 115 A VAL 134 A LEU 135 A ILE 137 A ASP 138 A ASN 141 A TRP 287 A ILE 290 A ILE 316 A GLY 319 A TYR 320 |
| N1=C(SCCC1)Nc1c(CN)cccc1 | 6.811 | A VAL 108 A THR 111 A GLN 115 A VAL 134 A ILE 137 A ASP 138 A TRP 287 A ILE 290 A TYR 312 A ILE 316 A GLY 319 A TYR 320 |
| N1=C(SCCC1)Nc1c(Cl)cccc1Cl | 6.808 | A VAL 108 A THR 111 A GLN 115 A VAL 134 A ILE 137 A ASP 138 A TRP 287 A ILE 290 A ILE 316 A GLY 319 A TYR 320 |
| N1=C(SCCC1)Nc1c(Br)cc(cc1)C | 6.806 | A VAL 108 A THR 111 A GLN 115 A TRP 124 A VAL 134 A LEU 135 A ASP 138 A ASN 141 A CYS 210 A TRP 287 A TYR 312 A ILE 316 A GLY 319 A TYR 320 |
| N1=C(SCCC1)Nc1c([C@H](CC)C)cccc1 | 6.804 | A LEU 135 A ASP 138 A TYR 139 A MET 142 A SER 211 A LYS 227 A VAL 230 A TRP 287 A ILE 290 A HIS 291 A ILE 294 A TYR 312 A ILE 316 |
| N1=C(SCCC1)Nc1cc(Cl)c(N)cc1 | 6.8 | A VAL 108 A ASP 138 A TYR 139 A MET 142 A LYS 227 A VAL 230 A TRP 287 A ILE 290 A HIS 291 A ILE 294 A ILE 316 A GLY 319 A TYR 320 |
| N1=C(SCCC1)Nc1c(ccc(Br)c1)C | 6.796 | A VAL 108 A THR 111 A GLN 115 A VAL 118 A TRP 124 A VAL 134 A LEU 135 A ILE 137 A ASP 138 A CYS 210 A ILE 316 A TYR 320 |
| N1=C(SCCC1)Nc1ccc(OC)nc1OC | 6.796 | A VAL 108 A THR 111 A PHE 114 A GLN 115 A TRP 124 A VAL 134 A ILE 137 A ASP 138 A TRP 287 A ILE 290 A TYR 312 A ILE 316 A GLY 319 A TYR 320 |
| N1=C(SCCC1)Nc1c(cc(N)cc1)C | 6.792 | A VAL 108 A THR 111 A GLN 115 A VAL 118 A TRP 124 A VAL 134 A LEU 135 A ILE 137 A ASP 138 A CYS 210 A TYR 312 A ILE 316 A TYR 320 |
| N1=C(SCCC1)Nc1c(F)cccc1 | 6.79 | A ASP 138 A TYR 139 A MET 142 A VAL 230 A TRP 287 A ILE 290 A ILE 294 A ILE 316 A GLY 319 A TYR 320 |
| N1=C(SCCC1)Nc1c(OC)cc(OC)cc1 | 6.78 | A VAL 108 A THR 111 A PHE 114 A GLN 115 A TRP 124 A VAL 134 A ILE 137 A ASP 138 A TRP 287 A ILE 290 A TYR 312 A ILE 316 A GLY 319 A TYR 320 |
| N1=C(SCCC1)Nc1c(ccnc1)C(F)(F)F | 6.777 | A VAL 108 A THR 111 A GLN 115 A TRP 124 A VAL 134 A LEU 135 A ASP 138 A TRP 287 A ILE 290 A TYR 312 A ILE 316 A GLY 319 A TYR 320 |
| N1=C(SCCC1)Oc1c(CN)c(Cl)ccc1 | 6.772 | A VAL 108 A THR 111 A GLN 115 A VAL 134 A LEU 135 A ILE 137 A ASP 138 A TRP 287 A ILE 290 A ILE 316 A GLY 319 A TYR 320 |
| N1=C(SCCC1)Nc1c(F)cc(Cl)cc1 | 6.767 | A ASP 138 A TYR 139 A MET 142 A LYS 227 A VAL 230 A TRP 287 A ILE 290 A HIS 291 A ILE 294 A ILE 316 A GLY 319 A TYR 320 |
| N1=C(SCCC1)N[C@@H](c1c(nn(c1C)C)C)C | 6.753 | A VAL 108 A THR 111 A PHE 114 A GLN 115 A VAL 118 A TRP 124 A VAL 134 A LEU 135 A ASP 138 A CYS 210 A TRP 287 A ILE 316 A TYR 320 |
| N1=C(SCCC1)Nc1c(Cl)cccc1F | 6.75 | A VAL 108 A THR 111 A GLN 115 A VAL 134 A LEU 135 A ASP 138 A TRP 287 A ILE 290 A TYR 312 A ILE 316 A GLY 319 A TYR 320 |
| N1=C(SCCC1)N(c1c(cc(cc1)C)C)C | 6.749 | A VAL 108 A THR 111 A PHE 114 A GLN 115 A VAL 118 A TRP 124 A VAL 134 A LEU 135 A ILE 137 A ASP 138 A CYS 210 A TYR 312 A ILE 316 A TYR 320 |
| N1=C(SCCC1)Nc1c(OC)cc(OC)c(Cl)c1 | 6.747 | A VAL 108 A THR 111 A PHE 114 A GLN 115 A TRP 124 A VAL 134 A ILE 137 A ASP 138 A TRP 287 A ILE 290 A TYR 312 A ILE 316 A GLY 319 A TYR 320 |
| N1=C(SCCC1)Nc1ccc(Br)cc1F | 6.744 | A VAL 108 A ASP 138 A TYR 139 A MET 142 A LYS 227 A VAL 230 A TRP 287 A ILE 290 A ILE 294 A ILE 316 A GLY 319 A TYR 320 |
| N1=C(SCCC1)Nc1c(O)ccc(F)c1 | 6.742 | A VAL 108 A THR 111 A VAL 134 A LEU 135 A ASP 138 A TRP 287 A ILE 290 A TYR 312 A ILE 316 A GLY 319 A TYR 320 |
| N1=C(SCCC1)C1Sc2c(S1)cccc2 | 6.741 | A VAL 108 A THR 111 A PHE 114 A GLN 115 A VAL 118 A TRP 124 A VAL 134 A ASP 138 A ASN 141 A TRP 287 A ILE 316 A GLY 319 A TYR 320 |
| N1=C(SCCC1)Nc1c(C(C)C)cccc1 | 6.738 | A VAL 108 A THR 111 A GLN 115 A VAL 134 A ASP 138 A MET 142 A TRP 287 A ILE 290 A ILE 294 A TYR 312 A CYS 315 A ILE 316 A GLY 319 A TYR 320 |
| N1=C(SCCC1)Nc1c(c(Cl)cnc1)C | 6.738 | A VAL 108 A THR 111 A GLN 115 A VAL 134 A ILE 137 A ASP 138 A TRP 287 A ILE 290 A TYR 312 A ILE 316 A GLY 319 A TYR 320 |
| N1=C(SCCC1)Nc1c(N)cccc1 | 6.736 | A VAL 108 A THR 111 A GLN 115 A TRP 124 A VAL 134 A LEU 135 A ASP 138 A TRP 287 A ILE 290 A TYR 312 A ILE 316 A GLY 319 A TYR 320 |
| N1=C(SCCC1)Nc1cccnc1O | 6.73 | A VAL 108 A THR 111 A PHE 114 A GLN 115 A VAL 134 A ASP 138 A TRP 287 A ILE 290 A TYR 312 A ILE 316 A GLY 319 A TYR 320 |
| N1=C(SCCC1)Nc1c(cccc1OC)C | 6.728 | A VAL 108 A THR 111 A PHE 114 A GLN 115 A VAL 118 A TRP 124 A VAL 134 A LEU 135 A ASP 138 A CYS 210 A TYR 312 A TYR 313 A TYR 320 |
| N1=C(SCCC1)Nc1c(N)ccc(Br)c1 | 6.728 | A VAL 108 A THR 111 A GLN 115 A VAL 134 A LEU 135 A ASP 138 A TRP 287 A ILE 290 A TYR 312 A CYS 315 A ILE 316 A GLY 319 A TYR 320 |
| N1=C(SCCC1)Nc1c(F)cc(N)cc1 | 6.723 | A ASP 138 A TYR 139 A MET 142 A LYS 227 A VAL 230 A TRP 287 A ILE 290 A HIS 291 A ILE 294 A ILE 316 A GLY 319 A TYR 320 |
| N1=C(SCCC1)Nc1c(OC(C)(C)C)cccc1 | 6.713 | A VAL 108 A THR 111 A GLN 115 A TRP 124 A VAL 134 A LEU 135 A ASP 138 A MET 142 A TRP 287 A ILE 290 A ILE 294 A ILE 316 A GLY 319 A TYR 320 |
| N1=C(SCCC1)Cc1c(O)cccc1Cl | 6.701 | A VAL 108 A THR 111 A PHE 114 A GLN 115 A VAL 118 A TRP 124 A VAL 134 A LEU 135 A ASP 138 A CYS 210 A TYR 312 A ILE 316 A TYR 320 |
| N1=C(SCCC1)Cc1c(cc(cc1C)C#N)C | 6.697 | A VAL 108 A THR 111 A GLN 115 A VAL 118 A TRP 124 A VAL 134 A LEU 135 A ASP 138 A CYS 210 A TYR 312 A ILE 316 A TYR 320 |
| N1=C(SCCC1)Nc1c(Br)cccc1 | 6.695 | A VAL 108 A ASP 138 A TYR 139 A MET 142 A VAL 230 A TRP 287 A ILE 290 A ILE 294 A ILE 316 A GLY 319 A TYR 320 |
| N1=C(SCCC1)Nc1c(Cl)cc(N)cc1 | 6.695 | A ASP 138 A TYR 139 A MET 142 A LYS 227 A VAL 230 A TRP 287 A ILE 290 A HIS 291 A ILE 294 A ILE 316 A GLY 319 A TYR 320 |
| N1=C(SCCC1)Nc1c(onc1C)CC | 6.691 | A VAL 108 A THR 111 A PHE 114 A GLN 115 A VAL 118 A TRP 124 A VAL 134 A LEU 135 A ASP 138 A CYS 210 A TYR 312 A ILE 316 A TYR 320 |
| N1=C(SCCC1)Nc1c(N(C)C)c(Cl)ccc1 | 6.684 | A LEU 135 A ASP 138 A TYR 139 A MET 142 A SER 211 A LEU 212 A LYS 227 A VAL 230 A TRP 287 A ILE 290 A HIS 291 A ILE 294 A TYR 312 A ILE 316 |
| N1=C(SCCC1)N[C@H]1CCCn2nccc12 | 6.684 | A VAL 108 A THR 111 A GLN 115 A TRP 124 A VAL 134 A LEU 135 A ASP 138 A TRP 287 A ILE 290 A TYR 312 A CYS 315 A ILE 316 A GLY 319 A TYR 320 |
| N1=C(SCCC1)Nc1c(ccnc1C)C | 6.683 | A VAL 108 A THR 111 A PHE 114 A GLN 115 A VAL 118 A TRP 124 A VAL 134 A LEU 135 A ASP 138 A CYS 210 A TYR 312 A ILE 316 A TYR 320 |
| N1=C(SCCC1)Nc1c(Br)cc(N)cc1 | 6.679 | A ASP 138 A TYR 139 A MET 142 A LYS 227 A VAL 230 A TRP 287 A ILE 290 A HIS 291 A ILE 294 A ILE 316 A GLY 319 A TYR 320 |
| N1=C(SCCC1)Nc1c(CCC)cccc1 | 6.675 | A VAL 108 A THR 111 A GLN 115 A VAL 134 A ASP 138 A MET 142 A TRP 287 A ILE 290 A ILE 294 A TYR 312 A CYS 315 A ILE 316 A GLY 319 A TYR 320 |
| N1=C(SCCC1)Nc1c(ccc2nsnc12)C | 6.673 | A LEU 135 A TYR 139 A MET 142 A SER 211 A LEU 212 A TYR 219 A LYS 227 A VAL 230 A TRP 287 A ILE 290 A HIS 291 A ILE 294 A TYR 312 A ILE 316 |
| N1=C(SCCC1)Nc1ncnc(N)c1OC | 6.672 | A VAL 108 A THR 111 A GLN 115 A VAL 118 A TRP 124 A VAL 134 A LEU 135 A ASP 138 A CYS 210 A TYR 312 A ILE 316 A TYR 320 |
| N1=C(SCCC1)Cc1c([nH+]c(N)cc1C)C | 6.669 | A ASP 138 A TYR 139 A MET 142 A MET 226 A LYS 227 A VAL 230 A PHE 231 A TRP 287 A ILE 290 A HIS 291 A ILE 294 A LEU 295 A TYR 312 A ILE 316 |
| N1=C(SCCC1)Nc1n(nc(c1N)C)CC | 6.666 | A VAL 108 A ASP 138 A TYR 139 A MET 142 A VAL 230 A TRP 287 A ILE 290 A HIS 291 A ILE 294 A TYR 312 A ILE 316 A GLY 319 A TYR 320 |
| N1=C(SCCC1)Nc1c(OC)ccc(N)c1 | 6.664 | A VAL 108 A THR 111 A VAL 134 A LEU 135 A ASP 138 A TRP 287 A ILE 290 A TYR 312 A CYS 315 A ILE 316 A GLY 319 A TYR 320 |
| N1=C(SCCC1)Nc1c(Cl)ccc(Cl)c1 | 6.661 | A VAL 108 A THR 111 A GLN 115 A TRP 124 A VAL 134 A LEU 135 A ILE 137 A ASP 138 A CYS 210 A ILE 316 A TYR 320 |
| N1=C(SCCC1)Nc1c(C(C)C)cc(O)c(c1)C | 6.66 | A VAL 108 A THR 111 A PHE 114 A GLN 115 A TRP 124 A VAL 134 A LEU 135 A ASP 138 A CYS 210 A SER 211 A TYR 312 A ILE 316 A TYR 320 |
| N1=C(SCCC1)Nc1c(C)cccc1 | 6.654 | A VAL 108 A THR 111 A GLN 115 A VAL 134 A ASP 138 A TRP 287 A ILE 290 A TYR 312 A ILE 316 A GLY 319 A TYR 320 |
| N1=C(SCCC1)N1c2c(CCC1)cc(OC)cc2OC | 6.653 | A LEU 135 A ASP 138 A TYR 139 A MET 142 A SER 211 A LEU 212 A LYS 227 A VAL 230 A TRP 287 A ILE 290 A HIS 291 A ILE 294 A TYR 312 A ILE 316 A GLY 319 |
| N1=C(SCCC1)Nc1c(cc(N)c(Cl)c1)C | 6.649 | A VAL 108 A THR 111 A GLN 115 A TRP 124 A VAL 134 A LEU 135 A ILE 137 A ASP 138 A TRP 287 A ILE 290 A TYR 312 A CYS 315 A ILE 316 A GLY 319 A TYR 320 |
| N1=C(SCCC1)Nc1c(CC)cccc1 | 6.646 | A VAL 108 A THR 111 A GLN 115 A VAL 134 A ASP 138 A MET 142 A TRP 287 A ILE 290 A TYR 312 A CYS 315 A ILE 316 A GLY 319 A TYR 320 |
| N1=C(SCCC1)N1C[C@H]2[C@H](CCC2)C1 | 6.642 | A VAL 108 A THR 111 A GLN 115 A TRP 124 A VAL 134 A ASP 138 A TRP 287 A ILE 290 A CYS 315 A ILE 316 A GLY 319 A TYR 320 |
| N1=C(SCCC1)N[C@@H]1[C@@H](F)CCCC1 | 6.641 | A VAL 108 A THR 111 A GLN 115 A VAL 134 A ASP 138 A TRP 287 A ILE 290 A TYR 312 A CYS 315 A ILE 316 A GLY 319 A TYR 320 |
| N1=C(SCCC1)Nc1c([nH]nc1C)C | 6.638 | A VAL 108 A THR 111 A ASP 138 A MET 142 A TRP 287 A ILE 290 A ILE 294 A CYS 315 A ILE 316 A GLY 319 A TYR 320 |
| N1=C(SCCC1)N[C@H]1[C@@H](CCCC1)CO | 6.637 | A VAL 108 A ASP 138 A TYR 139 A MET 142 A VAL 230 A TRP 287 A ILE 290 A HIS 291 A ILE 294 A TYR 312 A CYS 315 A ILE 316 A GLY 319 A TYR 320 |
| N1=C(SCCC1)Nc1c(OC)cccc1 | 6.633 | A VAL 108 A GLN 115 A ASP 138 A TYR 139 A MET 142 A VAL 230 A TRP 287 A ILE 290 A ILE 294 A TYR 312 A ILE 316 A GLY 319 A TYR 320 |
| N1=C(SCCC1)Nc1c(Cl)cc(OC)cc1 | 6.633 | A VAL 108 A THR 111 A PHE 114 A GLN 115 A TRP 124 A VAL 134 A ASP 138 A TRP 287 A ILE 290 A CYS 315 A ILE 316 A GLY 319 A TYR 320 |
| N1=C(SCCC1)Nc1c(C)ccnc1Cl | 6.629 | A VAL 108 A THR 111 A GLN 115 A VAL 134 A ILE 137 A ASP 138 A TRP 287 A ILE 290 A TYR 312 A CYS 315 A ILE 316 A GLY 319 A TYR 320 |
| N1=C(SCCC1)Oc1c(C)cc(F)cc1C | 6.628 | A ASP 138 A TYR 139 A MET 142 A LYS 227 A VAL 230 A TRP 287 A ILE 290 A HIS 291 A ILE 294 A GLU 297 A TYR 312 A ILE 316 |
| N1=C(SCCC1)Nc1c(CO)cccc1 | 6.624 | A VAL 108 A ASP 138 A TYR 139 A MET 142 A VAL 230 A TRP 287 A ILE 290 A ILE 294 A ILE 316 A GLY 319 A TYR 320 |
| N1=C(SCCC1)Nc1c(COC)cccc1 | 6.612 | A VAL 108 A THR 111 A GLN 115 A VAL 134 A ASP 138 A MET 142 A TRP 287 A ILE 290 A ILE 294 A TYR 312 A ILE 316 A GLY 319 A TYR 320 |
| N1=C(SCCC1)Nc1c(ccn(c1=O)C)C | 6.612 | A VAL 108 A THR 111 A PHE 114 A GLN 115 A TRP 124 A VAL 134 A ASP 138 A TRP 287 A ILE 290 A TYR 312 A ILE 316 A GLY 319 A TYR 320 |
| N1=C(SCCC1)Nc1c([nH+]ccc1)N(C)C | 6.605 | A VAL 108 A THR 111 A PHE 114 A GLN 115 A TRP 124 A VAL 134 A LEU 135 A ASP 138 A CYS 210 A TYR 312 A ILE 316 A TYR 320 |
| N1=C(SCCC1)Nc1c(C)cncc1C | 6.602 | A VAL 108 A THR 111 A GLN 115 A VAL 134 A ILE 137 A ASP 138 A TRP 287 A ILE 290 A TYR 312 A ILE 316 A GLY 319 A TYR 320 |
| N1=C(SCCC1)Nc1c(OC)c(OC)ccc1 | 6.601 | A VAL 108 A THR 111 A PHE 114 A GLN 115 A TRP 124 A VAL 134 A ILE 137 A ASP 138 A TRP 287 A ILE 290 A TYR 312 A ILE 316 A GLY 319 A TYR 320 |
| N1=C(SCCC1)Nc1c2CCCCc2sc1 | 6.592 | A VAL 108 A THR 111 A PHE 114 A GLN 115 A TRP 124 A VAL 134 A LEU 135 A ASP 138 A TRP 287 A ILE 290 A TYR 312 A ILE 316 A GLY 319 A TYR 320 |
| N1=C(SCCC1)Nc1c(N(C)C)ccc(Cl)c1 | 6.591 | A VAL 108 A THR 111 A PHE 114 A GLN 115 A TRP 124 A VAL 134 A LEU 135 A ASP 138 A CYS 210 A TYR 312 A ILE 316 A TYR 320 |
| N1=C(SCCC1)Nc1c(N)c(OC)ccc1 | 6.587 | A VAL 108 A ASP 138 A TYR 139 A MET 142 A LYS 227 A VAL 230 A TRP 287 A ILE 290 A HIS 291 A ILE 294 A ILE 316 A GLY 319 A TYR 320 |
| N1=C(SCCC1)Cc1ncccc1C | 6.586 | A VAL 108 A THR 111 A GLN 115 A VAL 134 A LEU 135 A ILE 137 A ASP 138 A TRP 287 A ILE 290 A ILE 316 A GLY 319 A TYR 320 |
| N1=C(SCCC1)Nc1c(OC)cc(N)cc1 | 6.585 | A GLN 115 A ASP 138 A TYR 139 A MET 142 A LYS 227 A VAL 230 A TRP 287 A ILE 290 A HIS 291 A ILE 294 A TYR 312 A ILE 316 A GLY 319 A TYR 320 |
| N1=C(SCCC1)Cc1c(cccc1F)C | 6.584 | A VAL 108 A THR 111 A GLN 115 A VAL 134 A LEU 135 A ILE 137 A ASP 138 A TRP 287 A ILE 290 A TYR 312 A ILE 316 A GLY 319 A TYR 320 |
| N1=C(SCCC1)N1c2c(CCC1)cccc2OC | 6.581 | A LEU 135 A ASP 138 A TYR 139 A MET 142 A SER 211 A LEU 212 A LYS 227 A VAL 230 A TRP 287 A ILE 290 A HIS 291 A ILE 294 A TYR 312 A ILE 316 |
| N1=C(SCCC1)Nc1c(Cl)cccc1 | 6.573 | A VAL 108 A THR 111 A GLN 115 A VAL 134 A ASP 138 A TRP 287 A ILE 290 A TYR 312 A ILE 316 A GLY 319 A TYR 320 |
| N1=C(SCCC1)Nc1c(O)cccc1 | 6.573 | A VAL 108 A THR 111 A GLN 115 A TRP 124 A VAL 134 A LEU 135 A ILE 137 A ASP 138 A ASN 141 A TRP 287 A ILE 316 A GLY 319 A TYR 320 |
| N1=C(SCCC1)Nc1c(Cl)cncc1Cl | 6.57 | A VAL 108 A THR 111 A GLN 115 A VAL 134 A ILE 137 A ASP 138 A TRP 287 A ILE 290 A ILE 316 A GLY 319 A TYR 320 |
| N1=C(SCCC1)Nc1ccc(Br)cc1 | 6.569 | A VAL 108 A THR 111 A GLN 115 A TRP 124 A VAL 134 A LEU 135 A ASP 138 A TRP 287 A ILE 290 A ILE 316 A GLY 319 A TYR 320 |
| N1=C(SCCC1)N1CCSc2c1cccc2 | 6.568 | A VAL 108 A THR 111 A PHE 114 A GLN 115 A VAL 134 A ASP 138 A TRP 287 A ILE 290 A TYR 312 A ILE 316 A GLY 319 A TYR 320 |
| N1=C(SCCC1)N1CCSc2nc(cc(c12)C)C | 6.567 | A VAL 108 A THR 111 A PHE 114 A GLN 115 A VAL 118 A TRP 124 A VAL 134 A LEU 135 A ASP 138 A CYS 210 A SER 211 A TYR 312 A ILE 316 A TYR 320 |
| N1=C(SCCC1)Nc1ccc(OC)cc1 | 6.566 | A VAL 108 A THR 111 A GLN 115 A TRP 124 A VAL 134 A LEU 135 A ASP 138 A TRP 287 A ILE 290 A TYR 312 A CYS 315 A ILE 316 A GLY 319 A TYR 320 |
| N1=C(SCCC1)Nc1cc(N)ccc1Br | 6.564 | A VAL 108 A ASP 138 A TYR 139 A MET 142 A TRP 287 A ILE 290 A ILE 294 A TYR 312 A ILE 316 A GLY 319 A TYR 320 |
| N1=C(SCCC1)NC[C@@H]1N(CCC1)CC | 6.546 | A VAL 108 A THR 111 A PHE 114 A GLN 115 A TRP 124 A VAL 134 A LEU 135 A ASP 138 A ASN 141 A TRP 287 A ILE 290 A ILE 316 A GLY 319 A TYR 320 |
| N1=C(SCCC1)N[C@H]1[C@@H](CCCC1)CC | 6.541 | A VAL 108 A ASP 138 A TYR 139 A ASN 141 A MET 142 A VAL 230 A TRP 287 A ILE 290 A HIS 291 A ILE 294 A TYR 312 A CYS 315 A ILE 316 A GLY 319 A TYR 320 |
| N1=C(SCCC1)Nc1c(OC)cccc1OC | 6.54 | A VAL 108 A THR 111 A GLN 115 A TRP 124 A VAL 134 A ILE 137 A ASP 138 A ASN 141 A TRP 287 A ILE 290 A TYR 312 A ILE 316 A GLY 319 A TYR 320 |
| N1=C(SCCC1)N[C@@H]1[C@H](CCCC1)C | 6.535 | A VAL 108 A THR 111 A GLN 115 A VAL 134 A LEU 135 A ASP 138 A TRP 287 A ILE 290 A TYR 312 A CYS 315 A ILE 316 A GLY 319 A TYR 320 |
| N1=C(SCCC1)N[C@@H](C1CCC1)C(F)(F)F | 6.532 | A VAL 108 A THR 111 A PHE 114 A GLN 115 A TRP 124 A VAL 134 A LEU 135 A ASP 138 A TRP 287 A ILE 316 A TYR 320 |
| N1=C(SCCC1)Nc1c(nccc1)C | 6.528 | A VAL 108 A THR 111 A GLN 115 A VAL 134 A ASP 138 A TRP 287 A ILE 290 A TYR 312 A ILE 316 A GLY 319 A TYR 320 |
| N1=C(SCCC1)Nc1c(N)cncc1 | 6.523 | A VAL 108 A THR 111 A GLN 115 A VAL 134 A LEU 135 A ILE 137 A ASP 138 A ASN 141 A TRP 287 A ILE 316 A GLY 319 A TYR 320 |
| N1=C(SCCC1)NCc1c[nH]nc1C | 6.519 | A VAL 108 A THR 111 A PHE 114 A GLN 115 A TRP 124 A VAL 134 A LEU 135 A ILE 137 A ASP 138 A MET 142 A TRP 287 A ILE 290 A CYS 315 A ILE 316 A GLY 319 A TYR 320 |
| N1=C(SCCC1)Oc1c(CN)cc(Cl)cc1 | 6.518 | A VAL 108 A THR 111 A MET 112 A PHE 114 A GLN 115 A VAL 118 A TRP 124 A VAL 134 A LEU 135 A ASP 138 A CYS 210 A TYR 312 A ILE 316 A TYR 320 |
| N1=C(SCCC1)Nc1c(SC(C)C)cccc1 | 6.514 | A VAL 108 A THR 111 A GLN 115 A VAL 134 A ASP 138 A MET 142 A TRP 287 A ILE 290 A ILE 294 A TYR 312 A CYS 315 A ILE 316 A GLY 319 A TYR 320 |
| N1=C(SCCC1)Oc1c(C)cc(Cl)cc1C | 6.51 | A VAL 108 A THR 111 A PHE 114 A GLN 115 A VAL 118 A TRP 124 A VAL 134 A LEU 135 A ASP 138 A CYS 210 A ILE 316 A TYR 320 |
| N1=C(SCCC1)Nc1c(N)ccnc1 | 6.507 | A VAL 108 A THR 111 A GLN 115 A TRP 124 A VAL 134 A ILE 137 A ASP 138 A ASN 141 A TRP 287 A ILE 316 A GLY 319 A TYR 320 |
| N1=C(SCCC1)Cc1c(cccc1C)C | 6.507 | A VAL 108 A THR 111 A GLN 115 A VAL 134 A LEU 135 A ASP 138 A TRP 287 A ILE 290 A TYR 312 A ILE 316 A GLY 319 A TYR 320 |
| N1=C(SCCC1)N(c1c(Cl)cc(Cl)cc1)C | 6.496 | A VAL 108 A THR 111 A PHE 114 A GLN 115 A VAL 118 A TRP 124 A VAL 134 A LEU 135 A ILE 137 A ASP 138 A CYS 210 A TYR 312 A ILE 316 A TYR 320 |
| N1=C(SCCC1)Sc1c(cccc1C)C | 6.491 | A VAL 108 A THR 111 A GLN 115 A VAL 134 A LEU 135 A ILE 137 A ASP 138 A TRP 287 A ILE 290 A TYR 312 A ILE 316 A GLY 319 A TYR 320 |
| N1=C(SCCC1)Cc1[n+](CC)cccc1 | 6.487 | A VAL 108 A THR 111 A GLN 115 A VAL 134 A LEU 135 A ILE 137 A ASP 138 A TRP 287 A ILE 290 A ILE 316 A GLY 319 A TYR 320 |
| N1=C(SCCC1)Cc1c(F)cccc1F | 6.487 | A VAL 108 A THR 111 A GLN 115 A VAL 134 A LEU 135 A ILE 137 A ASP 138 A TRP 287 A ILE 290 A TYR 312 A ILE 316 A GLY 319 A TYR 320 |
| N1=C(SCCC1)N[C@@H]1[C@H](CCCC1)CN | 6.467 | A VAL 108 A THR 111 A GLN 115 A VAL 134 A LEU 135 A ASP 138 A MET 142 A TRP 287 A ILE 290 A TYR 312 A CYS 315 A ILE 316 A GLY 319 A TYR 320 |
| N1=C(SCCC1)N[C@H]1[C@@H](C[C@@H](CC1)C)C | 6.465 | A VAL 108 A THR 111 A GLN 115 A VAL 118 A TRP 124 A VAL 134 A LEU 135 A ASP 138 A CYS 210 A SER 211 A TRP 287 A TYR 312 A ILE 316 A TYR 320 |
| N1=C(SCCC1)Nc1c(ccnc1)C | 6.462 | A VAL 108 A THR 111 A GLN 115 A VAL 134 A ILE 137 A ASP 138 A TRP 287 A ILE 290 A TYR 312 A ILE 316 A GLY 319 A TYR 320 |
| N1=C(SCCC1)Nc1c(OC)ccc(Cl)c1 | 6.458 | A VAL 108 A THR 111 A GLN 115 A TRP 124 A VAL 134 A ILE 137 A ASP 138 A TRP 287 A ILE 290 A TYR 312 A ILE 316 A GLY 319 A TYR 320 |
| N1=C(SCCC1)Nc1c([nH]nc1)CC | 6.458 | A VAL 108 A THR 111 A PHE 114 A GLN 115 A VAL 118 A TRP 124 A VAL 134 A LEU 135 A ASP 138 A CYS 210 A TYR 312 A ILE 316 A TYR 320 |
| N1=C(SCCC1)N[C@@H]([C@@H]1CCCOC1)C | 6.454 | A VAL 108 A THR 111 A PHE 114 A GLN 115 A TRP 124 A VAL 134 A LEU 135 A ASP 138 A TRP 287 A ILE 290 A ILE 316 A GLY 319 A TYR 320 |
| N1=C(SCCC1)N[C@@H](C(C)C)C#C | 6.451 | A VAL 108 A ASP 138 A TYR 139 A ASN 141 A MET 142 A TRP 287 A ILE 290 A ILE 294 A TYR 312 A CYS 315 A ILE 316 A GLY 319 A TYR 320 |
| N1=C(SCCC1)Nc1c(O)ccc(Br)c1 | 6.447 | A VAL 108 A THR 111 A GLN 115 A TRP 124 A VAL 134 A ASP 138 A TRP 287 A ILE 290 A TYR 312 A ILE 316 A GLY 319 A TYR 320 |
| N1=C(SCCC1)NCc1c(occ1)C | 6.443 | A VAL 108 A THR 111 A PHE 114 A GLN 115 A TRP 124 A VAL 134 A LEU 135 A ASP 138 A ASN 141 A TRP 287 A ILE 290 A ILE 316 A GLY 319 A TYR 320 |
| N1=C(SCCC1)N[C@@H](C1CC1)C(F)(F)F | 6.443 | A VAL 108 A THR 111 A GLN 115 A VAL 134 A ASP 138 A TRP 287 A ILE 290 A TYR 312 A ILE 316 A GLY 319 A TYR 320 |
| N1=C(SCCC1)Cc1c(cccc1Cl)C | 6.432 | A VAL 108 A THR 111 A GLN 115 A VAL 134 A LEU 135 A ASP 138 A TRP 287 A ILE 290 A TYR 312 A ILE 316 A GLY 319 A TYR 320 |
| N1=C(SCCC1)N(CC)c1ccc(cc1)C | 6.43 | A VAL 108 A THR 111 A PHE 114 A GLN 115 A TRP 124 A VAL 134 A LEU 135 A ASP 138 A CYS 210 A TRP 287 A TYR 312 A ILE 316 A GLY 319 A TYR 320 |
| N1=C(SCCC1)Nc1c(n(nc1C)CC)C | 6.429 | A THR 111 A GLN 115 A TRP 124 A VAL 134 A LEU 135 A ASP 138 A TRP 287 A ILE 290 A TYR 312 A ILE 316 A GLY 319 A TYR 320 |
| N1=C(SCCC1)N[C@@H](C1CCCC1)CN | 6.425 | A VAL 108 A THR 111 A PHE 114 A GLN 115 A TRP 124 A VAL 134 A LEU 135 A ILE 137 A ASP 138 A TRP 287 A ILE 290 A CYS 315 A ILE 316 A GLY 319 A TYR 320 |
| N1=C(SCCC1)CN1[C@H](CCCCC1)C | 6.422 | A VAL 108 A THR 111 A GLN 115 A VAL 134 A LEU 135 A ASP 138 A ASN 141 A TRP 287 A ILE 290 A ILE 316 A GLY 319 A TYR 320 |
| N1=C(SCCC1)N(C1CC1)C1=CCCCC1 | 6.419 | A LEU 135 A ASP 138 A TYR 139 A MET 142 A SER 211 A LEU 212 A ASP 223 A LYS 227 A VAL 230 A ILE 290 A HIS 291 A ILE 294 A TYR 312 A ILE 316 |
| N1=C(SCCC1)Nc1c(nn(c1C)C)C | 6.401 | A VAL 108 A THR 111 A GLN 115 A VAL 118 A TRP 124 A VAL 134 A LEU 135 A ILE 137 A ASP 138 A CYS 210 A TYR 312 A ILE 316 A TYR 320 |
| N1=C(SCCC1)N[C@@H]1[C@H](OCC1)C=C | 6.397 | A VAL 108 A ASP 138 A ASN 141 A MET 142 A TRP 287 A ILE 290 A ILE 294 A CYS 315 A ILE 316 A GLY 319 A TYR 320 |
| N1=C(SCCC1)N[C@H]1[C@@H](CCCC1)C(C)(C)C | 6.382 | A LEU 135 A ASP 138 A TYR 139 A MET 142 A SER 211 A LEU 212 A MET 226 A LYS 227 A VAL 230 A TRP 287 A ILE 290 A HIS 291 A ILE 294 A TYR 312 A ILE 316 |
| N1=C(SCCC1)N[C@H](c1c(F)cncc1F)C | 6.38 | A VAL 108 A THR 111 A GLN 115 A ILE 137 A ASP 138 A MET 142 A TRP 287 A ILE 290 A ILE 294 A TYR 312 A ILE 316 A GLY 319 A TYR 320 |
| N1=C(SCCC1)Nc1c(SCC)cccc1 | 6.374 | A VAL 108 A THR 111 A PHE 114 A GLN 115 A VAL 118 A TRP 124 A VAL 134 A LEU 135 A ASP 138 A CYS 210 A SER 211 A TYR 312 A ILE 316 A TYR 320 |
| N1=C(SCCC1)Nc1c(O)ccnc1 | 6.367 | A VAL 108 A THR 111 A PHE 114 A GLN 115 A TRP 124 A VAL 134 A ILE 137 A ASP 138 A ASN 141 A TRP 287 A ILE 316 A GLY 319 A TYR 320 |
| N1=C(SCCC1)Nc1c(CC)ccnc1 | 6.365 | A VAL 108 A THR 111 A GLN 115 A ASP 138 A TYR 139 A MET 142 A VAL 230 A TRP 287 A ILE 290 A ILE 294 A TYR 312 A ILE 316 A GLY 319 A TYR 320 |
| N1=C(SCCC1)Nc1c(cncc1)C | 6.361 | A VAL 108 A THR 111 A GLN 115 A VAL 134 A ASP 138 A TRP 287 A ILE 290 A TYR 312 A ILE 316 A GLY 319 A TYR 320 |
| N1=C(SCCC1)N(c1c(CN)cccc1)C | 6.356 | A VAL 108 A THR 111 A MET 112 A PHE 114 A GLN 115 A VAL 118 A TRP 124 A VAL 134 A LEU 135 A ILE 137 A ASP 138 A CYS 210 A TYR 312 A ILE 316 A TYR 320 |
| N1=C(SCCC1)Nc1c(nsc1)c1ccccc1 | 6.356 | A THR 111 A PHE 114 A GLN 115 A VAL 118 A TRP 124 A VAL 134 A LEU 135 A ASP 138 A CYS 210 A ILE 290 A TYR 312 A ILE 316 A TYR 320 |
| N1=C(SCCC1)N[C@@H]1CCC=CC1 | 6.353 | A VAL 108 A THR 111 A GLN 115 A VAL 134 A LEU 135 A ASP 138 A ASN 141 A TRP 287 A ILE 316 A GLY 319 A TYR 320 |
| N1=C(SCCC1)N(c1c(F)cccc1)C | 6.347 | A VAL 108 A THR 111 A GLN 115 A VAL 134 A ASP 138 A TRP 287 A ILE 290 A TYR 312 A CYS 315 A ILE 316 A GLY 319 A TYR 320 |
| N1=C(SCCC1)N[C@@H]1[C@@H](N)CCCC1 | 6.344 | A VAL 108 A THR 111 A GLN 115 A VAL 134 A ILE 137 A ASP 138 A TRP 287 A ILE 290 A TYR 312 A ILE 316 A GLY 319 A TYR 320 |
| N1=C(SCCC1)[C@H]1CNCc2c1cccc2 | 6.335 | A ASP 138 A TYR 139 A MET 142 A SER 211 A LEU 212 A TYR 219 A ASP 223 A LYS 227 A VAL 230 A TRP 287 A ILE 290 A HIS 291 A ILE 294 A TYR 312 A ILE 316 |
| N1=C(SCCC1)N(c1cc(ccc1)C)CC | 6.329 | A THR 111 A PHE 114 A GLN 115 A VAL 118 A TYR 119 A TRP 124 A VAL 134 A LEU 135 A ASP 138 A ILE 208 A GLU 209 A CYS 210 A LEU 309 A TYR 312 A TYR 313 |
| N1=C(SCCC1)N(CC)c1cc(Cl)ccc1 | 6.328 | A ASP 138 A TYR 139 A MET 142 A LYS 227 A VAL 230 A TRP 287 A ILE 290 A HIS 291 A ILE 294 A TYR 312 A ILE 316 |
| N1=C(SCCC1)N[C@@H]1C[C@@H](O[C@H]1C)C | 6.327 | A VAL 108 A ASP 138 A ASN 141 A MET 142 A VAL 230 A TRP 287 A ILE 290 A HIS 291 A ILE 294 A TYR 312 A CYS 315 A ILE 316 A GLY 319 A TYR 320 |
| N1=C(SCCC1)NC[C@@H]1N(CCC1)CCC | 6.327 | A VAL 108 A THR 111 A PHE 114 A GLN 115 A VAL 134 A LEU 135 A ASP 138 A ASN 141 A MET 142 A TRP 287 A ILE 290 A ILE 294 A CYS 315 A ILE 316 A GLY 319 A TYR 320 |
| N1=C(SCCC1)N(c1cc(ccc1)C)C | 6.312 | A VAL 108 A THR 111 A GLN 115 A VAL 134 A LEU 135 A ASP 138 A TRP 287 A ILE 290 A TYR 312 A CYS 315 A ILE 316 A GLY 319 A TYR 320 |
| N1=C(SCCC1)NCC1(OCCO1)C | 6.311 | A VAL 108 A THR 111 A PHE 114 A GLN 115 A VAL 134 A ASP 138 A TRP 287 A ILE 290 A ILE 316 A GLY 319 A TYR 320 |
| N1=C(SCCC1)N[C@H]1[C@H](NC(=O)CC1)C | 6.311 | A VAL 108 A ASP 138 A TYR 139 A MET 142 A TRP 287 A ILE 290 A ILE 294 A TYR 312 A ILE 316 A GLY 319 A TYR 320 |
| N1=C(SCCC1)Nc1c(S)cccc1 | 6.294 | A VAL 108 A THR 111 A GLN 115 A VAL 134 A ILE 137 A ASP 138 A TRP 287 A ILE 290 A TYR 312 A ILE 316 A GLY 319 A TYR 320 |
| N1=C(SCCC1)Nc1c(OCC)nccc1 | 6.291 | A VAL 108 A THR 111 A PHE 114 A GLN 115 A VAL 134 A ASP 138 A TRP 287 A ILE 290 A TYR 312 A CYS 315 A ILE 316 A GLY 319 A TYR 320 |
| N1=C(SCCC1)N[C@@H](C1CCC1)C1CC1 | 6.29 | A VAL 108 A THR 111 A GLN 115 A VAL 134 A ASP 138 A ASN 141 A MET 142 A TRP 287 A ILE 290 A ILE 294 A TYR 312 A ILE 316 A GLY 319 A TYR 320 |
| N1=C(SCCC1)NCC1(CC1)C | 6.283 | A VAL 108 A THR 111 A PHE 114 A GLN 115 A VAL 134 A LEU 135 A ILE 137 A ASP 138 A TRP 287 A ILE 290 A ILE 316 A GLY 319 A TYR 320 |
| N1=C(SCCC1)NC1(OCCO1)C(F)F | 6.277 | A VAL 108 A THR 111 A PHE 114 A GLN 115 A TRP 124 A VAL 134 A LEU 135 A ASP 138 A ILE 316 A TYR 320 |
| N1=C(SCCC1)NC1CCCCC1 | 6.275 | A VAL 108 A ASP 138 A TYR 139 A MET 142 A VAL 230 A TRP 287 A ILE 290 A HIS 291 A ILE 294 A TYR 312 A CYS 315 A ILE 316 A GLY 319 A TYR 320 |
| N1=C(SCCC1)N[C@@H]1[C@H](CCC[C@H]1C)C | 6.271 | A VAL 108 A THR 111 A GLN 115 A VAL 134 A ILE 137 A ASP 138 A TRP 287 A ILE 290 A TYR 312 A CYS 315 A ILE 316 A GLY 319 A TYR 320 |
| N1=C(SCCC1)Nc1c(nn(c1)C)CC | 6.27 | A VAL 108 A ASP 138 A TYR 139 A MET 142 A VAL 230 A TRP 287 A ILE 290 A HIS 291 A ILE 294 A ILE 316 A GLY 319 A TYR 320 |
| N1=C(SCCC1)N(c1c(N)cccc1)C | 6.269 | A VAL 108 A THR 111 A PHE 114 A GLN 115 A VAL 118 A TRP 124 A VAL 134 A LEU 135 A ILE 137 A ASP 138 A CYS 210 A TYR 312 A ILE 316 A TYR 320 |
| N1=C(SCCC1)Oc1c(cccc1C)C | 6.266 | A ASP 138 A TYR 139 A MET 142 A LYS 227 A VAL 230 A TRP 287 A ILE 290 A HIS 291 A ILE 294 A GLU 297 A TYR 312 A ILE 316 |
| N1=C(SCCC1)N[C@H]1C(OCC1)(C)C | 6.265 | A VAL 108 A ASP 138 A TYR 139 A ASN 141 A MET 142 A TRP 287 A ILE 290 A ILE 294 A TYR 312 A CYS 315 A ILE 316 A GLY 319 A TYR 320 |
| N1=C(SCCC1)Nc1c(OC)c(Cl)ccc1 | 6.241 | A VAL 108 A THR 111 A PHE 114 A GLN 115 A TRP 124 A VAL 134 A ILE 137 A ASP 138 A TRP 287 A ILE 290 A TYR 312 A ILE 316 A GLY 319 A TYR 320 |
| N1=C(SCCC1)N[C@H]1[C@@H](CCNC1)C | 6.241 | A VAL 108 A ASP 138 A TYR 139 A ASN 141 A MET 142 A TRP 287 A ILE 290 A ILE 294 A TYR 312 A CYS 315 A ILE 316 A GLY 319 A TYR 320 |
| N1=C(SCCC1)Nc1c(N(C)C)cccc1 | 6.239 | A VAL 108 A THR 111 A PHE 114 A GLN 115 A TRP 124 A VAL 134 A LEU 135 A ASP 138 A CYS 210 A TYR 312 A ILE 316 A TYR 320 |
| N1=C(SCCC1)N[C@@H]1[C@@H](OC)CCCC1 | 6.239 | A VAL 108 A THR 111 A PHE 114 A GLN 115 A TRP 124 A VAL 134 A LEU 135 A ASP 138 A MET 142 A TRP 287 A ILE 290 A TYR 312 A CYS 315 A ILE 316 A GLY 319 A TYR 320 |
| N1=C(SCCC1)NCc1cc[nH]c1 | 6.237 | A VAL 108 A THR 111 A PHE 114 A GLN 115 A TRP 124 A VAL 134 A LEU 135 A ASP 138 A TRP 287 A ILE 290 A CYS 315 A ILE 316 A GLY 319 A TYR 320 |
| N1=C(SCCC1)Nc1cccnc1Cl | 6.236 | A VAL 108 A THR 111 A GLN 115 A VAL 118 A TRP 124 A VAL 134 A LEU 135 A ILE 137 A ASP 138 A CYS 210 A TYR 312 A ILE 316 A TYR 320 |
| N1=C(SCCC1)NCc1nc[nH]c1C | 6.23 | A VAL 108 A THR 111 A GLN 115 A VAL 134 A LEU 135 A ILE 137 A ASP 138 A TRP 287 A ILE 290 A ILE 316 A GLY 319 A TYR 320 |
| N1=C(SCCC1)N[C@H](CC)c1scc(n1)C | 6.23 | A VAL 108 A ASP 138 A TYR 139 A ASN 141 A MET 142 A LYS 227 A VAL 230 A TRP 287 A ILE 290 A HIS 291 A ILE 294 A TYR 312 A ILE 316 A GLY 319 A TYR 320 |
| N1=C(SCCC1)Nc1cnn(c1Cl)C | 6.225 | A VAL 108 A THR 111 A GLN 115 A VAL 134 A ASP 138 A TRP 287 A ILE 290 A TYR 312 A CYS 315 A ILE 316 A GLY 319 A TYR 320 |
| N1=C(SCCC1)NCc1scc(c1)C | 6.223 | A VAL 108 A THR 111 A GLN 115 A TRP 124 A VAL 134 A LEU 135 A ASP 138 A TRP 287 A ILE 290 A CYS 315 A ILE 316 A GLY 319 A TYR 320 |
| N1=C(SCCC1)N[C@H]1[C@@H](COCC1)C | 6.217 | A VAL 108 A ASP 138 A MET 142 A VAL 230 A TRP 287 A ILE 290 A ILE 294 A TYR 312 A CYS 315 A ILE 316 A GLY 319 A TYR 320 |
| N1=C(SCCC1)N1C[C@@H]2N(CC1)CCC2 | 6.217 | A VAL 108 A THR 111 A PHE 114 A GLN 115 A TRP 124 A VAL 134 A ASP 138 A TRP 287 A ILE 290 A TYR 312 A ILE 316 A GLY 319 A TYR 320 |
| N1=C(SCCC1)N(c1c(cccc1C)C)C | 6.212 | A ASP 138 A TYR 139 A MET 142 A LYS 227 A VAL 230 A TRP 287 A ILE 290 A HIS 291 A ILE 294 A GLU 297 A TYR 312 A ILE 316 |
| N1=C(SCCC1)Oc1c(cccc1F)C | 6.203 | A VAL 108 A ASP 138 A MET 142 A TRP 287 A ILE 290 A ILE 294 A TYR 312 A CYS 315 A ILE 316 A GLY 319 A TYR 320 |
| N1=C(SCCC1)C[n+]1c(cc(cc1C)C)C | 6.188 | A VAL 108 A THR 111 A PHE 114 A GLN 115 A VAL 118 A TRP 124 A VAL 134 A LEU 135 A ASP 138 A CYS 210 A TYR 312 A ILE 316 A TYR 320 |
| N1=C(SCCC1)N(c1cc(C)ccc1C)C | 6.187 | A THR 111 A PHE 114 A GLN 115 A VAL 118 A TRP 124 A VAL 134 A LEU 135 A ASP 138 A CYS 210 A SER 211 A LEU 309 A TYR 312 A TYR 313 |
| N1=C(SCCC1)N[C@@H](c1sccc1C)C | 6.183 | A VAL 108 A THR 111 A PHE 114 A GLN 115 A TRP 124 A VAL 134 A LEU 135 A ASP 138 A CYS 210 A TRP 287 A ILE 316 A TYR 320 |
| N1=C(SCCC1)Oc1c(CN)cccc1C | 6.182 | A ASP 138 A TYR 139 A MET 142 A MET 226 A LYS 227 A VAL 230 A TRP 287 A ILE 290 A HIS 291 A ILE 294 A GLU 297 A TYR 312 A ILE 316 |
| N1=C(SCCC1)Oc1c(cccc1Cl)C | 6.174 | A ASP 138 A TYR 139 A MET 142 A LYS 227 A VAL 230 A TRP 287 A ILE 290 A HIS 291 A ILE 294 A GLU 297 A TYR 312 |
| N1=C(SCCC1)NC[C@H]1N(CCC1)C | 6.171 | A VAL 108 A THR 111 A PHE 114 A GLN 115 A TRP 124 A VAL 134 A LEU 135 A ASP 138 A ASN 141 A TRP 287 A ILE 290 A ILE 316 A GLY 319 A TYR 320 |
| N1=C(SCCC1)[C@H]1CCCc2sccc12 | 6.167 | A VAL 108 A ASP 138 A MET 142 A TRP 287 A ILE 290 A ILE 294 A TYR 312 A CYS 315 A ILE 316 A GLY 319 A TYR 320 |
| N1=C(SCCC1)N[C@H]1C(CN(C1)C)(C)C | 6.141 | A VAL 108 A ASP 138 A TYR 139 A MET 142 A VAL 230 A TRP 287 A ILE 290 A ILE 294 A TYR 312 A CYS 315 A ILE 316 A GLY 319 A TYR 320 |
| N1=C(SCCC1)Nc1c(n(nc1)C)C | 6.135 | A VAL 108 A THR 111 A GLN 115 A VAL 134 A ASP 138 A TRP 287 A ILE 290 A TYR 312 A CYS 315 A ILE 316 A GLY 319 A TYR 320 |
| N1=C(SCCC1)Nc1c(SC)cccc1 | 6.118 | A VAL 108 A THR 111 A GLN 115 A VAL 134 A LEU 135 A ILE 137 A ASP 138 A TRP 287 A ILE 290 A TYR 312 A ILE 316 A GLY 319 A TYR 320 |
| N1=C(SCCC1)N[C@@H](C(C)C)C(=O)NC | 6.091 | A VAL 108 A THR 111 A ASP 138 A TYR 139 A MET 142 A VAL 230 A TRP 287 A ILE 290 A ILE 294 A TYR 312 A CYS 315 A ILE 316 A GLY 319 A TYR 320 |
| N1=C(SCCC1)N[C@@H](CC)c1sccn1 | 6.084 | A VAL 108 A THR 111 A PHE 114 A GLN 115 A VAL 118 A TRP 124 A VAL 134 A LEU 135 A ASP 138 A CYS 210 A TYR 312 A ILE 316 A TYR 320 |
| N1=C(SCCC1)Nc1c(CC)c(Cl)ccc1CC | 6.074 | A ASP 138 A TYR 139 A MET 142 A SER 211 A LEU 212 A TYR 219 A ASP 223 A LYS 227 A VAL 230 A TRP 287 A ILE 290 A HIS 291 A ILE 294 A GLU 297 A TYR 312 A ILE 316 |
| N1=C(SCCC1)Cc1[n+](cccc1)C | 6.07 | A VAL 108 A THR 111 A GLN 115 A VAL 134 A LEU 135 A ILE 137 A ASP 138 A TRP 287 A ILE 290 A TYR 312 A ILE 316 A GLY 319 A TYR 320 |
| N1=C(SCCC1)N[C@H](C1CC1)c1sccc1 | 6.066 | A VAL 108 A THR 111 A PHE 114 A GLN 115 A TRP 124 A VAL 134 A LEU 135 A ASP 138 A CYS 210 A SER 211 A ILE 316 A TYR 320 |
| N1=C(SCCC1)N[C@H](C(O)(C)C)CC | 6.062 | A VAL 108 A THR 111 A ASP 138 A TYR 139 A ASN 141 A MET 142 A TRP 287 A ILE 290 A ILE 294 A TYR 312 A CYS 315 A ILE 316 A GLY 319 A TYR 320 |
| N1=C(SCCC1)N[C@@H]1C(CCSC1)(C)C | 6.062 | A VAL 108 A ASP 138 A TYR 139 A ASN 141 A MET 142 A TRP 287 A ILE 290 A HIS 291 A ILE 294 A TYR 312 A CYS 315 A ILE 316 A GLY 319 A TYR 320 |
| N1=C(SCCC1)CN1[C@@H](CN(CC1)C)C | 6.059 | A VAL 108 A THR 111 A PHE 114 A GLN 115 A VAL 118 A TRP 124 A VAL 134 A LEU 135 A ASP 138 A CYS 210 A SER 211 A ILE 316 A TYR 320 |
| N1=C(SCCC1)N(c1c(C)cccc1)C | 6.054 | A THR 111 A PHE 114 A GLN 115 A VAL 118 A TRP 124 A VAL 134 A LEU 135 A ASP 138 A CYS 210 A TYR 312 A ILE 316 A TYR 320 |
| N1=C(SCCC1)C[n+]1c(cccc1)C | 6.053 | A VAL 108 A THR 111 A GLN 115 A VAL 134 A LEU 135 A ILE 137 A ASP 138 A TRP 287 A ILE 290 A ILE 316 A GLY 319 A TYR 320 |
| N1=C(SCCC1)N1C[C@H](F)[C@@H](F)C1 | 6.047 | A VAL 108 A THR 111 A PHE 114 A GLN 115 A TRP 124 A VAL 134 A LEU 135 A ILE 137 A ASP 138 A TRP 287 A ILE 316 A GLY 319 A TYR 320 |
| N1=C(SCCC1)NCc1c(Cl)csc1 | 6.045 | A VAL 108 A THR 111 A PHE 114 A GLN 115 A TRP 124 A VAL 134 A LEU 135 A ASP 138 A TRP 287 A ILE 290 A ILE 316 A GLY 319 A TYR 320 |
| N1=C(SCCC1)N(c1c(C)cccc1)CC | 6.045 | A THR 111 A PHE 114 A GLN 115 A VAL 118 A TYR 119 A TRP 124 A VAL 134 A LEU 135 A ILE 208 A GLU 209 A CYS 210 A SER 211 A LEU 309 A TYR 312 A TYR 313 |
| N1=C(SCCC1)N(CC)c1c(F)cccc1 | 6.044 | A THR 111 A PHE 114 A GLN 115 A VAL 118 A TRP 124 A VAL 134 A LEU 135 A ASP 138 A CYS 210 A SER 211 A TYR 312 A ILE 316 A TYR 320 |
| N1=C(SCCC1)N[C@H]1[C@@H](CCN[C@@H]1C)C | 6.043 | A VAL 108 A THR 111 A GLN 115 A VAL 118 A TRP 124 A VAL 134 A LEU 135 A ILE 137 A ASP 138 A CYS 210 A TYR 312 A ILE 316 A TYR 320 |
| N1=C(SCCC1)N[C@H](C)c1nccnc1 | 6.04 | A VAL 108 A THR 111 A GLN 115 A VAL 134 A ILE 137 A ASP 138 A TRP 287 A ILE 290 A TYR 312 A ILE 316 A GLY 319 A TYR 320 |
| N1=C(SCCC1)N(c1ccccc1)C | 6.039 | A VAL 108 A THR 111 A GLN 115 A VAL 134 A LEU 135 A ASP 138 A TRP 287 A ILE 290 A TYR 312 A CYS 315 A ILE 316 A GLY 319 A TYR 320 |
| N1=C(SCCC1)N1[C@@H]([C@H](S(=O)(=O)CC1)C)C | 6.039 | A THR 111 A PHE 114 A GLN 115 A VAL 118 A TRP 124 A VAL 134 A LEU 135 A ASP 138 A CYS 210 A TYR 312 A ILE 316 A TYR 320 |
| N1=C(SCCC1)Nn1c(C)ccc1C | 6.032 | A VAL 108 A THR 111 A GLN 115 A ASP 138 A ASN 141 A TRP 287 A ILE 290 A TYR 312 A CYS 315 A ILE 316 A GLY 319 A TYR 320 |
| N1=C(SCCC1)Oc1c(CNC)cccc1 | 6.031 | A VAL 108 A GLN 115 A ASP 138 A TYR 139 A TRP 287 A ILE 290 A ILE 294 A TYR 312 A CYS 315 A ILE 316 A LEU 318 A GLY 319 A TYR 320 |
| N1=C(SCCC1)N[C@H](CC)C(F)(F)F | 6.028 | A VAL 108 A ASP 138 A ASN 141 A MET 142 A TRP 287 A ILE 290 A ILE 294 A TYR 312 A CYS 315 A ILE 316 A GLY 319 A TYR 320 |
| N1=C(SCCC1)Nc1c(OC)ccnc1 | 6.021 | A VAL 108 A THR 111 A ASP 138 A TYR 139 A TRP 287 A ILE 290 A ILE 294 A TYR 312 A ILE 316 A GLY 319 A TYR 320 |
| N1=C(SCCC1)N[C@H](C(CC)CC)C | 6.021 | A VAL 108 A THR 111 A GLN 115 A ASP 138 A TYR 139 A ASN 141 A MET 142 A TRP 287 A ILE 290 A ILE 294 A TYR 312 A ILE 316 A GLY 319 A TYR 320 A SER 323 |
| N1=C(SCCC1)CN([C@@H]1CCN(C1)C)C | 6.02 | A VAL 108 A THR 111 A PHE 114 A GLN 115 A VAL 118 A TRP 124 A VAL 134 A ASP 138 A ASN 141 A TRP 287 A ILE 290 A ILE 316 A GLY 319 A TYR 320 |
| N1=C(SCCC1)NCc1sccc1Br | 6.005 | A VAL 108 A THR 111 A GLN 115 A VAL 134 A LEU 135 A ASP 138 A TRP 287 A ILE 290 A TYR 312 A ILE 316 A GLY 319 A TYR 320 |
| N1=C(SCCC1)N[C@@H](C(C)C)c1sccc1 | 6.002 | A VAL 108 A THR 111 A GLN 115 A VAL 134 A ASP 138 A ASN 141 A MET 142 A TRP 287 A ILE 290 A ILE 294 A TYR 312 A ILE 316 A GLY 319 A TYR 320 |
| N1=C(SCCC1)N[C@@H](CCC=C)C | 5.999 | A VAL 108 A THR 111 A GLN 115 A VAL 134 A ASP 138 A ASN 141 A TRP 287 A ILE 290 A CYS 315 A ILE 316 A GLY 319 A TYR 320 |
| N1=C(SCCC1)NC1(C2CC2)CC1 | 5.996 | A VAL 108 A THR 111 A GLN 115 A VAL 134 A ASP 138 A ASN 141 A MET 142 A TRP 287 A ILE 290 A TYR 312 A ILE 316 A GLY 319 A TYR 320 |
| N1=C(SCCC1)NCc1ccsc1 | 5.993 | A VAL 108 A THR 111 A PHE 114 A GLN 115 A TRP 124 A VAL 134 A LEU 135 A ASP 138 A TRP 287 A ILE 290 A CYS 315 A ILE 316 A GLY 319 A TYR 320 |
| N1=C(SCCC1)Oc1c(CN)cccc1Cl | 5.987 | A ASP 138 A TYR 139 A MET 142 A LYS 227 A VAL 230 A TRP 287 A ILE 290 A HIS 291 A ILE 294 A GLU 297 A TYR 312 A ILE 316 |
| N1=C(SCCC1)Cc1ccncc1Br | 5.985 | A VAL 108 A THR 111 A GLN 115 A VAL 134 A LEU 135 A ILE 137 A ASP 138 A TRP 287 A ILE 290 A TYR 312 A ILE 316 A GLY 319 A TYR 320 |
| N1=C(SCCC1)N[C@@H](C(C)C)CC | 5.983 | A VAL 108 A THR 111 A ASP 138 A TYR 139 A ASN 141 A MET 142 A TRP 287 A ILE 290 A ILE 294 A TYR 312 A CYS 315 A ILE 316 A GLY 319 A TYR 320 |
| N1=C(SCCC1)N[C@H](c1sccc1)C | 5.958 | A VAL 108 A THR 111 A GLN 115 A TRP 124 A VAL 134 A ASP 138 A TRP 287 A ILE 290 A TYR 312 A CYS 315 A ILE 316 A GLY 319 A TYR 320 |
| N1=C(SCCC1)N[C@H](CN)C(C)C | 5.946 | A VAL 108 A THR 111 A GLN 115 A VAL 134 A ASP 138 A ASN 141 A MET 142 A TRP 287 A ILE 290 A ILE 316 A GLY 319 A TYR 320 |
| N1=C(SCCC1)N[C@@H]1[C@H](NCCC1)C | 5.943 | A VAL 108 A THR 111 A GLN 115 A VAL 134 A ILE 137 A ASP 138 A TRP 287 A ILE 290 A TYR 312 A ILE 316 A GLY 319 A TYR 320 |
| N1=C(SCCC1)N(CC)c1c(N)cccc1 | 5.942 | A THR 111 A PHE 114 A GLN 115 A VAL 118 A TRP 124 A VAL 134 A LEU 135 A ASP 138 A GLU 209 A CYS 210 A TYR 312 A TYR 313 A TYR 320 |
| N1=C(SCCC1)N[C@@H](C(C)C)C(=O)N | 5.918 | A VAL 108 A ASP 138 A MET 142 A TRP 287 A ILE 290 A ILE 294 A TYR 312 A CYS 315 A ILE 316 A GLY 319 A TYR 320 |
| N1=C(SCCC1)N[C@@H]([C@H]1CCOC1)C | 5.918 | A VAL 108 A ASP 138 A MET 142 A TRP 287 A ILE 290 A ILE 294 A TYR 312 A CYS 315 A ILE 316 A GLY 319 A TYR 320 |
| N1=C(SCCC1)N[C@@H]1[C@H](OCC1)CC | 5.891 | A VAL 108 A ASP 138 A MET 142 A VAL 230 A TRP 287 A ILE 290 A HIS 291 A ILE 294 A TYR 312 A CYS 315 A ILE 316 A GLY 319 A TYR 320 |
| N1=C(SCCC1)N[C@H]1CCNC1(C)C | 5.88 | A VAL 108 A ASP 138 A TYR 139 A MET 142 A VAL 230 A TRP 287 A ILE 290 A ILE 294 A TYR 312 A CYS 315 A ILE 316 A GLY 319 A TYR 320 |
| N1=C(SCCC1)[C@H]1NCCc2c1scc2 | 5.865 | A ASP 138 A TYR 139 A MET 142 A LYS 227 A VAL 230 A TRP 287 A ILE 290 A HIS 291 A ILE 294 A TYR 312 A ILE 316 |
| N1=C(SCCC1)Nc1c(csc1)C | 5.863 | A VAL 108 A THR 111 A GLN 115 A VAL 134 A ASP 138 A TRP 287 A ILE 290 A TYR 312 A CYS 315 A ILE 316 A GLY 319 A TYR 320 |
| N1=C(SCCC1)N[C@@H](C)c1sccn1 | 5.857 | A THR 111 A GLN 115 A VAL 134 A LEU 135 A ASP 138 A TRP 287 A ILE 290 A TYR 312 A CYS 315 A ILE 316 A GLY 319 A TYR 320 |
| N1=C(SCCC1)NC1CCOCC1 | 5.853 | A VAL 108 A ASP 138 A ASN 141 A MET 142 A VAL 230 A TRP 287 A ILE 290 A ILE 294 A TYR 312 A CYS 315 A ILE 316 A GLY 319 A TYR 320 |
| N1=C(SCCC1)N1[C@H]2[C@H](SCC1)CCCC2 | 5.843 | A LEU 135 A ASP 138 A TYR 139 A MET 142 A SER 211 A LEU 212 A LYS 227 A VAL 230 A TRP 287 A ILE 290 A ILE 294 A TYR 312 A ILE 316 |
| N1=C(SCCC1)CN1[C@H](CCC[C@H]1C)C | 5.839 | A VAL 108 A THR 111 A GLN 115 A TRP 124 A VAL 134 A LEU 135 A ASP 138 A TRP 287 A ILE 290 A TYR 312 A ILE 316 A GLY 319 A TYR 320 |
| N1=C(SCCC1)N(Cc1sccc1C)C | 5.834 | A VAL 108 A THR 111 A GLN 115 A VAL 134 A LEU 135 A ASP 138 A TRP 287 A ILE 290 A TYR 312 A ILE 316 A GLY 319 A TYR 320 |
| N1=C(SCCC1)N1C[C@@H]2N[C@@H](C2)C1 | 5.822 | A VAL 108 A THR 111 A GLN 115 A VAL 134 A ASP 138 A ASN 141 A TRP 287 A ILE 290 A ILE 316 A GLY 319 A TYR 320 |
| N1=C(SCCC1)N(c1c(CC)cccc1)C | 5.813 | A THR 111 A PHE 114 A GLN 115 A VAL 118 A TRP 124 A VAL 134 A LEU 135 A ASP 138 A CYS 210 A TYR 312 A ILE 316 A TYR 320 |
| N1=C(SCCC1)N[C@H]([C@H](CC)C)C(=O)OC | 5.811 | A VAL 108 A THR 111 A GLN 115 A VAL 134 A ASP 138 A MET 142 A TRP 287 A ILE 290 A ILE 294 A TYR 312 A CYS 315 A ILE 316 A GLY 319 A TYR 320 |
| N1=C(SCCC1)NC(C(C)C)C(C)C | 5.809 | A VAL 108 A ASP 138 A TYR 139 A MET 142 A TRP 287 A ILE 290 A HIS 291 A ILE 294 A TYR 312 A CYS 315 A ILE 316 A GLY 319 A TYR 320 |
| N1=C(SCCC1)N[C@H](CC)c1sccc1 | 5.805 | A VAL 108 A THR 111 A PHE 114 A GLN 115 A TRP 124 A VAL 134 A LEU 135 A ASP 138 A CYS 210 A SER 211 A ILE 316 A TYR 320 |
| N1=C(SCCC1)Cc1c(Cl)cncc1Cl | 5.787 | A VAL 108 A THR 111 A GLN 115 A VAL 134 A LEU 135 A ASP 138 A TRP 287 A ILE 290 A TYR 312 A ILE 316 A GLY 319 A TYR 320 |
| N1=C(SCCC1)N[C@H]1[C@H](SCC1)C | 5.782 | A VAL 108 A ASP 138 A TYR 139 A MET 142 A TRP 287 A ILE 290 A ILE 294 A TYR 312 A CYS 315 A ILE 316 A GLY 319 A TYR 320 |
| N1=C(SCCC1)N/C=C(/CC)C | 5.761 | A VAL 108 A THR 111 A GLN 115 A VAL 134 A ASP 138 A TRP 287 A ILE 290 A TYR 312 A CYS 315 A ILE 316 A GLY 319 A TYR 320 |
| N1=C(SCCC1)N[C@@H](C)C(=O)N | 5.748 | A THR 111 A PHE 114 A GLN 115 A ASP 138 A TRP 287 A ILE 290 A TYR 312 A ILE 316 A GLY 319 A TYR 320 |
| N1=C(SCCC1)N[C@H]1CC[S@@](=O)C1 | 5.742 | A VAL 108 A ASP 138 A TYR 139 A MET 142 A TRP 287 A ILE 290 A ILE 294 A TYR 312 A CYS 315 A ILE 316 A GLY 319 A TYR 320 |
| N1=C(SCCC1)N[C@](C(C)C)(C)C#N | 5.7 | A VAL 108 A ASP 138 A TYR 139 A MET 142 A TRP 287 A ILE 290 A ILE 294 A TYR 312 A CYS 315 A ILE 316 A GLY 319 A TYR 320 |
| N1=C(SCCC1)Nc1csnc1C | 5.697 | A VAL 108 A THR 111 A GLN 115 A VAL 134 A ASP 138 A TRP 287 A ILE 290 A TYR 312 A ILE 316 A GLY 319 A TYR 320 |
| N1=C(SCCC1)N[C@@H](C(C)C)C(=O)OC | 5.672 | A VAL 108 A THR 111 A PHE 114 A GLN 115 A VAL 134 A ASP 138 A ASN 141 A TRP 287 A TYR 312 A ILE 316 A GLY 319 A TYR 320 |
| N1=C(SCCC1)NC1CCSCC1 | 5.671 | A VAL 108 A ASP 138 A MET 142 A VAL 230 A TRP 287 A ILE 290 A ILE 294 A TYR 312 A CYS 315 A ILE 316 A GLY 319 A TYR 320 |
| N1=C(SCCC1)N[C@@H](CCCN)C | 5.669 | A VAL 108 A THR 111 A GLN 115 A VAL 134 A LEU 135 A ASP 138 A ASN 141 A TRP 287 A ILE 290 A CYS 315 A ILE 316 A LEU 318 A GLY 319 A TYR 320 |
| N1=C(SCCC1)[C@H]1NCCc2sccc12 | 5.667 | A LEU 135 A ASP 138 A TYR 139 A MET 142 A SER 211 A LEU 212 A LYS 227 A VAL 230 A ILE 290 A HIS 291 A ILE 294 A TYR 312 A ILE 316 |
| N1=C(SCCC1)N[C@@H](C(O)(C)C)C(C)C | 5.651 | A VAL 108 A THR 111 A GLN 115 A VAL 118 A TRP 124 A VAL 134 A LEU 135 A ASP 138 A CYS 210 A SER 211 A TYR 312 A ILE 316 A TYR 320 |
| N1=C(SCCC1)N[C@@H]([C@@H](CO)C)C | 5.631 | A VAL 108 A ASP 138 A TYR 139 A ASN 141 A MET 142 A TRP 287 A ILE 290 A ILE 294 A TYR 312 A CYS 315 A ILE 316 A GLY 319 A TYR 320 |
| N1=C(SCCC1)N[C@H](C)C(=O)C | 5.617 | A VAL 108 A THR 111 A GLN 115 A VAL 134 A ASP 138 A TRP 287 A ILE 290 A TYR 312 A ILE 316 A GLY 319 A TYR 320 |
| N1=C(SCCC1)N[C@H]1CCN(C1)C | 5.605 | A VAL 108 A THR 111 A GLN 115 A VAL 134 A ASP 138 A ASN 141 A TRP 287 A ILE 290 A CYS 315 A ILE 316 A GLY 319 A TYR 320 |
| N1=C(SCCC1)N[C@H](CC)C(=O)N | 5.604 | A VAL 108 A THR 111 A GLN 115 A ASP 138 A TRP 287 A ILE 290 A TYR 312 A ILE 316 A GLY 319 A TYR 320 |
| N1=C(SCCC1)N[C@H](C(C)C)C | 5.592 | A VAL 108 A THR 111 A GLN 115 A VAL 134 A ASP 138 A TRP 287 A ILE 290 A TYR 312 A CYS 315 A ILE 316 A GLY 319 A TYR 320 |
| N1=C(SCCC1)N1CCSC[C@@H](C1)C | 5.536 | A VAL 108 A THR 111 A PHE 114 A GLN 115 A VAL 118 A TRP 124 A VAL 134 A LEU 135 A ASP 138 A TYR 312 A ILE 316 A TYR 320 |
| N1=C(SCCC1)[C@@H](N)c1sccc1 | 5.534 | A VAL 108 A THR 111 A GLN 115 A VAL 134 A LEU 135 A ILE 137 A ASP 138 A TRP 287 A ILE 290 A TYR 312 A ILE 316 A GLY 319 A TYR 320 |
| N1=C(SCCC1)N1[C@@H]([C@H](SCC1)C)C | 5.514 | A THR 111 A PHE 114 A GLN 115 A TRP 124 A VAL 134 A LEU 135 A ASP 138 A ILE 290 A TYR 312 A ILE 316 A TYR 320 |
| N1=C(SCCC1)[C@@H](CN)c1sccc1 | 5.404 | A VAL 108 A THR 111 A GLN 115 A ASP 138 A ASN 141 A TRP 287 A ILE 290 A ILE 294 A TYR 312 A ILE 316 A GLY 319 A TYR 320 |
| N1=C(SCCC1)[C@@H]1[C@@H](N(CC1)C)C | 5.232 | A VAL 108 A ASP 138 A MET 142 A TRP 287 A ILE 290 A ILE 294 A TYR 312 A CYS 315 A ILE 316 A GLY 319 A TYR 320 |
| N1=C(SCCC1)N[C@@H](CN(C)C)C | 5.125 | A VAL 108 A ASP 138 A TYR 139 A MET 142 A VAL 230 A TRP 287 A ILE 290 A HIS 291 A ILE 294 A TYR 312 A ILE 316 A GLY 319 A TYR 320 |
| N1=C(SCCC1)N[C@H](CF)C | 5.12 | A VAL 108 A THR 111 A GLN 115 A VAL 134 A ASP 138 A ASN 141 A TRP 287 A ILE 316 A GLY 319 A TYR 320 |
